# Supplementary material for: Spontaneous exciton dissociation enables spin state interconversion in delayed fluorescence organic semiconductors
Source: Nat Commun. 2021 Nov 17;12:6640. doi: 10.1038/s41467-021-26689-8 (PMC8599618; doi:10.1038/s41467-021-26689-8)
Supplement: Supplementary file 1 — Supplementary Information [file 41467_2021_26689_MOESM1_ESM.pdf]

Supplementary Information for

Spontaneous exciton dissociation enables spin state interconversion in delayed fluorescence organic semiconductors

Alexander J. Gillett[1], Claire Tonnelé[2], Giacomo Londi[3], Gaetano Ricci[4], Manon Catherin[5], Darcy M. L. Unson[1], David Casanova[2], Frédéric Castet[6], Yoann Olivier[4], Weimin M. Chen[7], Elena Zaborova[5], Emrys W. Evans[1,8], Bluebell H. Drummond[1], Patrick J. Conaghan[1,9], Lin-Song Cui[1,10], Neil C. Greenham[1], Yuttapoom Puttisong[7]*, Frédéric Fages[5]*, David Beljonne[3]* and Richard H. Friend[1]*.*

[1]Cavendish Laboratory, University of Cambridge, JJ Thomson Avenue, Cambridge, UK.

[2]Donostia International Physics Centre (DIPC), Donostia, Euskadi, Spain.

[3]Laboratory for Chemistry of Novel Materials, Université de Mons, Place du Parc 20, 7000 Mons, Belgium.

[4]Unité de Chimie Physique Théorique et Structurale & Laboratoire de Physique du Solide, Namur Institute of Structured Matter, Université de Namur, B-5000 Namur, Belgium.

[5]Aix Marseille Univ, CNRS, CINaM UMR 7325, AMUtech, Campus de Luminy, 13288 Marseille, France.

[6]Institut des Sciences Moléculaires, Université de Bordeaux, 33405 Talence, France.

[7]Department of Physics, Chemistry and Biology (IFM) Linköping University, Linköping, Sweden.

[8]Department of Chemistry, Swansea University, Singleton Park, Swansea, UK.

[9]ARC Centre of Excellence in Exciton Science, School of Chemistry, University of Sydney, NSW 2006, Australia.

[10]Department of Polymer Science and Engineering, University of Science and Technology of China, Hefei, Anhui 230026, China.

[*]Corresponding authors: Alexander J. Gillett: E-mail: ajg216@cam.ac.uk; Yuttapoom Puttisong: E-mail: yuttapoom.puttisong@liu.se; Frédéric Fages: E-mail frederic.fages@univ-amu.fr; David Beljonne: E-mail: david.beljonne@umons.ac.be; Richard H. Friend: E-mail: rhf10@cam.ac.uk.

Table of Contents

Material properties **4**

Attenuation coefficients and ΔE_{ST} 4

BF2 low temperature fluorescence and phosphorescence 6

BF2 Absorption and PL 7

Transient absorption of BF2:PCBM **8**

OSC device data of BF2:PCBM **9**

Neat BF2 and 4CzIPN OSC device data **10**

BF2 diluted in CBP **11**

Steady-state PL spectra of BF2 dilution series 12

Ultrafast TA of BF2 in CBP 13

Nanosecond TA of BF2 in CBP 15

Transient PL spectra of BF2 in CBP 18

BF2 diluted in DPEPO **19**

Ultrafast TA of BF2 in DPEPO 20

Nanosecond TA of BF2 in DPEPO 22

PLDMR spectroscopy and hyperfine interactions **23**

PLDMR of BF2 27

PLDMR of APDC-DTPA 29

PLDMR of TXO-TPA 31

PLDMR of 4CzIPN 33

Detailed concentration series of BF2 diluted in CBP **34**

PLQE of BF2 in CBP 34

Ultrafast TA of BF2 dilution series in CBP 36

Transient absorption of neat 4CzIPN **37**

Computational results **38**

Methodology 38

Calculations on BF2 dimer 41

Calculations on BF2 tetramer 50

Calculations on 4CzIPN dimer 59

Calculations on hyperfine couplings in the BF2 dimer 67

Calculations on the hyperfine ISC rates 72

Supplementary References **76**

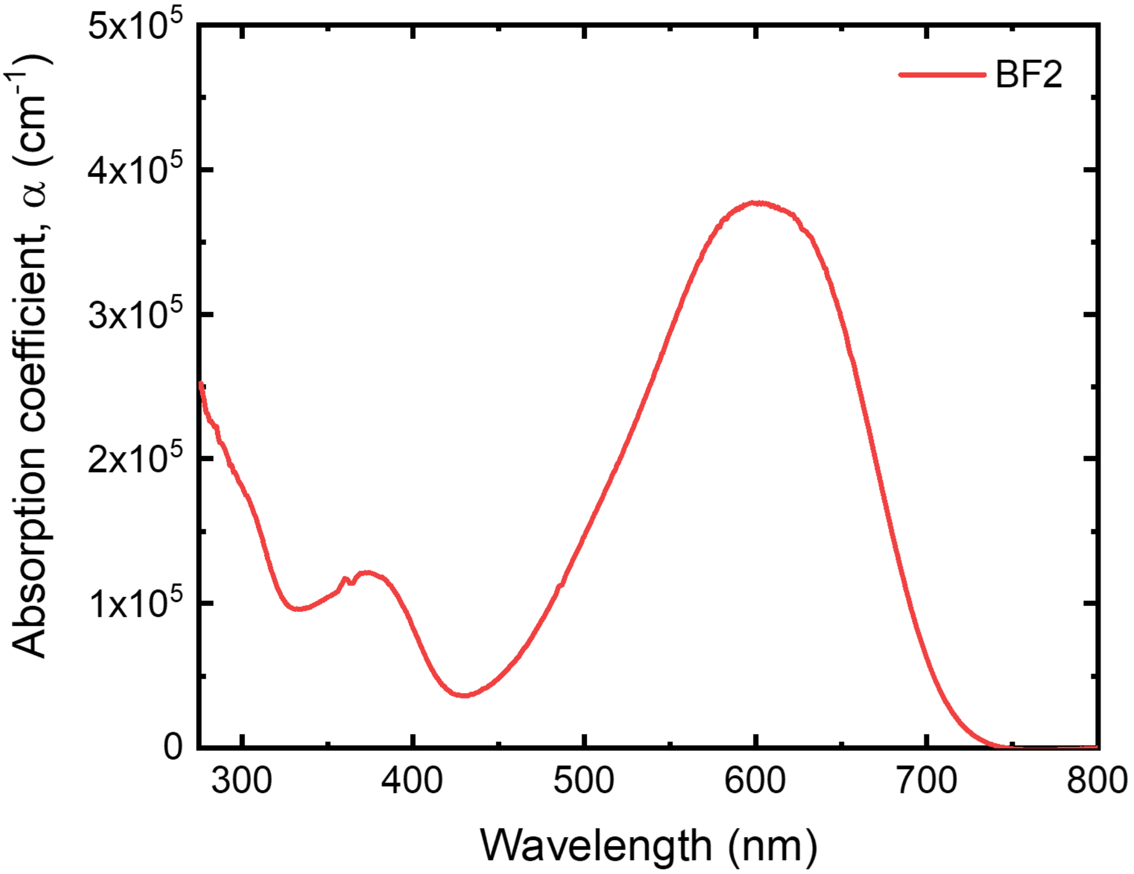

Supplementary Figure 1: The absorption coefficient of a neat BF2 film.

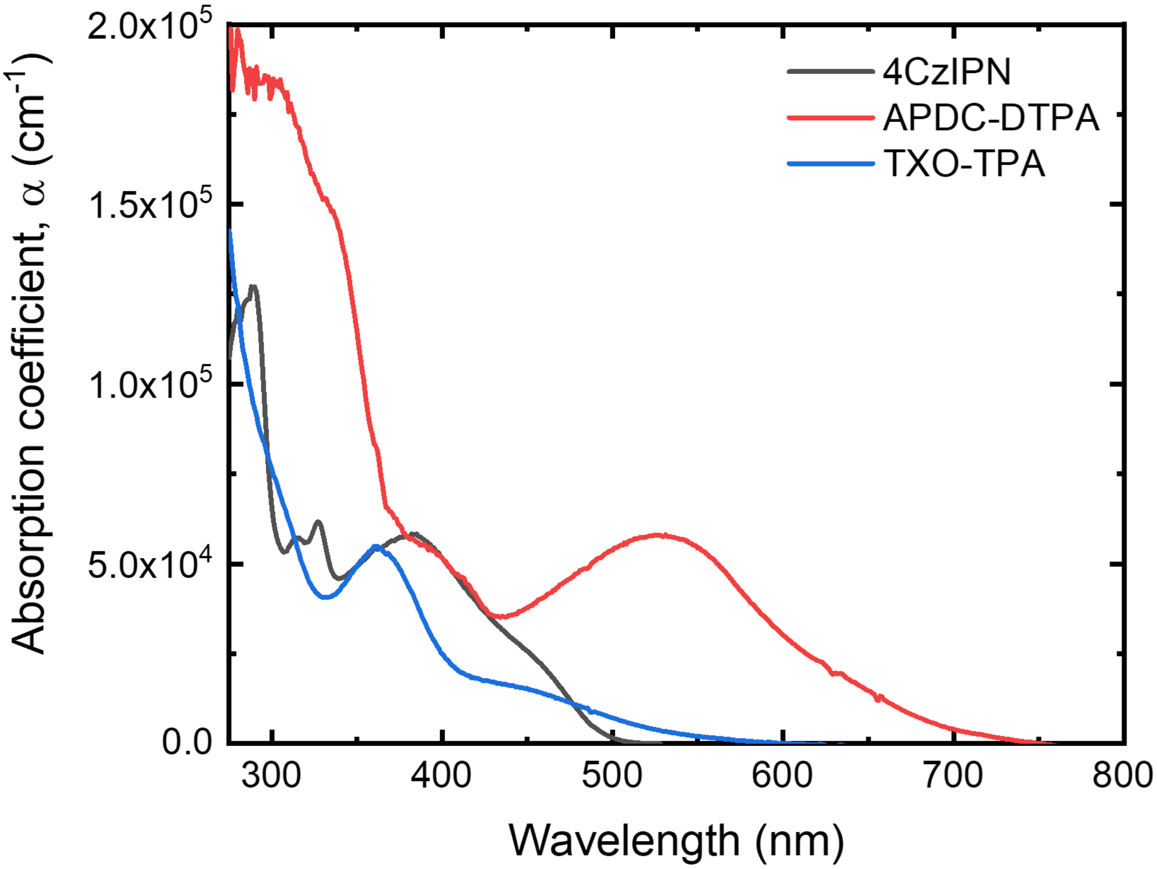

Supplementary Figure 2: The absorption coefficients of neat films of the other organic DF materials investigated.

Material	Absorption coefficient (cm^{-1})	ΔE_{ST} (meV)
BF2	$3.8x10^5$	200
APDC-DTPA	$5.8x10^4$	140[1]
4CzIPN	$2.5x10^4$	40[2]
TXO-TPA	$1.8x10^4$	40[3]

Supplementary Table 1: The absorption coefficients and ΔE_{ST} of organic DF materials investigated in this study. The absorption coefficient is taken from the peak of the lowest energy intra-CT absorption band: BF2 (600 nm); APDC-DTPA (530 nm); 4CzIPN (450 nm); and TXO-TPA (425 nm). The ΔE_{ST} is obtained from low temperature phosphorescence measurements in all cases.

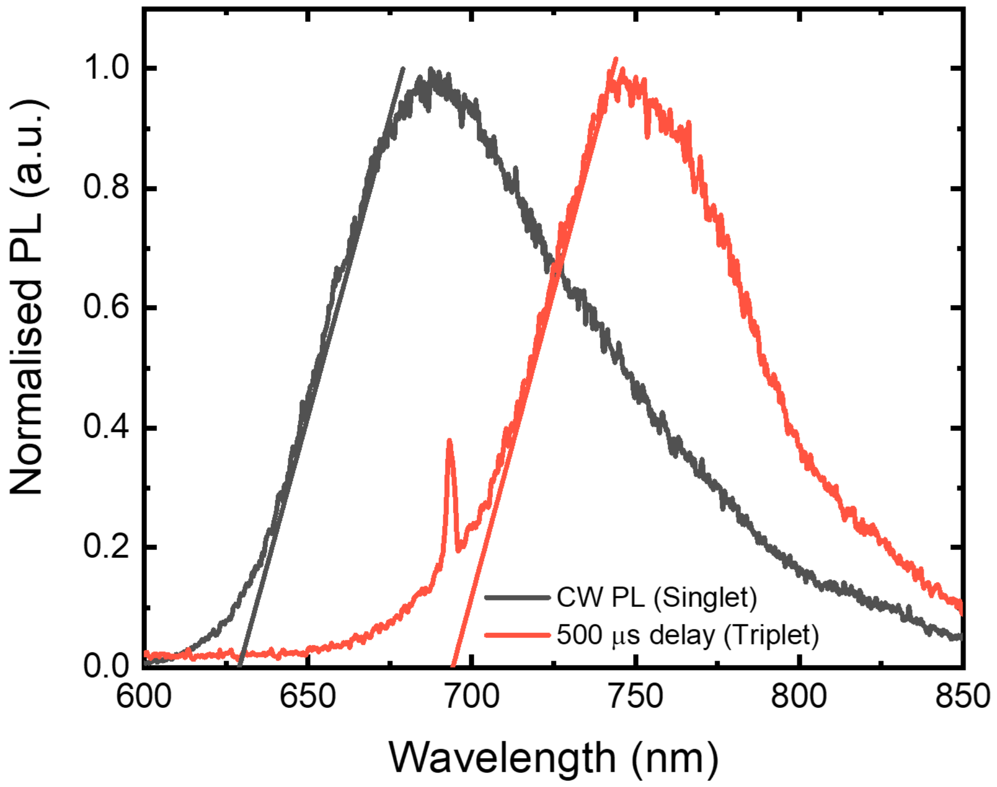

Supplementary Figure 3: The normalised fluorescence and phosphorescence spectra of BF2 doped at 5 wt% in polystyrene, taken at 10 K. Excitation was provided by a 400 nm laser. The onset of the fluorescence is at 1.97 eV, whilst the phosphorescence onset is at 1.77 eV. The difference in onset of the spectra gives a singlet-triplet energy gap of 0.2 eV.

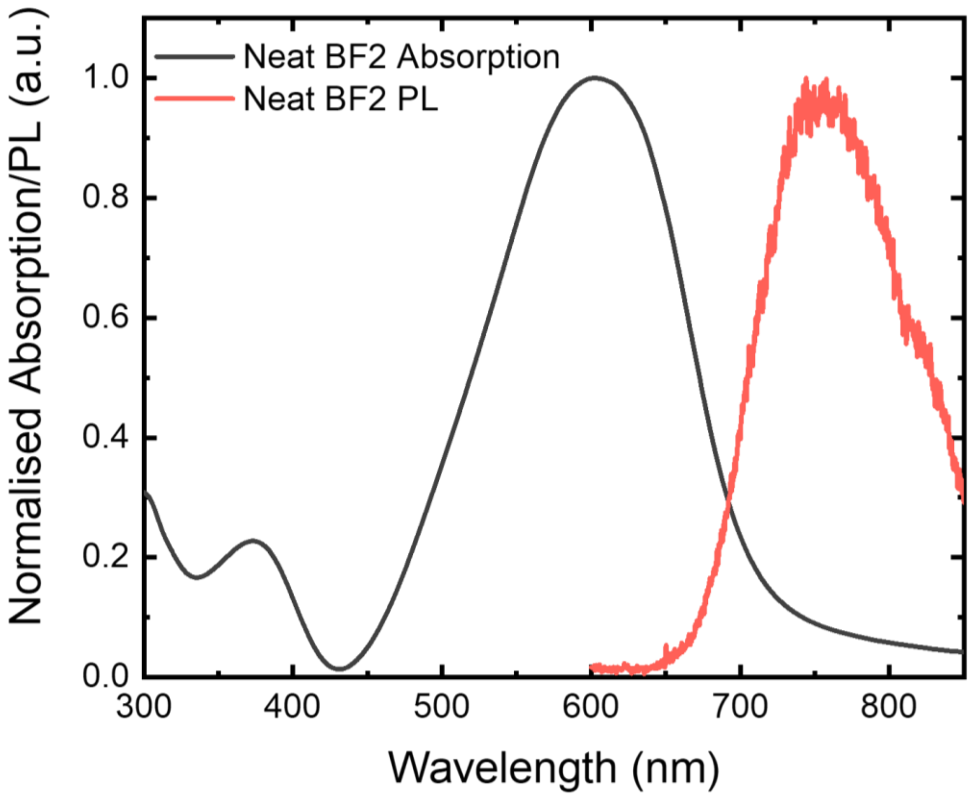

Supplementary Figure 4: The normalised absorption and photoluminescence spectra of a neat BF2 film.

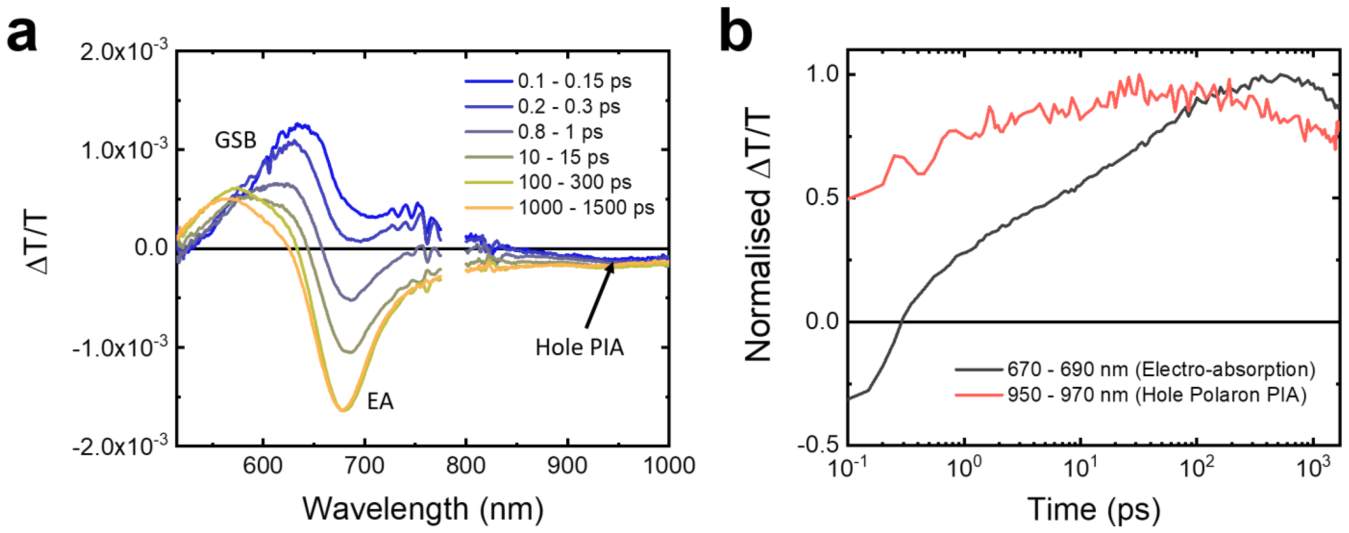

Supplementary Figure 5: TA studies of a BF2: $PC_{60}BM$ film. (a) The TA spectra of a BF2:$PC_{60}BM$ film, excited at 610 nm with a fluence of 3.6 μJ cm^{-2}. By 0.1 – 0.15 ps, the BF2 stimulated emission (SE) is already strongly quenched compared to the neat film TA (Figure 2), suggesting that a significant fraction of the electron transfer from BF2 to $PC_{60}BM$ takes place on ultrafast (<0.1 ps) timescales. A PIA centred at 940 nm that is clearly distinct from the BF2 intra-^{1}CT PIA peaking beyond 1000 nm is also seen at 0.1 – 0.15 ps. Given the rapid quenching of the BF2 intra-^{1}CT, this new PIA is assigned to the hole polaron photo-induced absorption (PIA) of BF2. As time progresses, a sharp new negative feature forms at the band edge of the BF2 absorption around 680 nm. Due to the spectral location at the edge of the BF2 ground state absorption and distinct kinetics to the hole polaron PIA, this new feature is assigned to the electro-absorption (EA) of BF2, induced by the separation of the charge transfer state into free charges[4–7]. The disconnect in the spectral data is due to the gap in our probe range around the 800 nm fundamental of the laser used to run the setup. **(b)** The TA kinetics of the hole polaron (950 – 970 nm) and EA (670 – 690 nm). We note that the kinetics of the formation of the EA feature are clearly distinct to the formation of the hole polaron PIA, confirming that these two features have different origins. We note that the EA grows in more slowly than the hole polaron, as would be expected given that charge transfer must take place before the charge transfer state can dissociate into free charges.

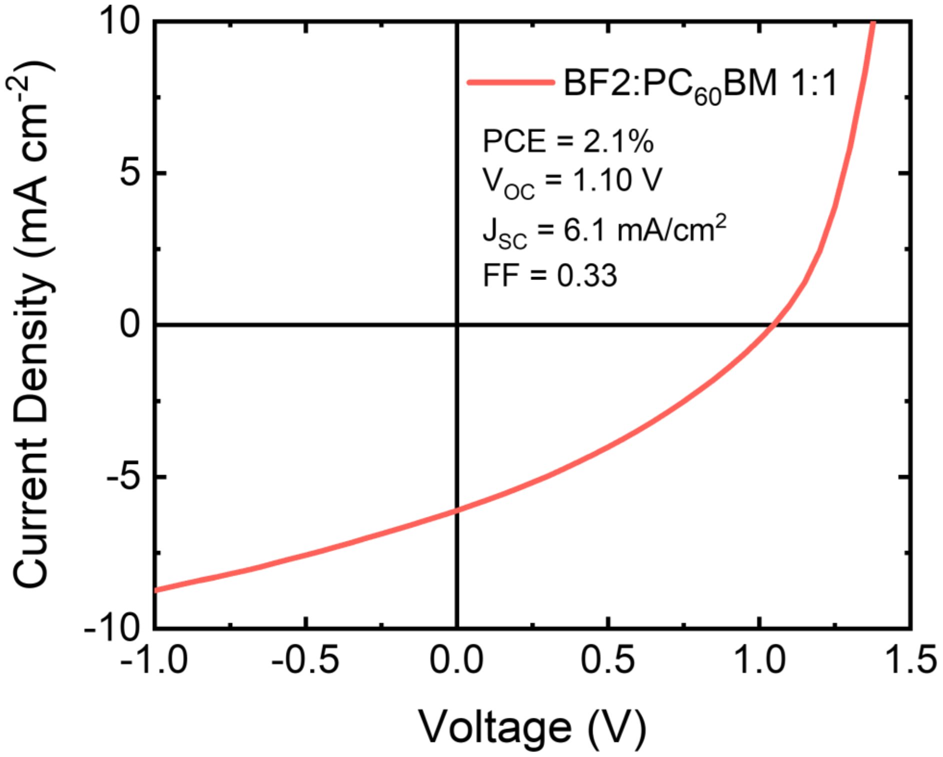

Supplementary Figure 6: The light current density-voltage curve of the corresponding BF2:PC_{60}BM OSC device, taken under 100 mW cm^{-2} AM1.5G illumination. The significant photocurrent generation confirms that electron transfer from BF2 to PC_{60}BM occurs efficiently, supporting the assignment in Supplementary Figure 5 of the PIA centred at 950 nm as the hole polaron of BF2.

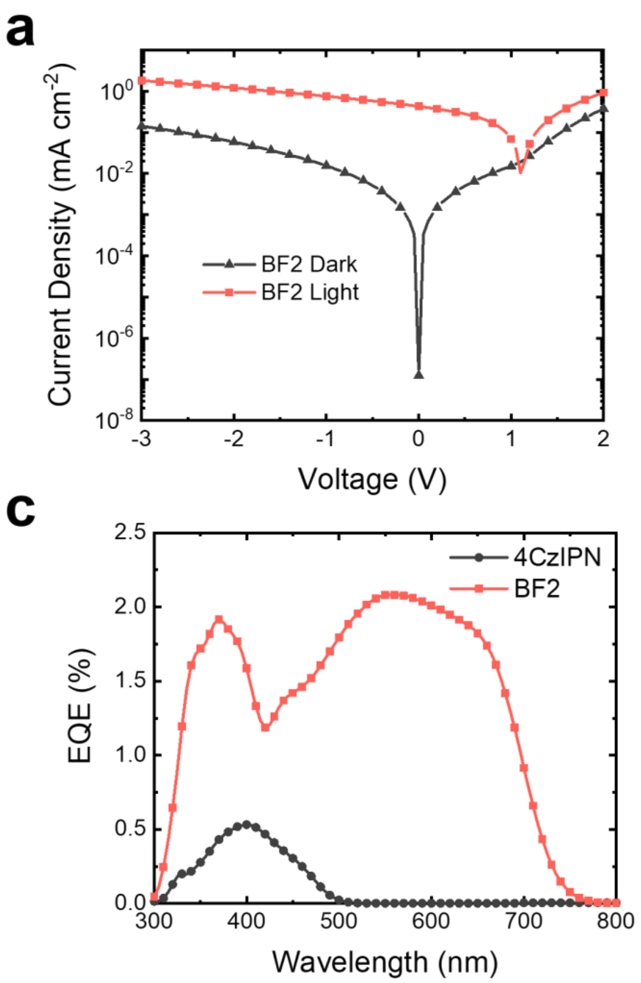

Supplementary Figure 7: Device characteristics of neat BF2 and 4CzIPN OSCs. (a) The dark and light current density-voltage curves under 100 mW cm^{-2} AM1.5G illumination of an OSC device fabricated with a neat BF2 active layer. The current density extracted under reverse bias is greatly enhanced under 1 sun illumination, providing evidence for the photo-generation of bound SCRPs in BF2 that can be dissociated by an external electric field. **(b)** The dark and light current density-voltage curves under 100 mW cm^{-2} AM1.5G illumination of an OSC device fabricated with a neat 4CzIPN active layer. The photocurrent generated from the neat 4CzIPN device is significantly lower than the neat BF2 device. **(c)** The EQE responses of the neat BF2 and 4CzIPN devices taken at short-circuit. The peak EQE of 2.1% at 555 nm in the neat BF2 device demonstrates that BF2 itself can generate a moderate photocurrent without the need for a separate electron or hole acceptor. The lower peak EQE of 0.53% in the neat 4CzIPN device confirms that 4CzIPN alone is not able to generate significant photocurrent.

Device	V_{oc} (V)	J_{sc} (mA cm^{-2})	FF	PCE (%)
Neat BF2	1.12	0.43	0.31	0.15
Neat 4CzIPN	0.98	0.02	0.22	0.004

Supplementary Table 2: The summarised performance metrics of the neat BF2 and 4CzIPN OSC devices under 100 mW cm^{-2} AM1.5G illumination.

BF2 diluted in CBP

To gain a deeper insight into the exciton dissociation process, we have investigated the ultrafast TA of BF2 dispersed in the non-interacting host CBP at 2, 10 and 40 wt% (Supplementary Figure 9). We note that the photoluminescence (PL) spectra of BF2 broadens and undergoes a significant red shift as a function of doping concentration (Supplementary Figure 8). We attribute this emission broadening and red shift to two factors: 1. As BF2 has a significantly larger dipole moment than the CBP host, as the BF2 concentration increases, the intra-^{1}CT excitons will be progressively more stabilised by the surrounding BF2 molecules, leading to a red shift in the PL maxima[8]; 2. We find that in the BF2 dimer, there is significant delocalisation of the bright state wavefunction over the neighbouring molecule that could result in a broadening and red shift in the observed emission (Supplementary table 7). This can be attributed to the tendency of BF2 to form dimers in low wt% films, followed by larger aggregates at a higher wt%[9]. Thus, we consider the 2 wt% film to represent the behaviour of isolated BF2 molecules, with higher loadings increasingly reflecting the properties of aggregated BF2. In the ultrafast TA of the BF2 dilution series, we observe a dramatic shortening of the SE lifetime with increasing BF2 concentration (Figs. 4b and S10); this is consistent with the expectation that closer contact between BF2 molecules will result in faster exciton dissociation. Of particular interest is the nanosecond TA of the BF2 dilution series (Supplementary Figure 11). In the 2 wt% film, the SE decays within 10 ns, leaving behind a long-lived GSB signal that represents the small fraction of BF2 intra-^{1}CT excitons that undergo ISC to the T_1 state. The kinetics of the GSB decay can be well-described by a bi-exponential function, typical of DF emitters[10,11]. The short 'prompt' time constant of 2.4 ns primarily represents the radiative decay of singlet excitons (Supplementary Figure 14), whilst the long

‘delayed’ time constant of 260 μs confirms that the rISC process of isolated BF2 is particularly slow, as expected for the relatively large ΔE_{ST} of 0.2 eV. In contrast, the 10 and 40 wt% films exhibit markedly different behaviour. After efficient dissociation of the excitons quenches the SE on picosecond timescales, we observe the regrowth of the SE on microsecond timescales (Figs. 2c, 2d, S11f and S12c). This suggests that the emissive intra-^{1}CT exciton is being reformed from the separated charge carriers. We verify that the emissive state present on microsecond timescales has the same PL spectrum as the steady-state emission (Supplementary Figure 15), confirming that the SE regrowth observed in the TA is due to the reformation of the bright intra-^{1}CT exciton.

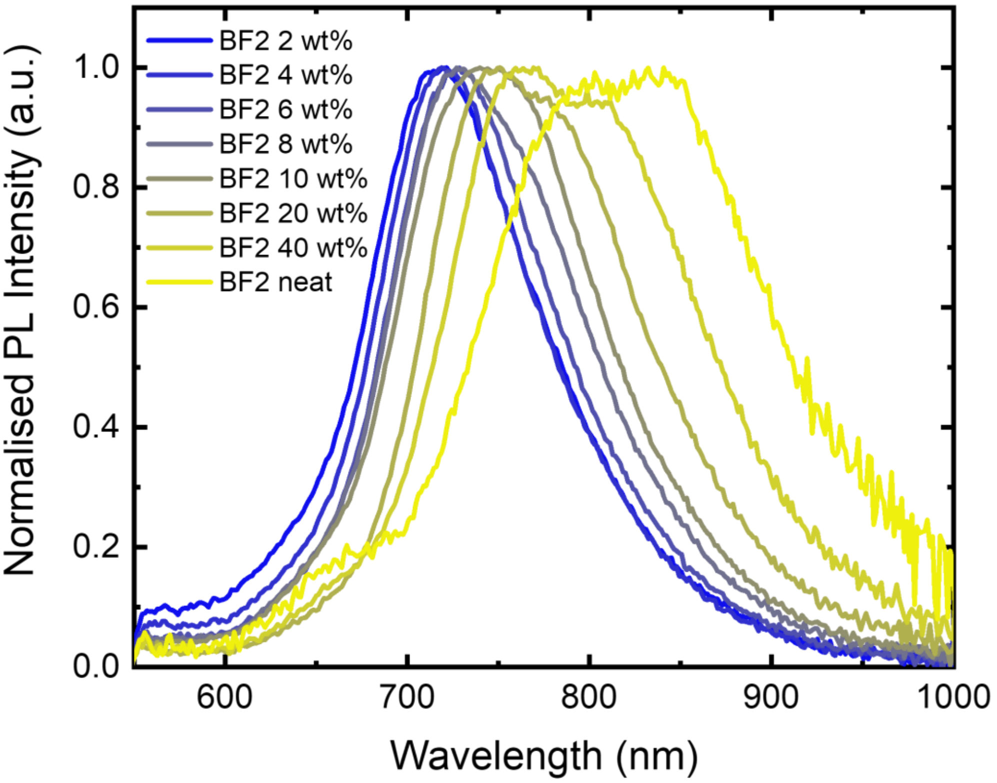

Supplementary Figure 8: The normalised PL spectra of BF2 at 2, 4, 6, 8, 10, 20, 40 wt% in CBP and a neat film. Excitation was provided by a 520 nm laser. There is a clear red shift of the PL maximum and broadening of the spectra as the doping concentration rises. The PL spectra were extracted from the PLQE measurements reported in Supplementary Table 3.

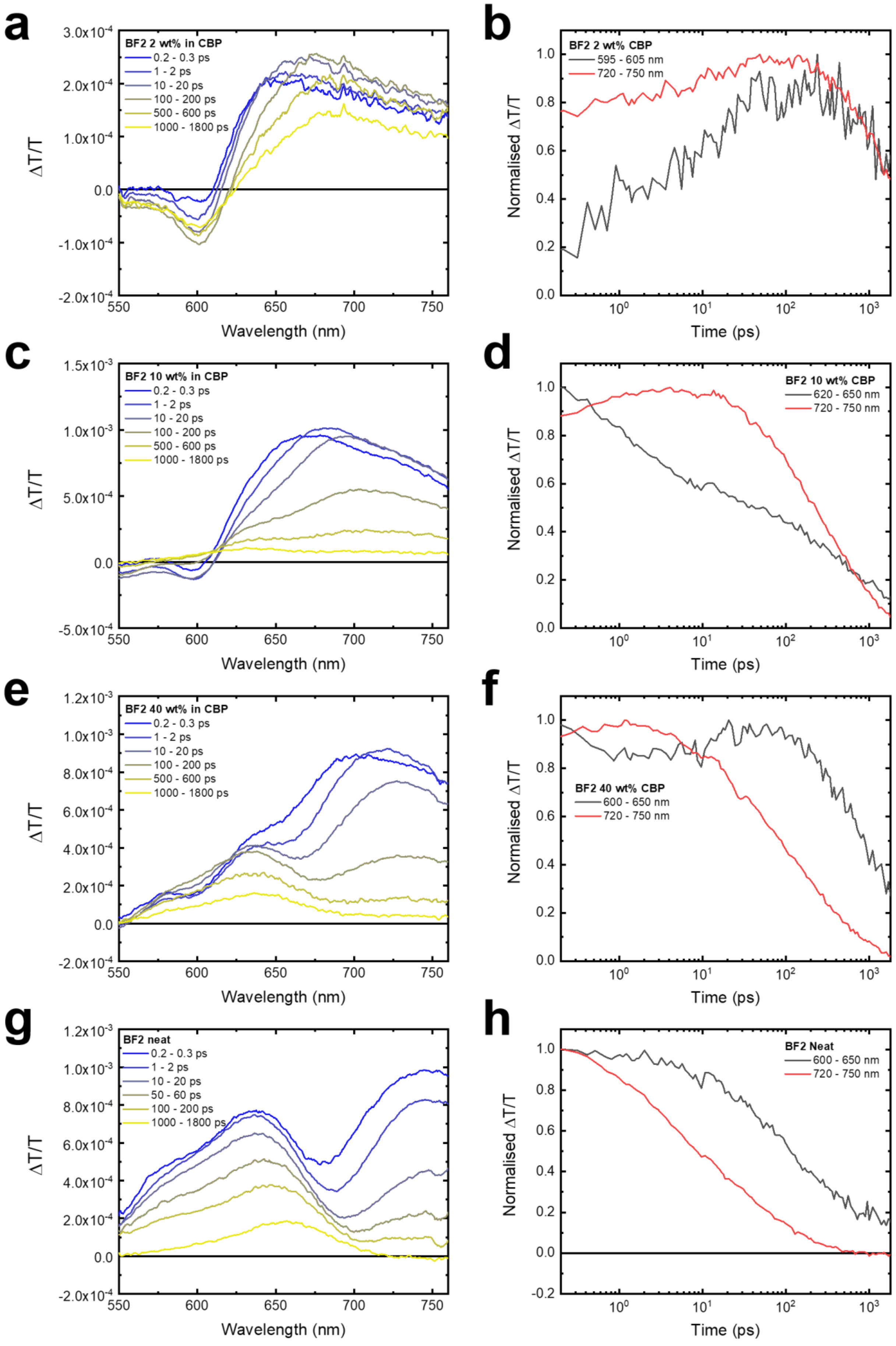
a
BF2 2 wt% in CBP
0.2 - 0.3 ps
1 - 2 ps
10 - 20 ps
100 - 200 ps
500 - 600 ps
1000 - 1800 ps
ΔT/T
Wavelength (nm)
b
BF2 2 wt% CBP
595 - 605 nm
720 - 750 nm
Normalised ΔT/T
Time (ps)
c
BF2 10 wt% in CBP
0.2 - 0.3 ps
1 - 2 ps
10 - 20 ps
100 - 200 ps
500 - 600 ps
1000 - 1800 ps
ΔT/T
Wavelength (nm)
d
BF2 10 wt% CBP
620 - 650 nm
720 - 750 nm
Normalised ΔT/T
Time (ps)
e
BF2 40 wt% in CBP
0.2 - 0.3 ps
1 - 2 ps
10 - 20 ps
100 - 200 ps
500 - 600 ps
1000 - 1800 ps
ΔT/T
Wavelength (nm)
f
BF2 40 wt% CBP
600 - 650 nm
720 - 750 nm
Normalised ΔT/T
Time (ps)
g
BF2 neat
0.2 - 0.3 ps
1 - 2 ps
10 - 20 ps
50 - 60 ps
100 - 200 ps
1000 - 1800 ps
ΔT/T
Wavelength (nm)
h
BF2 Neat
600 - 650 nm
720 - 750 nm
Normalised ΔT/T
Time (ps)

Supplementary Figure 9: The ultrafast TA spectra and kinetics of BF2 at 2, 10, and 40 wt% in CBP and a neat BF2 film. (a) The TA spectrum of BF2 at 2 wt% in CBP, excited at 532 nm with a fluence of 15.7 μJ cm^{-2}. **(b)** The corresponding TA kinetics for the BF2 at 2 wt% in CBP film. **(c)** The TA spectrum of BF2 at 10 wt% in CBP, excited at 532 nm with a fluence of 15.7 μJ cm^{-2}. **(d)** The corresponding TA kinetics for the BF2 at 10 wt% in CBP film. **(e)** The TA spectrum of BF2 at 40 wt% in CBP, excited at 532 nm with a fluence of 6.3 μJ cm^{-2}. **(f)** The corresponding TA kinetics for the BF2 at 40 wt% in CBP film. **(g)** The TA spectrum of the neat BF2 film, excited at 532 nm with a fluence of 5.3 μJ cm^{-2}. **(h)** The corresponding TA kinetics for the neat BF2 film.

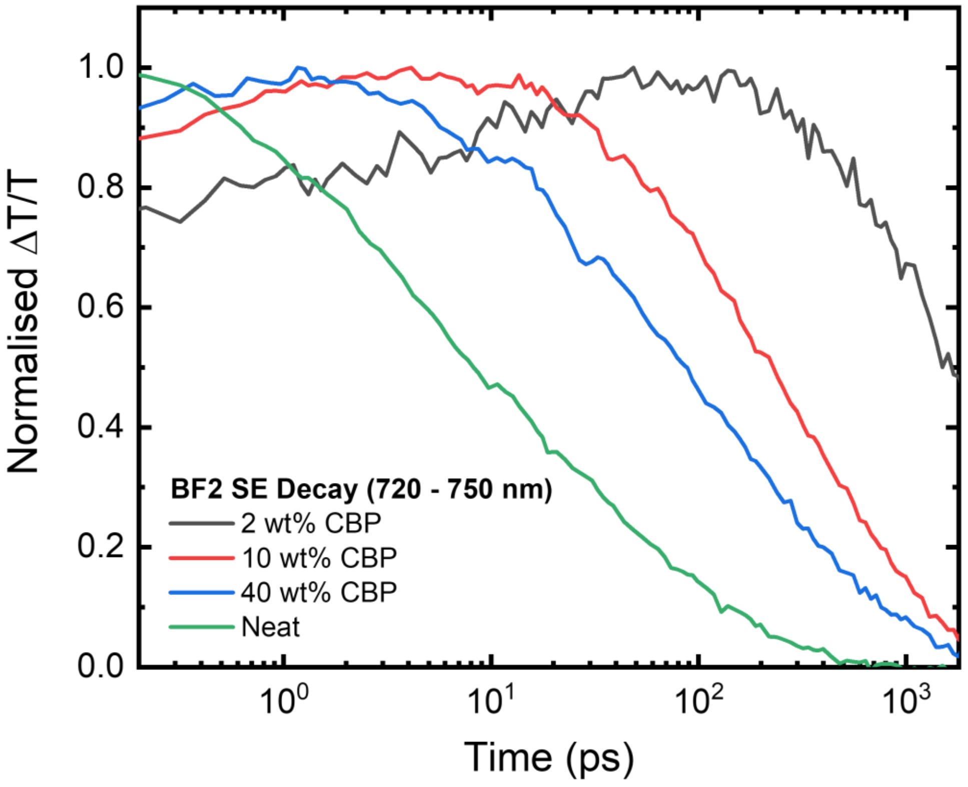

Supplementary Figure 10: The normalised ultrafast TA kinetics of the BF2 SE (720 – 750 nm) for the 2, 10, and 40 wt% films in CBP and the neat BF2 film. The lifetime of the SE falls rapidly as the doping concentration is increased, confirming that the increased contact between BF2 molecules results in faster intermolecular charge transfer. The slight increase in the intensity of the SE intensity between 720 – 750 nm in the 2 wt% blend within the first 100 ps is due to a red shift in the SE band over these timescales.

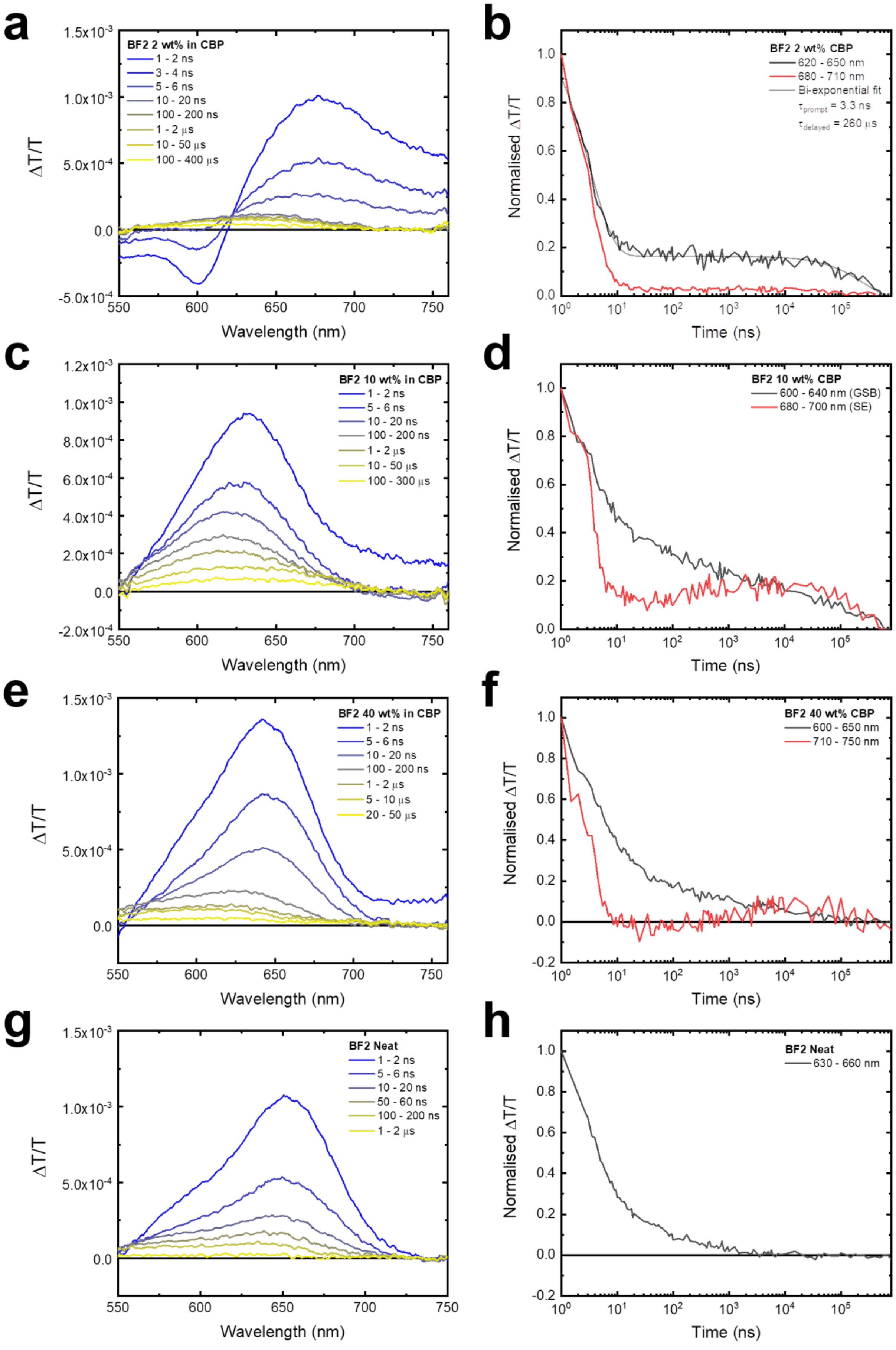

a
BF2 2 wt% in CBP
1 - 2 ns
3 - 4 ns
5 - 6 ns
10 - 20 ns
100 - 200 ns
1 - 2 μs
10 - 50 μs
100 - 400 μs
ΔT/T
Wavelength (nm)
b
BF2 2 wt% CBP
620 - 650 nm
680 - 710 nm
Bi-exponential fit
τprompt = 3.3 ns
τdelayed = 260 μs
Normalised ΔT/T
Time (ns)
c
BF2 10 wt% in CBP
1 - 2 ns
5 - 6 ns
10 - 20 ns
100 - 200 ns
1 - 2 μs
10 - 50 μs
100 - 300 μs
ΔT/T
Wavelength (nm)
d
BF2 10 wt% CBP
600 - 640 nm (GSB)
680 - 700 nm (SE)
Normalised ΔT/T
Time (ns)
e
BF2 40 wt% in CBP
1 - 2 ns
5 - 6 ns
10 - 20 ns
100 - 200 ns
1 - 2 μs
5 - 10 μs
20 - 50 μs
ΔT/T
Wavelength (nm)
f
BF2 40 wt% CBP
600 - 650 nm
710 - 750 nm
Normalised ΔT/T
Time (ns)
g
BF2 Neat
1 - 2 ns
5 - 6 ns
10 - 20 ns
50 - 60 ns
100 - 200 ns
1 - 2 μs
ΔT/T
Wavelength (nm)
h
BF2 Neat
630 - 660 nm
Normalised ΔT/T
Time (ns)

Supplementary Figure 11: The nanosecond TA spectra and kinetics of BF2 at 2, 10, and 40 wt% in CBP and a neat BF2 film. (a) The TA spectrum of BF2 at 2 wt% in CBP, excited at 532 nm with a fluence of 692 μJ cm^{-2}. **(b)** The corresponding TA kinetics for the BF2 at 2 wt% in CBP film. **(c)** The TA spectrum of BF2 at 10 wt% in CBP, excited at 532 nm with a fluence of 346 μJ cm^{-2}. **(d)** The corresponding TA kinetics for the BF2 at 10 wt% in CBP film. **(e)** The TA spectrum of BF2 at 40 wt% in CBP, excited at 532 nm with a fluence of 59.7 μJ cm^{-2}. **(f)** The corresponding TA kinetics for the BF2 at 40 wt% in CBP film. **(g)** The TA spectrum of the neat BF2 film, excited at 532 nm with a fluence of 47.6 μJ cm^{-2}. **(h)** The corresponding TA kinetics for the neat BF2 film.

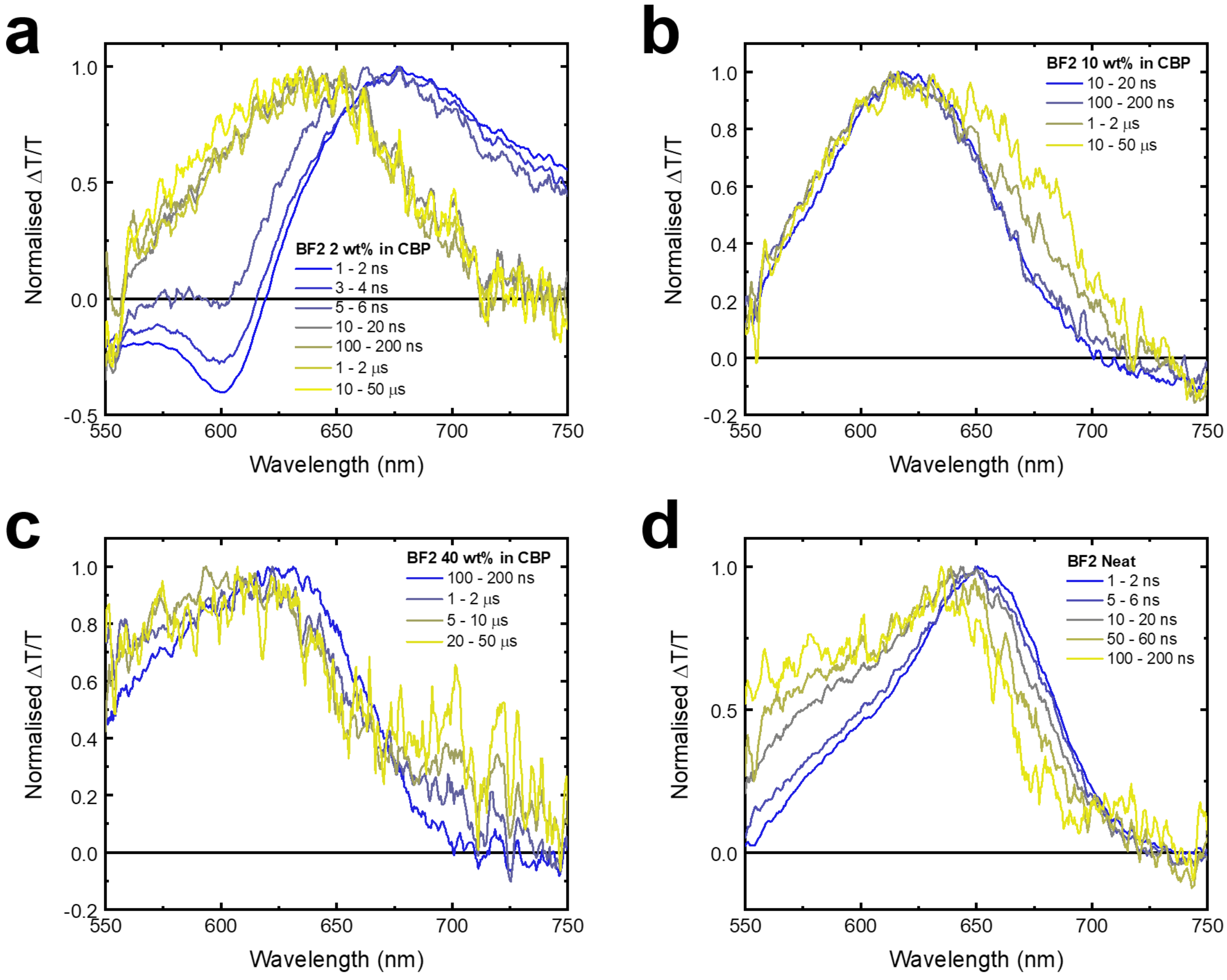

Supplementary Figure 12: The normalised nanosecond TA spectra of BF2 at 2, 10, and 40 wt% in CBP and a neat BF2 film. (a) The normalised TA spectrum of the BF2 at 2 wt% in CBP film. **(b)** The normalised TA spectrum of the BF2 at 10 wt% in CBP film. **(c)** The normalised TA spectrum of the BF2 at 40 wt% in CBP film. **(d)** The normalised TA spectrum of the neat BF2 film. The regrowth of the SE at the lower energy edge of the GSB can clearly be seen in the 10 and 40 wt% films but is absent in the 2 wt% and neat films.

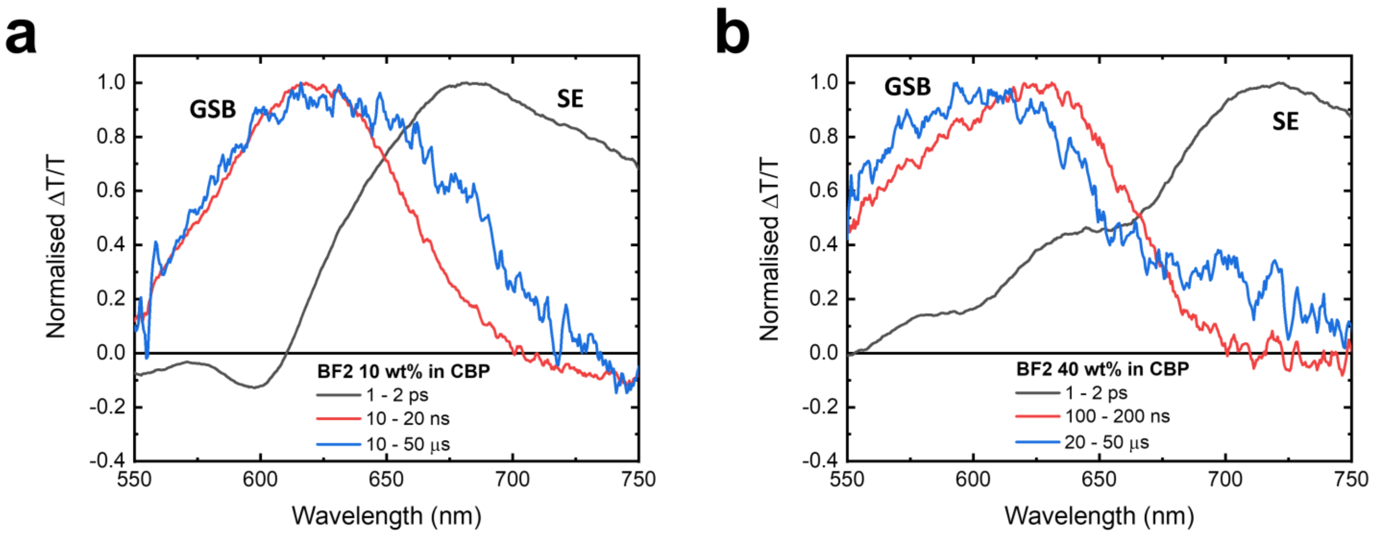

Supplementary Figure 13: A comparison of the ultrafast and nanosecond TA spectra for the 10 and 40 wt% BF2 in CBP films. (a) The TA spectrum of the BF2 at 10 wt% in CBP film, normalised at 3 representative time points. **(b)** The TA spectrum of the BF2 at 40 wt% in CBP film, normalised at 3 representative time points. In both films, the SE feature at 1 – 2 ps overlaps closely with the spectral position of the additional positive sign feature that grows in at the low energy edge of the GSB on microsecond timescales. This confirms that the low energy band represents the regrowth of the SE, induced by the reformation of bright intra-^{1}CT excitons from inter-^{1}CT states.

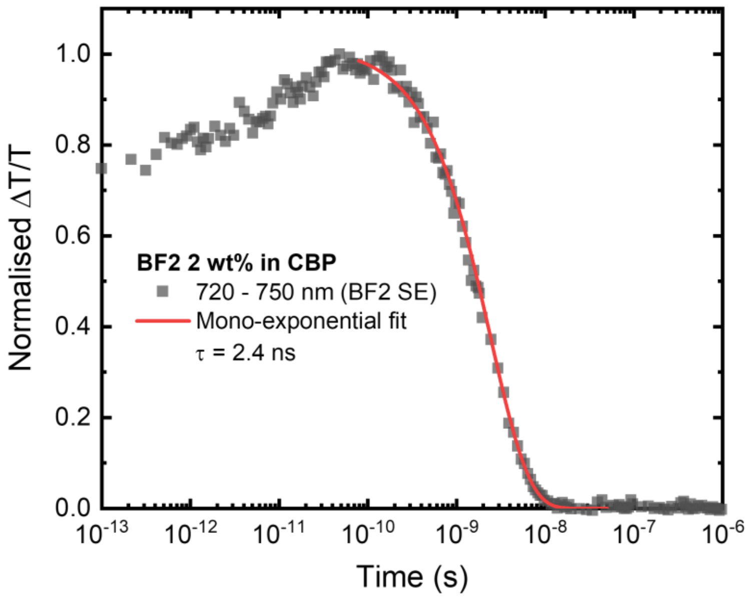

Supplementary Figure 14: A combination of the ultrafast and nanosecond TA kinetics for the SE feature of the 2 wt% BF2 in CBP film. The SE decay can be fitted with a mono-exponential function with a time constant of 2.4 ns, providing a proxy for the radiative lifetime of the BF2 intra-^{1}CT exciton that is not possible to obtain accurately from the trPL measurements due to the limited experimental time resolution of ~5 ns. Such a fast radiative rate is consistent with the extremely strong optical absorption of the BF2 intra-^{1}CT exciton.

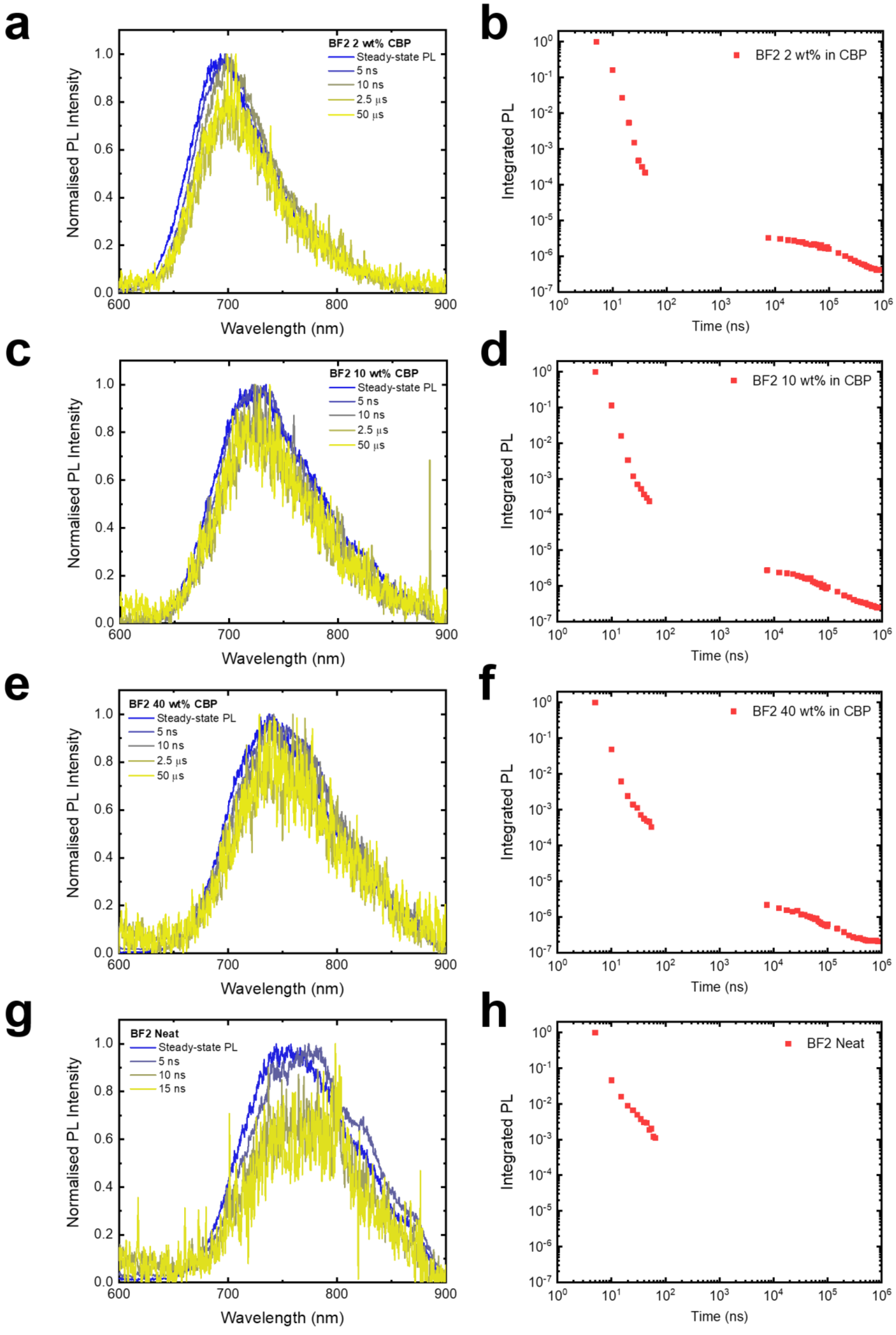
a
BF2 2 wt% CBP
Steady-state PL
5 ns
10 ns
2.5 μs
50 μs
Normalised PL Intensity
Wavelength (nm)
b
BF2 2 wt% in CBP
Integrated PL
Time (ns)
c
BF2 10 wt% CBP
Steady-state PL
5 ns
10 ns
2.5 μs
50 μs
Normalised PL Intensity
Wavelength (nm)
d
BF2 10 wt% in CBP
Integrated PL
Time (ns)
e
BF2 40 wt% CBP
Steady-state PL
5 ns
10 ns
2.5 μs
50 μs
Normalised PL Intensity
Wavelength (nm)
f
BF2 40 wt% in CBP
Integrated PL
Time (ns)
g
BF2 Neat
Steady-state PL
5 ns
10 ns
15 ns
Normalised PL Intensity
Wavelength (nm)
h
BF2 Neat
Integrated PL
Time (ns)

Supplementary Figure 15: The transient PL spectra of BF2 at 2, 10, and 40 wt% in CBP and a neat BF2 film. (a) The trPL spectrum of BF2 at 2 wt% in CBP, excited at 532 nm with a fluence of 27.8 μJ cm^{-2}. **(b)** The corresponding integrated trPL kinetics for the BF2 at 2 wt% in CBP film. **(c)** The trPL spectrum of BF2 at 10 wt% in CBP, excited at 532 nm with a fluence of 27.8 μJ cm^{-2}. **(d)** The corresponding integrated trPL kinetics for the BF2 at 10 wt% in CBP film. **(e)** The trPL spectrum of BF2 at 40 wt% in CBP, excited at 532 nm with a fluence of 27.8 μJ cm^{-2}. **(f)** The corresponding integrated trPL kinetics for the BF2 at 40 wt% in CBP film. **(g)** The trPL spectrum of the neat BF2 film, excited at 532 nm with a fluence of 27.8 μJ cm^{-2}. **(h)** The corresponding integrated trPL kinetics for the neat BF2 film.

BF2 diluted in DPEPO

To determine the effect of the dielectric environment on the ability of BF2 intra-^{1}CT excitons to dissociate into inter-^{1}CT states, we have also investigated BF2 diluted in the polar host DPEPO; DPEPO has a significantly higher dielectric constant of ε =6.1[12], compared to ε =3.5 for CBP[13]. To enable a direct comparison to our studies on BF2 diluted in CBP, we have also fabricated DPEPO films with 2, 10, and 40 wt% BF2. In the ultrafast TA of BF2 in DPEPO (Supplementary Figure 16), we observe similar spectral features to the CBP hosted films; all samples show a broad and positive sign feature between 600 – 750 nm, which we assign to the overlapping GSB and SE of BF2. When comparing the 2, 10, and 40 wt% in DPEPO films, we see that the SE decays more quickly as the BF2 concentration increases (Supplementary Figure 17), indicating the quenching of BF2 intra-^{1}CT excitons by intermolecular charge transfer to form inter-^{1}CT states. In the nanosecond TA of the BF2:DPEPO films (Supplementary Figure 18), we observe a long-lived BF2 GSB feature, which we attribute to the presence of inter-^{1}CT states. Thus, we conclude that the polarity of the host environment has only a limited influence on the ability of BF2 to form well-separated and long-lived inter-^{1}CT states that have the potential to undergo ISC/rISC mediated by the HFI.

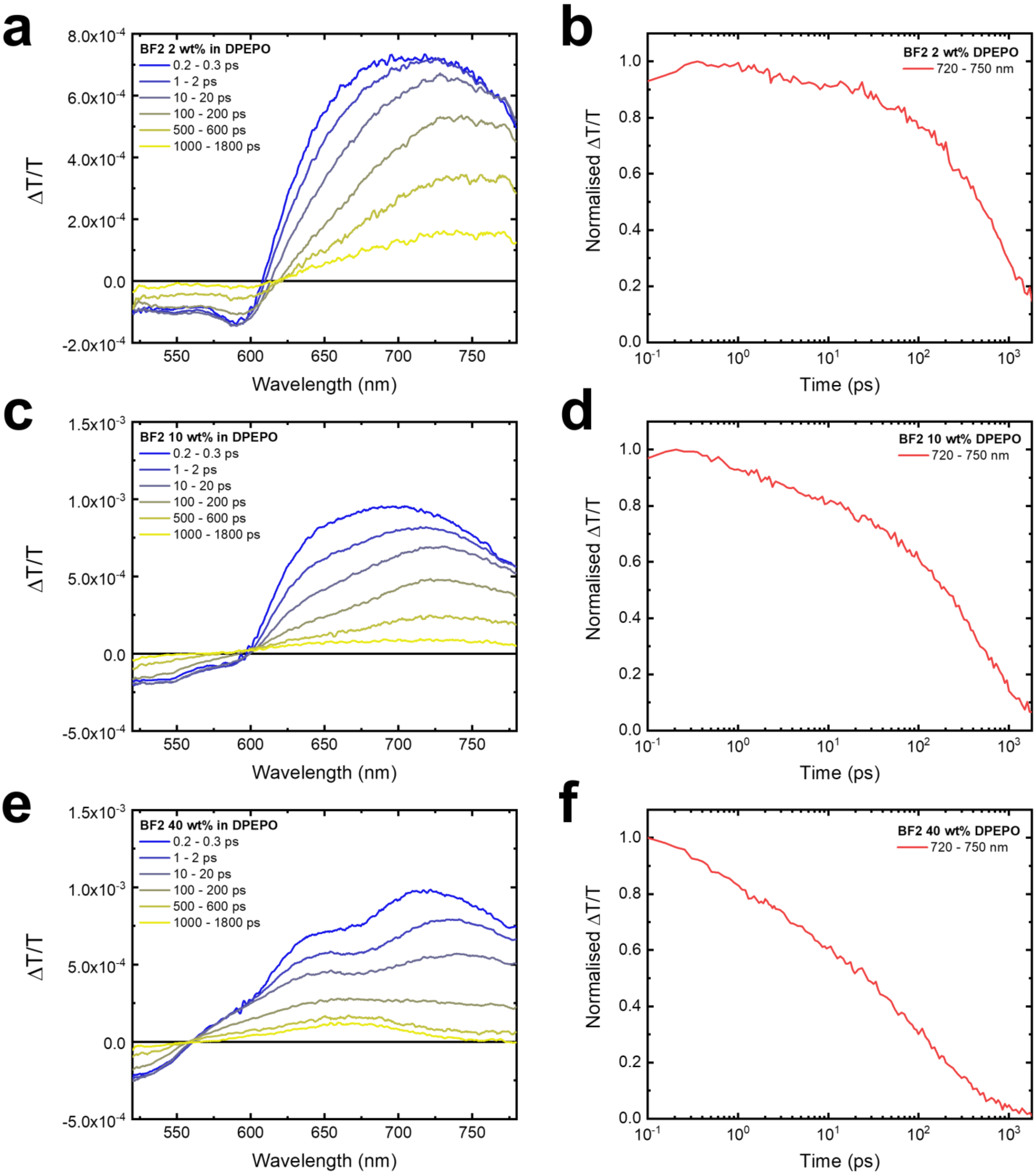
a
BF2 2 wt% in DPEPO
0.2 - 0.3 ps
1 - 2 ps
10 - 20 ps
100 - 200 ps
500 - 600 ps
1000 - 1800 ps
ΔT/T
Wavelength (nm)
b
BF2 2 wt% DPEPO
720 - 750 nm
Normalised ΔT/T
Time (ps)
c
BF2 10 wt% in DPEPO
0.2 - 0.3 ps
1 - 2 ps
10 - 20 ps
100 - 200 ps
500 - 600 ps
1000 - 1800 ps
ΔT/T
Wavelength (nm)
d
BF2 10 wt% DPEPO
720 - 750 nm
Normalised ΔT/T
Time (ps)
e
BF2 40 wt% in DPEPO
0.2 - 0.3 ps
1 - 2 ps
10 - 20 ps
100 - 200 ps
500 - 600 ps
1000 - 1800 ps
ΔT/T
Wavelength (nm)
f
BF2 40 wt% DPEPO
720 - 750 nm
Normalised ΔT/T
Time (ps)

Supplementary Figure 16: The ultrafast TA spectra and kinetics of BF2 at 2, 10 and 40 wt% in DPEPO. The TA spectrum of BF2 at 2 wt% in DPEPO, excited at 600 nm with a fluence of 4.7 μJ cm^{-2}. **(b)** The corresponding TA kinetics for the BF2 at 2 wt% in DPEPO film. **(c)** The TA spectrum of BF2 at 10 wt% in DPEPO, excited at 600 nm with a fluence of 1.7 μJ cm^{-2}. **(d)** The corresponding TA kinetics for the BF2 at 10 wt% in DPEPO film. **(e)** The TA spectrum of BF2 at 40 wt% in DPEPO, excited at 600 nm with a fluence of 2.0 μJ cm^{-2}. **(f)** The corresponding TA kinetics for the BF2 at 40 wt% in DPEPO film.

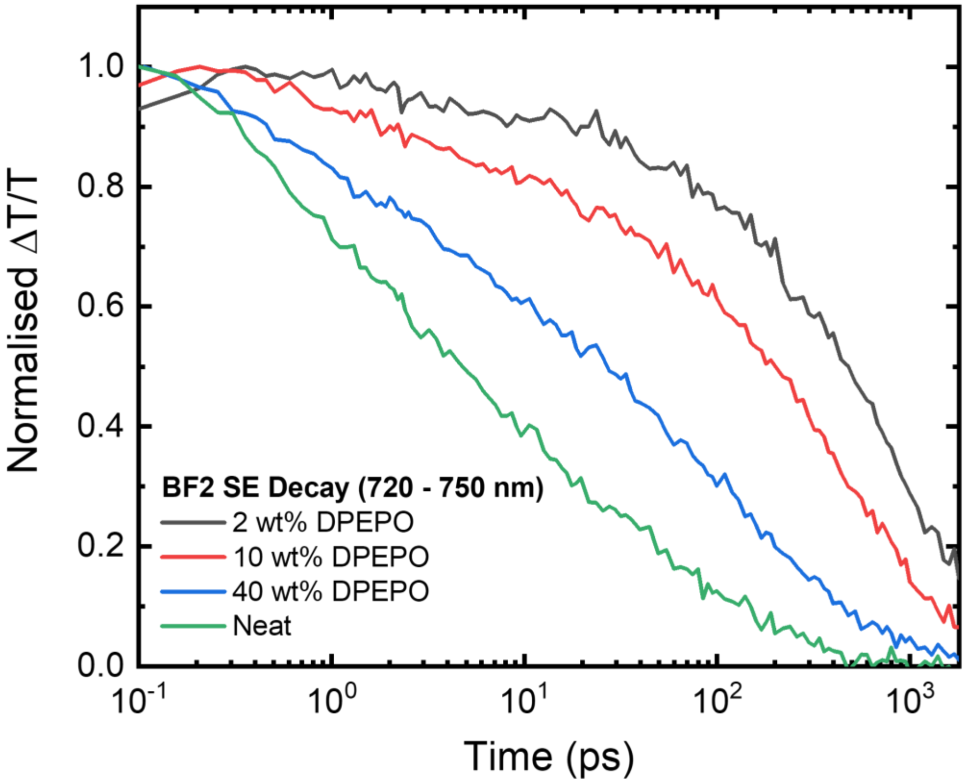

Supplementary Figure 17: The normalised ultrafast TA kinetics of the BF2 SE (720 – 750 nm) for the 2, 10, and 40 wt% films in DPEPO and the neat BF2 film. The lifetime of the SE falls rapidly as the doping concentration is increased, confirming that the increased contact between BF2 molecules results in faster intermolecular charge transfer.

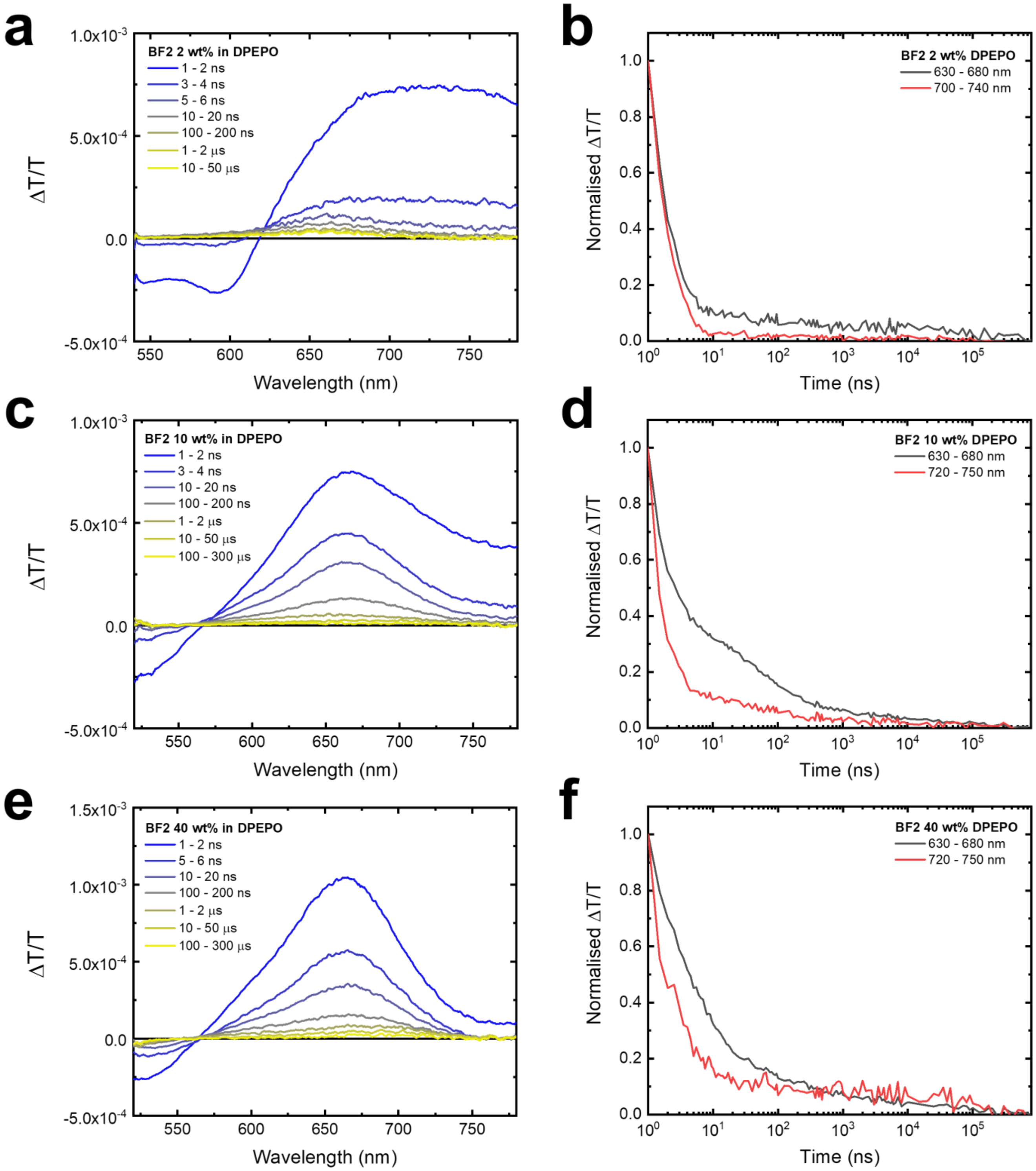

Supplementary Figure 18: The nanosecond TA spectra and kinetics of BF2 at 2, 10, and 40 wt% in DPEPO. (a) The TA spectrum of BF2 at 2 wt% in DPEPO, excited at 532 nm with a fluence of 377 μJ cm^{-2}. **(b)** The corresponding TA kinetics for the BF2 at 2 wt% in DPEPO film. **(c)** The TA spectrum of BF2 at 10 wt% in DPEPO, excited at 532 nm with a fluence of 75.3 μJ cm^{-2}. **(d)** The corresponding TA kinetics for the BF2 at 10 wt% in DPEPO film. **(e)** The TA spectrum of BF2 at 40 wt% in DPEPO, excited at 532 nm with a fluence of 57.2 μJ cm^{-2}. **(f)** The corresponding TA kinetics for the BF2 at 40 wt% in DPEPO film.

PLDMR spectroscopy and hyperfine interactions

In PLDMR spectroscopy, an external magnetic field (**B_0** ~330 mT) energetically splits the T_+ and T_- triplet sublevels from the S and T_0 through the Zeeman effect[14]. By sweeping **B_0**, Zeeman sublevel transitions are induced in the triplet manifold when the applied X-band microwaves are resonant with the transition energy. To enable detection with a high sensitivity, the sample is illuminated with a 405 nm continuous-wave laser, which initially creates singlet Frenkel excitons, and the resulting PL is monitored. When the fixed-frequency microwaves become resonant with a Zeeman transition, the steady-state triplet sublevel populations will be modified and the PL yield will be enhanced or quenched, depending on whether the new triplet sublevel occupancies can better couple to the emissive singlet manifold than the unperturbed population[15]. Thus, PLDMR is well-suited to detecting and identifying triplet states in organic materials.

The HFI originates from the magnetic coupling between the electron spin and the nuclear spin (primarily of ^{1}H and ^{14}N nuclei[16]); as a result, every organic molecule possesses a local hyperfine field (**B_{HF}**) about which the paramagnetic spin of an unpaired electron, known as a radical, will precess[17]. HFI-ISC is typically observed in spin-correlated radical pairs[14,16,18], which consist of two weakly exchange-coupled radicals and can be considered analogous to the loosely-bound inter-CT states present in BF2. Here, local variances in **B_{HF}**, including the different **B_{HF}** experienced by the electron and hole in the inter-CT state[19], results in the spins of individual radicals precessing with slightly different frequencies and, in absence of an applied external magnetic field (**B_0**), also about randomly-distributed axis[14,20]. Thus, the HFI can promote ISC transitions with a rate of ~10^8–10^6 s^{-1} via a periodic oscillation between the

m_s=0 singlet (S) and the m_s=-1,0,+1 triplet sublevels (T_-, T_0, T_+) of the inter-CT state (Figure 3a)[16,21,22]; this is achieved by inducing a phase-lag between the precession of the radical spins[14]. We note similar spin-mixing effects can be achieved through the Δg mechanism, whereby small variations in the g-factor of the positive and negative polarons comprising the inter-CT state can also lead to dephasing between the radical spins[14]. However, the Δg mechanism exhibits a strong dependence on the strength of $\mathbf{B_0}$ and is only able to mediate spin-mixing between the inter-$^1CT_0$ and inter-$^3CT_0$ with a rate of ~10^7-10^6 s^{-1} at fields of ~1 T[14,18,21,23]. Consequently, at the field values studied here (and under OLED device operation at zero-field), it is likely that only the HFI can contribute significantly to the ISC processes between inter-CT states.

To estimate the size of the EPR **D**-parameter (corresponding to the strength of the dipolar interaction between two unpaired spins), and thus the electron-hole separation in the BF2 film, we can use the linewidth of the PLDMR signal. Whilst broadening mechanisms will also contribute to the observed linewidth, an upper estimate of 2**D** can be obtained from the full width at half maximum (FWHM) of the signal. In neat BF2, the FWHM of the inter-CT state signal is ~1.5 mT; this corresponds to **D** ≤0.75 mT. As we now know the strength of the dipolar interaction (in mT), we can estimate an lower bound for the electron-hole separation (in nm) from the following equation[24,25]:

$$r_{e-h} = \sqrt[3]{\frac{2.785}{\boldsymbol{D}}} \tag{1}$$

From this, we obtain a lower bound for the electron-hole separation for the inter-CT state in the neat BF2 film of ≥1.5 nm.

To ascertain the singlet-triplet interconversion timescale enabled by the HFI, we turn to the spin-locking data (Figure 3d). In the PLDMR measurements, the active magnetic field (**B**) is determined by:

$$\boldsymbol{B} = \boldsymbol{B_0} + \boldsymbol{B_{HF}} \tag{2}$$

In the presence of a large $\mathbf{B_0}$, **B** is overwhelmingly determined by $\mathbf{B_0}$; this results in the two unpaired spins precessing around $\mathbf{B_0}$. As previously discussed, this prevents HFI-interaction mediated transitions between S_0 and the T_+ and T_-. However, the spin precession frequency difference resulting from the $\mathbf{B_{HF}}$ component parallel to $\mathbf{B_0}$ ($\Delta\omega_{HF}$) is retained; thus, S_0 to T_0 HFI-induced transitions can still occur. In this case, the singlet-triplet mixing frequency (Ω) is ~$\Delta\omega_{HF}$. However, when $\mathbf{B_1}$ becomes comparable to the $\mathbf{B_{HF}}$ component parallel to $\mathbf{B_0}$ (with $\mathbf{B_1}$ is perpendicular to $\mathbf{B_0}$), the spins reorient and begin to precess around $\mathbf{B_1}$ instead (as viewed in a rotating co-ordinate frame fixed with respect to $\mathbf{B_0}$). As a result, Ω now becomes[26]:

$$\Omega \approx \frac{\Delta\omega_{HF}^2}{2\omega_1} \tag{3}$$

Where ω_1 is the frequency of the spin precession around $\mathbf{B_1}$. From this, we can estimate the effective $\mathbf{B_{HF}}$ ($|\boldsymbol{B_{HF}}|^{eff}$) to be:

$$|\boldsymbol{B_{HF}}|^{eff} \sim 2\boldsymbol{B_1^*} \tag{4}$$

Here, $\boldsymbol{B_1^*}$ represents the microwave field at which the W-shaped resonance starts to develop:

$$\boldsymbol{B}_1^* = \frac{\hbar\omega_{B_1^*}}{g\mu_B} \qquad (5)$$

Where ħ is the reduced Planck's constant, g is the electron g-factor and μ_B is the Bohr magneton. For BF2, $\boldsymbol{B}_1^*$ = 0.38 mT, giving $|\boldsymbol{B}_{HF}|^{eff}$~ 0.76 mT. This can be converted into a frequency (ν) through the following formula:

$$\nu\ (MHz) = 10^{-9}\frac{g\mu_B}{h}B\ (mT) \qquad (6)$$

Where h is Planck's constant. Thus, 0.76 mT corresponds to an effective $\Delta\omega_{HF}$ of 21.3 MHz. As $\Delta\omega_{HF}$ represents the time taken for a complete S_0-T_0-S_0 cycle, the frequency of one singlet to triplet conversion step is 10.7 MHz, equal to an ISC (and rISC) timescale of ~24 ns.

As the HFI-ISC ISC/rISC process is driven by the magnetic coupling between the electron and nuclear spins (Equation 3), it is expected to be temperature independent. However, depending on the energetic alignment of the intra-^{1}CT and inter-^{1}CT states in the system of interest, a temperature dependence would be expected for the intra-^{1}CT dissociation ($E_{intra} < E_{inter}$) or inter-^{1}CT recombination ($E_{intra} > E_{inter}$) processes. For example, as the intra-^{1}CT is slightly higher in energy than the inter-^{1}CT in BF2 (predicted to be 62 meV from our quantum-chemical calculations on the BF2 dimer, Supplementary table 10), we would expect a temperature dependence for the reformation of the intra-^{1}CT after the cycles of ISC/rISC have taken place. Thus, the DF process in aggregated BF2 samples is expected to be temperature dependent. This is consistent with previous observations in a 6 wt% BF2 in CBP film[9], where we expect both the intramolecular rISC (driven by spin-orbit interactions) and intermolecular HFI-ISC processes to be occurring.

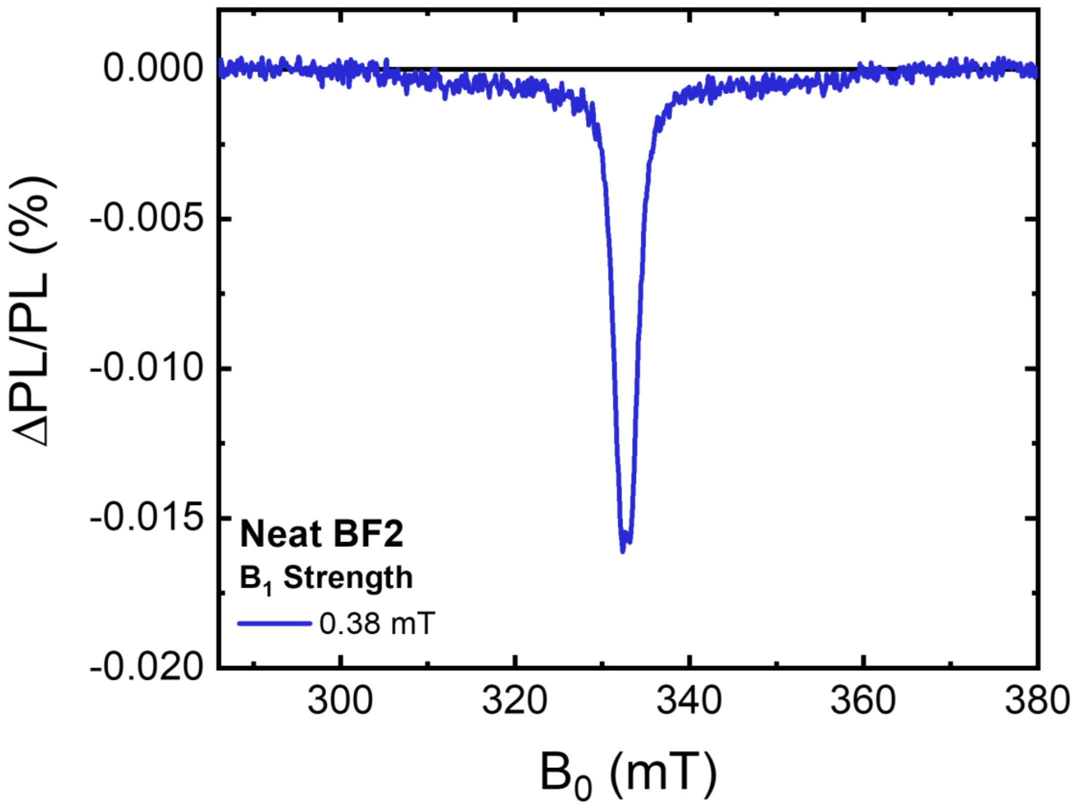

Supplementary Figure 19: The full field range PLDMR spectrum of a neat BF2 film at 293 K with 405 nm excitation (30 mW). The sharp peak at ~333 mT is a signature of inter-CT states, with the weak and broader resonance between 300 – 360 mT originating from triplet excitons localised on a single BF2 molecule.

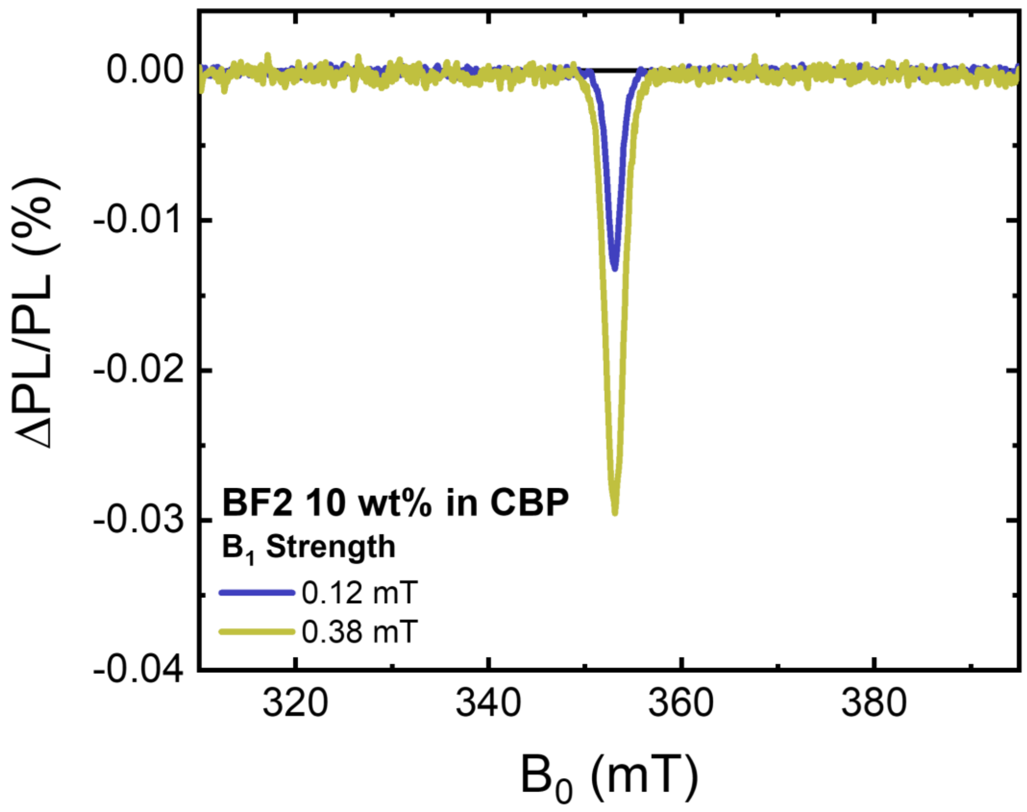

Supplementary Figure 20: The full field range PLDMR spectra of BF2 diluted at 10 wt% in CBP film at 293 K with 405 nm excitation (30 mW). The sharp peak at ~353 mT is a signature of inter-CT states, with no broader resonance observed that could be assigned to triplet excitons localised on a single BF2 molecule. The presence of molecular triplet excitons in the dilute films of the other DF studied here suggests that the formation of these states is suppressed in the BF2 diluted at 10 wt% in CBP film. The inter-CT peak is shifted by ~20 mT compared to the neat film due to a small change in the frequency of the X-band microwaves between measurements. The electron *g*-factor remains unchanged.

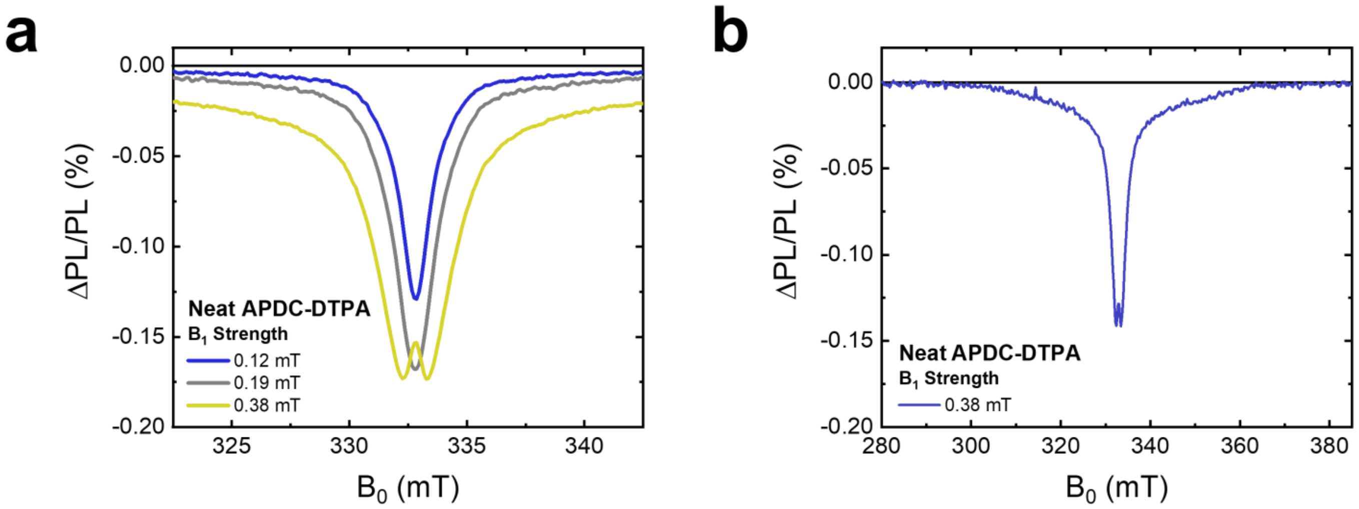

Supplementary Figure 21: The PLDMR of a neat APDC-DTPA film. (a) The PLDMR spectra of a neat APDC-DTPA film at 293 K with 405 nm excitation (30 mW). As $\mathbf{B_1}$ increases, a 'W'-shaped peak begins to form, confirming the spin-locking behaviour and the presence of HFI-ISC processes between inter-CT states in a neat APDC-DTPA film. **(b)** The full field range PLDMR spectrum of the neat APDC-DTPA film. The broader resonance between 300 – 360 mT originates from triplet excitons localised on a single APDC-DTPA molecule.

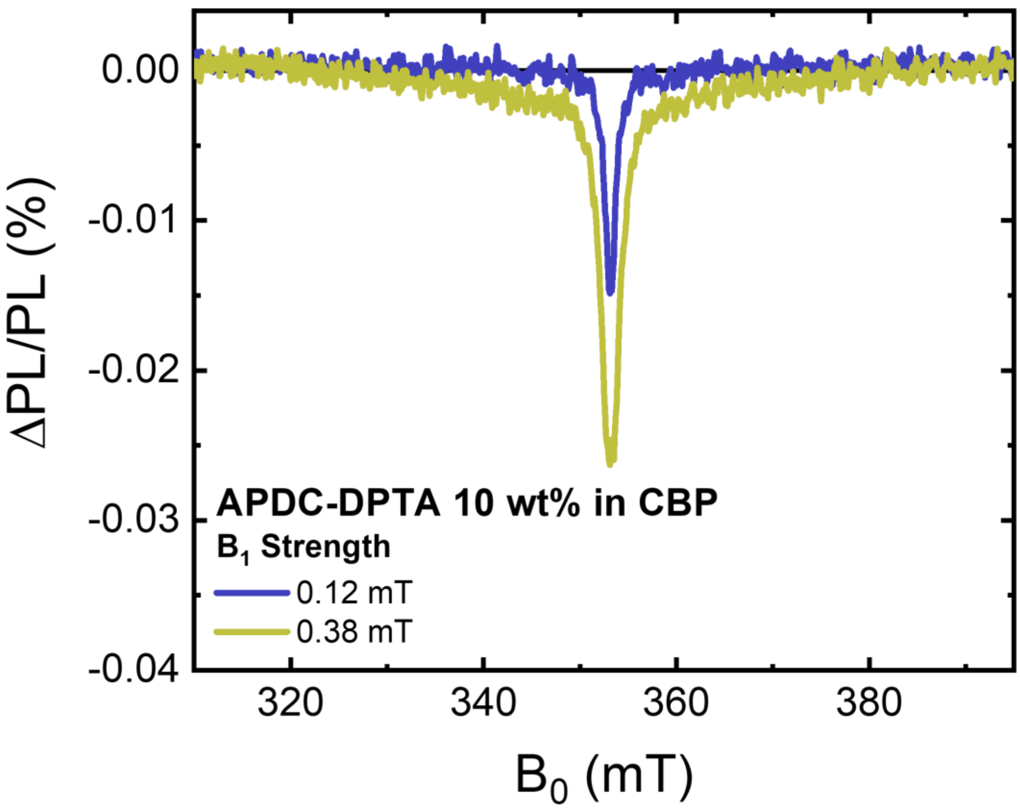

Supplementary Figure 22: The PLDMR spectra of APDC-DTPA diluted at 10 wt % in CBP at 293 K with 405 nm excitation (30 mW). The sharp peak at ~353 mT is a signature of inter-CT states, with the weak and broader resonance between 300 – 360 mT originating from triplet excitons localised on a single APDC-DTPA molecule. The inter-CT peak is shifted by ~20 mT compared to the neat film due to a small change in the frequency of the X-band microwaves between measurements. The electron *g*-factor remains unchanged.

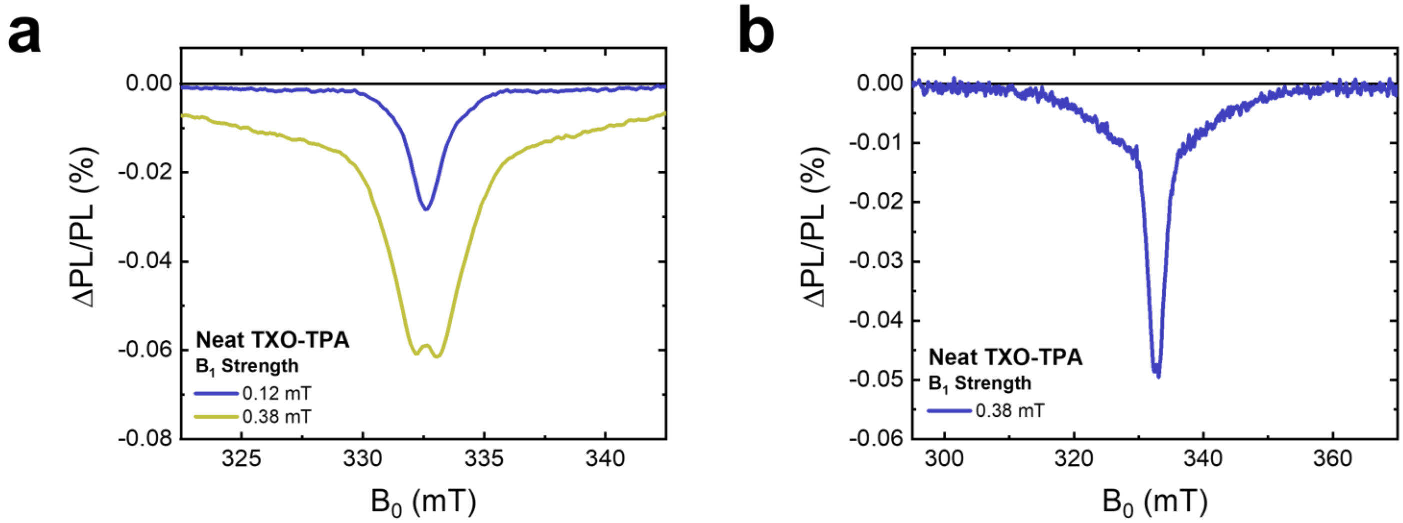

Supplementary Figure 23: The PLDMR of a neat TXO-TPA film. (a) The PLDMR spectra of a neat TXO-TPA film at 293 K with 405 nm excitation (30 mW). As $\mathbf{B_1}$ increases through a reduced microwave attenuation, a 'W'-shaped peak begins to form, confirming the spin-locking behaviour and the presence of HFI-ISC processes between inter-CT states in a neat TXO-TPA film. **(b)** The full field range PLDMR spectrum of the neat TXO-TPA film. The broader resonance between 300 – 360 mT originates from triplet excitons localised on a single TXO-TPA molecule.

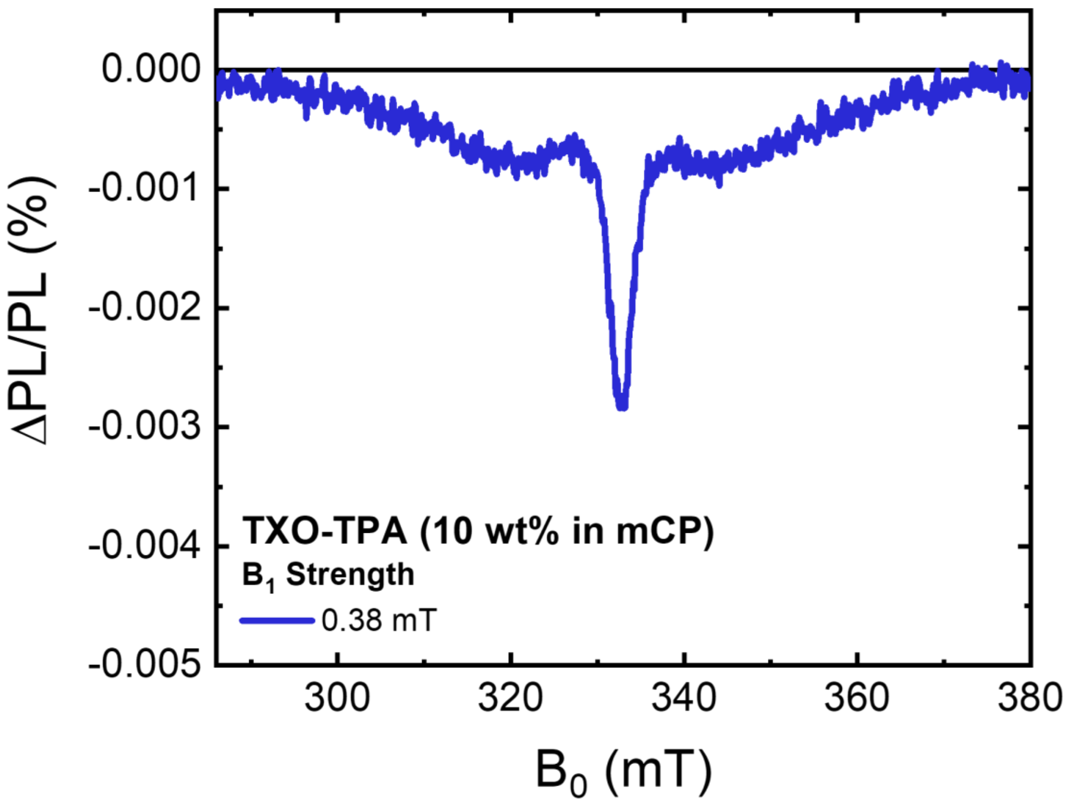

Supplementary Figure 24: The PLDMR spectrum of TXO-TPA diluted in mCP at 10 wt% at 293 K with 405 nm excitation (30 mW). The sharp peak at ~353 mT is a signature of inter-CT states, with the broader resonance between 290 – 370 mT originating from triplet excitons localised on a single TXO-TPA molecule.

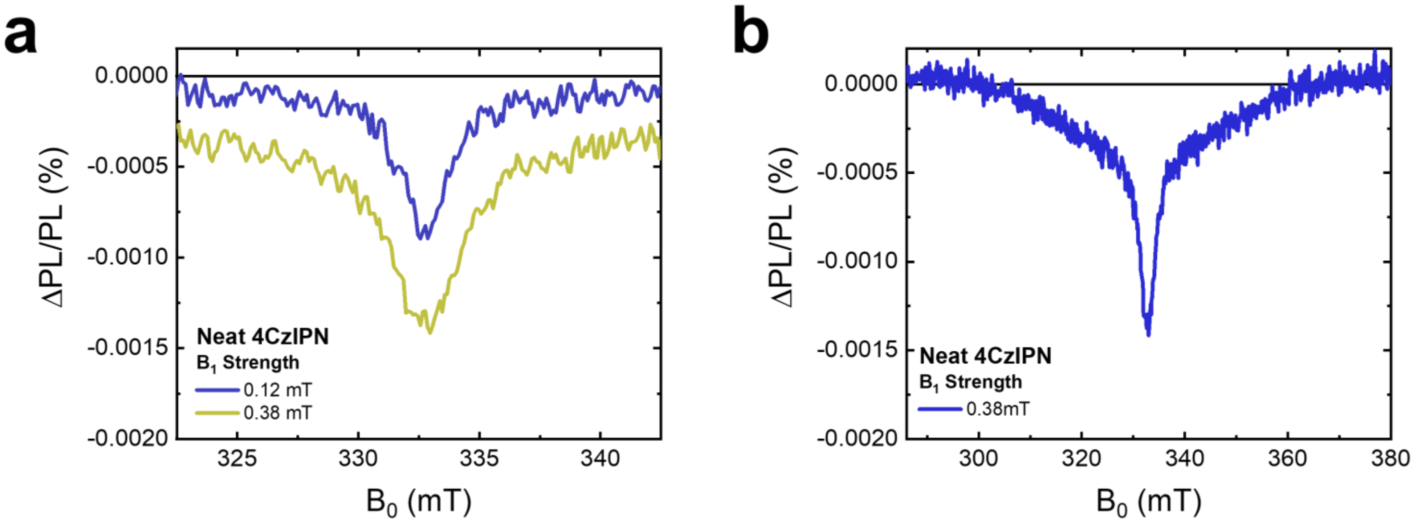

Supplementary Figure 25: The PLDMR of a neat 4CzIPN film. (a) The PLDMR spectra of a neat 4CzIPN film at 293 K with 405 nm excitation (30 mW). The signal is significantly weaker than in the other DF materials included in this study, suggesting a reduced ability of 4CzIPN to form intermolecular CT states where HFI-ISC processes can take place. As $\mathbf{B_1}$ increases, there is no formation of a 'W'-shaped peak, indicating that spin-locking is not observed in the neat 4CzIPN film. **(b)** The full field range PLDMR spectrum of the neat 4CzIPN film. The broader resonance between 300 – 360 mT originates from triplet excitons localised on a single 4CzIPN molecule.

Detailed concentration series of BF2 diluted in CBP

wt% of BF2 in CBP	PLQE in N_2 (%)	PLQE in air (%)
2	44.3 ±4.4	34.1 ±3.4
4	60.3 ±4.5	50.1 ±3.8
6	63.3 ±3.8	51.6 ±3.0
8	51.4 ±2.6	41.2 ±2.1
10	41.1 ±1.6	31.6 ±1.3
20	20.9 ±0.3	15.6 ±0.2
40	10.8 ±0.1	7.4 ±0.07
100 (neat film)	2.6 ±0.01	1.6 ±0.008

Supplementary Table 3: The tabulated PLQE values measured in a nitrogen and air environment for the dilution series of BF2 in CBP, as plotted in Figure 4a. The reported error results from the determination of the PLQE for the weakly absorbing dilute samples with a measured laser power stability of ~1%. Excitation wavelength = 520 nm.

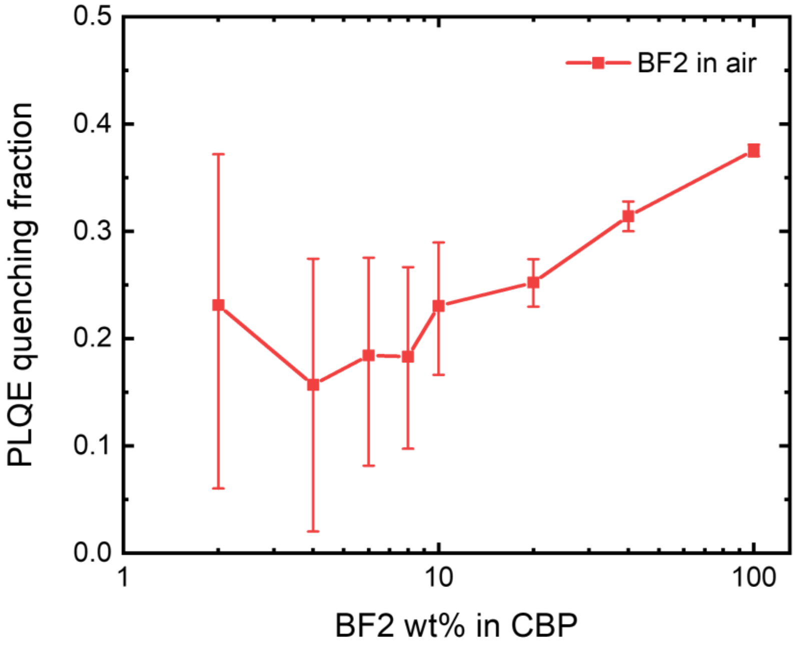

Supplementary Figure 26: The fractional reduction in the PLQE upon exposure to air for the films of BF2 diluted in CBP. The large error bars for the low wt% samples result from the propagated error arising from the determination of the PLQE for weakly absorbing dilute samples with a measured laser power stability of ~1%. However, the general trend is for an increase in the fraction of PLQE quenched by oxygen with increasing BF2 wt%, which is particularly clear for the more concentrated samples where the propagated error is smaller. As only delayed fluorescence, which passes through the triplet manifold, is expected to be quenched by oxygen exposure, this provides evidence for an increased contribution from the delayed fluorescence pathway to the overall emission. Such an observation is consistent with the increased tendency for intra-^{1}CT excitons to dissociate at higher BF2 loadings and undergo ISC/rISC *via* the HFI-ISC mechanism in inter-CT states.

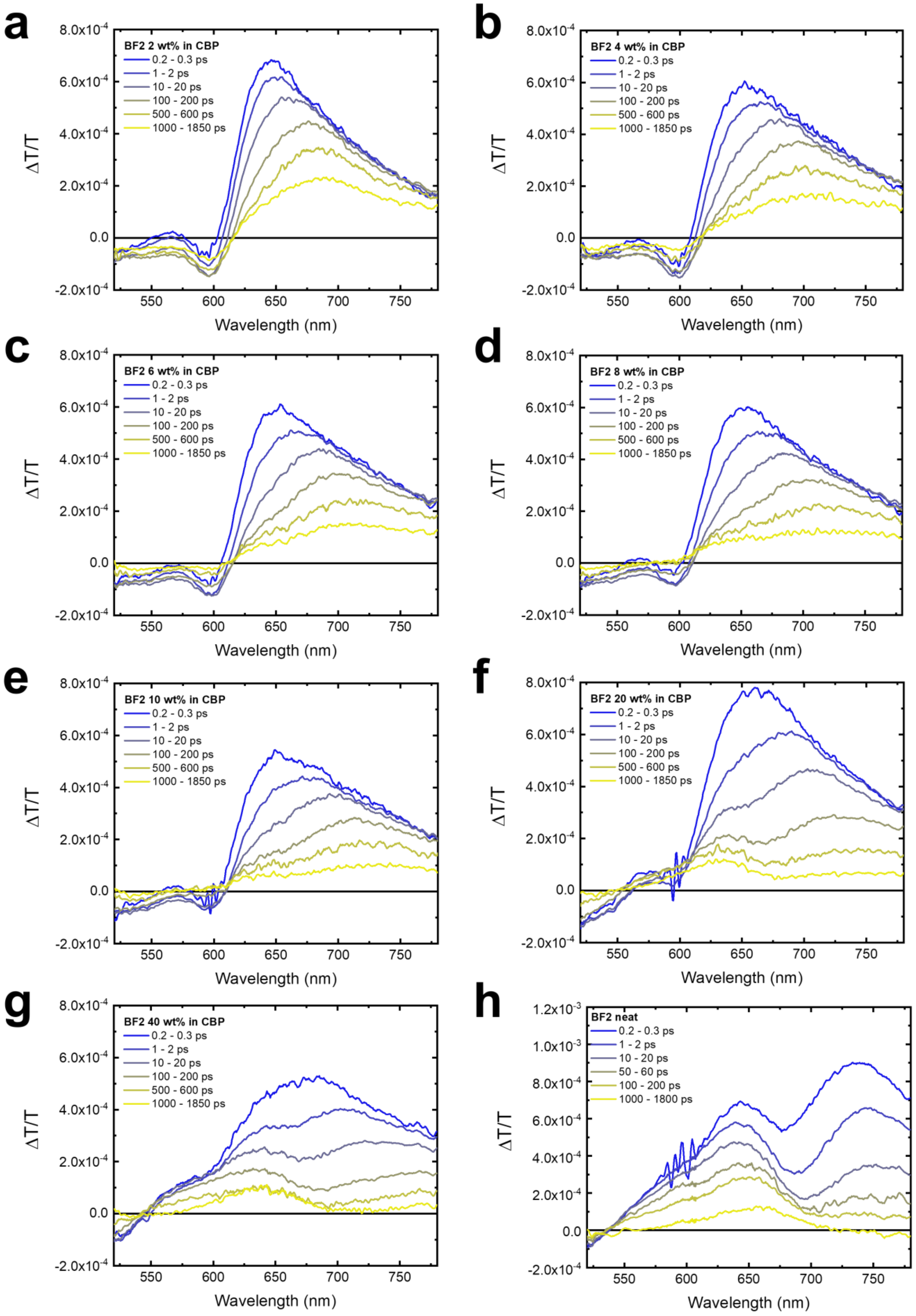
a
BF2 2 wt% in CBP
0.2 - 0.3 ps
1 - 2 ps
10 - 20 ps
100 - 200 ps
500 - 600 ps
1000 - 1850 ps
ΔT/T
Wavelength (nm)
b
BF2 4 wt% in CBP
c
BF2 6 wt% in CBP
d
BF2 8 wt% in CBP
e
BF2 10 wt% in CBP
f
BF2 20 wt% in CBP
g
BF2 40 wt% in CBP
h
BF2 neat
0.2 - 0.3 ps
1 - 2 ps
10 - 20 ps
50 - 60 ps
100 - 200 ps
1000 - 1800 ps

Supplementary Figure 27: The ultrafast TA spectra and kinetics of BF2 at 2, 4, 6, 8, 10, 20, and 40 wt% in CBP and a neat BF2 film. (a) The TA spectra of BF2 at 2 wt% in CBP, excited at 600 nm with a fluence of 2.9 µJ cm^{-2}. **(b)** The TA spectra of BF2 at 4 wt% in CBP, excited at 600 nm with a fluence of 2.3 µJ cm^{-2}. **(c)** The TA spectra of BF2 at 6 wt% in CBP, excited at 600 nm with a fluence of 1.5 µJ cm^{-2}. **(d)** The TA spectra of BF2 at 8 wt% in CBP, excited at 600 nm with a fluence of 1.2 µJ cm^{-2}. **(e)** The TA spectra of BF2 at 10 wt% in CBP, excited at 600 nm with a fluence of 1.1 µJ cm^{-2}. **(f)** The TA spectra of BF2 at 20 wt% in CBP, excited at 600 nm with a fluence of 0.8 µJ cm^{-2}. **(g)** The TA spectra of BF2 at 40 wt% in CBP, excited at 600 nm with a fluence of 0.6 µJ cm^{-2}. **(h)** The TA spectra of the neat BF2 film, excited at 600 nm with a fluence of 1.5 µJ cm^{-2}. These TA measurements were conducted on the same films used for the 'nitrogen' PLQE measurements. The corresponding TA kinetics are plotted in Figure 4b.

Transient absorption of neat 4CzIPN

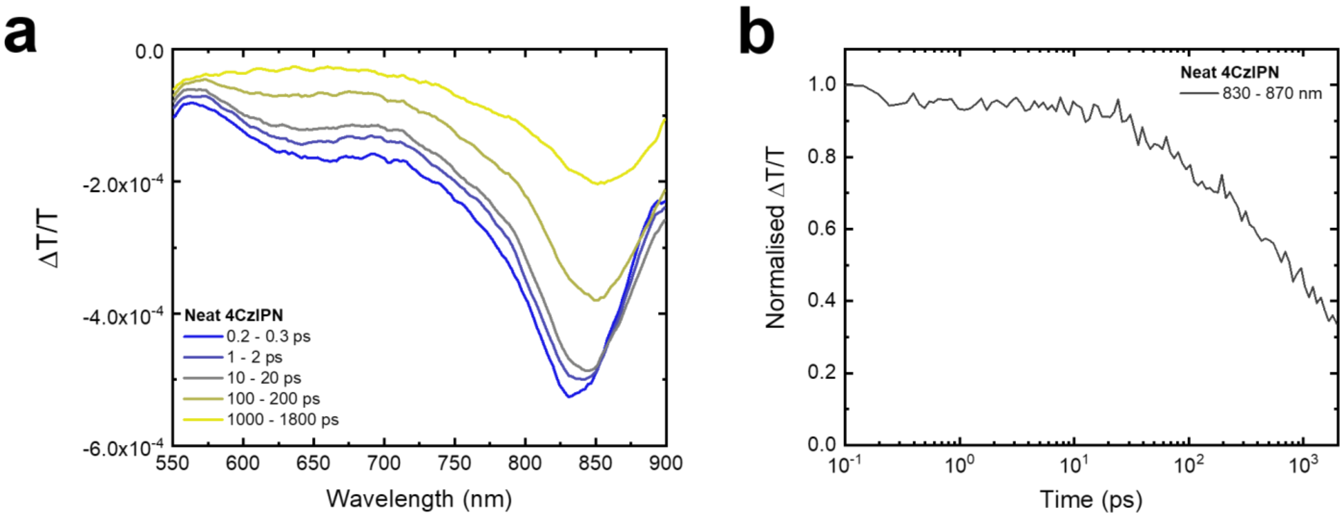

Supplementary Figure 28: The TA of a neat 4CzIPN film. (a) The TA spectra of a neat 4CzIPN film, excited at 450 nm with a fluence of 31.8 µJ cm^{-2}. We observe a small red shift in the 4CzIPN intra-^{1}CT PIA in the first few ps, potentially due to molecular relaxation, but no further spectral evolution on longer timescales. The 4CzIPN intra-^{1}CT PIA at 850 nm begins to decrease in intensity beyond 10 ps as recombination back to the ground state takes place. The absence of significant spectral evolution or the formation of new PIA bands that can be attributed to electron or hole polarons, as observed in a neat BF2 film (Figure 2a), suggests that intermolecular charge transfer does not readily occur in a neat 4CzIPN film on timescales shorter than 2 ns. **(b)** The TA kinetics of the intra-^{1}CT PIA (830 – 870 nm).

Computational details

Screened range separated hybrid approach

Isolated BF2 and 4CzIPN molecules (monomers) were optimised at the DFT level with the exchange-correlation B3LYP[27] functional and the 6-31G(d) basis set for all the atomic species. To model dimers, we took advantage of the crystallographic unit cell of a curcuminoid derivative closely-related to BF2[28] and 4CzIPN[29], reported elsewhere in the literature. In the former, the hydrogen atom of the central dioxaborine ring in the parental curcuminoid molecule was replaced with an ethoxycarbonyl group to match the BF2 molecular structure. Dimer configurations were then optimised at the ground state with DFT, using the same level of theory as above, further including the D3 version of Grimme's empirical dispersion with the Becke-Johnson damping[30]. The DFT optimised BF2 dimer was then used to build a larger aggregate, namely a BF2 tetramer, whose constituting monomers were found to be 3.5 Å apart after the optimisation. Supplementary Figure 21 shows the BF2 and 4CzIPN dimer structures, whose optimisation yielded an intermolecular distance $d_{\perp}$ ~3.5 Å, along with the optimised BF2 tetramer.

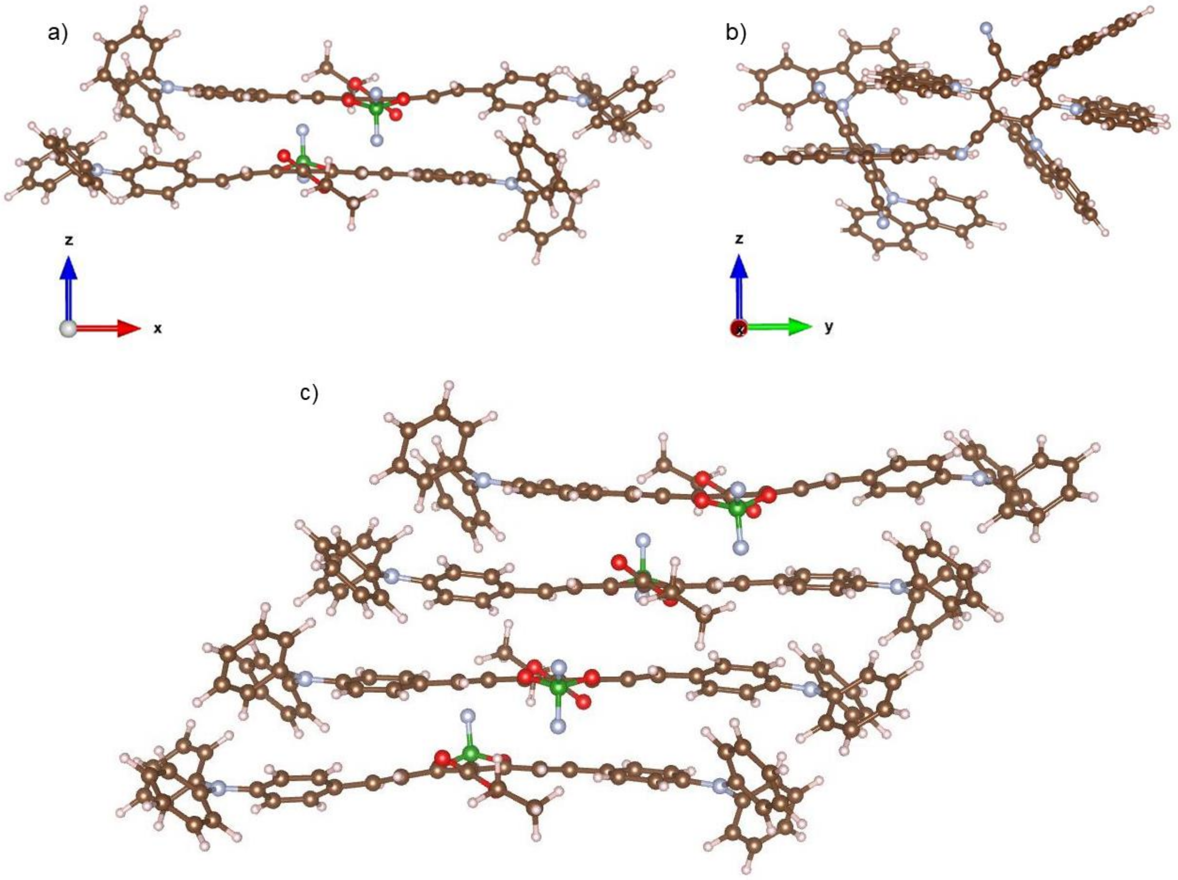

Supplementary Figure 29: Structures of the BF2 and 4CzIPN aggregates. Ground state DFT optimised structure of the a) BF2 dimer, b) 4CzIPN dimer and c) BF2 tetramer.

Optoelectronic properties of the dimers were computed at the Time Dependent (TD) DFT level, resorting to the Tamm-Dancoff approximation (TDA)[31]. Singlet and triplet energies, as well as oscillator strengths, were computed as a function of $d_{\perp}$ by fixing the position of one fragment and shifting the other along the *z* axis. To take into account solid-state screening effects of the surrounding polarizable environment in our calculations, we exploited a *screened* range separated hybrid (SRSH) functional approach[32,33]. In this state-of-the-art method, the Coulomb potential is partitioned into a short-range (SR) and a long-range (LR) contributions, where the asymptotic behaviour of the inter-electron potential tends to $\frac{1}{\varepsilon r}$, so that in the LR domain it is properly screened by the condensed phase scalar dielectric constant ε. In addition, $\frac{1}{\varepsilon} = \alpha + \beta$, where $\alpha + \beta$ quantifies the fraction of exact Hartree-Fock (HF) exchange included in the LR

part, while α quantifies the fraction of HF exchange included in the SR domain. In these calculations, as implemented in Gaussian16[34] and Q-Chem[35] programs, the LC-ωhPBE functional[36] was used along with the 6-311+G(d,p) basis set. Initially, the range-separation parameter ω was optimally tuned in gas-phase for each previously optimized monomer and dimer. The BF2 monomer yielded an ω value of 0.113 $Bohr^{-1}$, while the dimer gave an ω value of 0.086 $Bohr^{-1}$. The 4CzIPN monomer yielded an ω value of 0.107 $Bohr^{-1}$, while the dimer gave an ω value of 0.094 $Bohr^{-1}$. Then, by retaining the α value at 0.2 (typical for organic molecules) and the gas-phase ω value, a dielectric constant of $\varepsilon = 3.00$ was chosen and, thus, the β parameter was set at 0.1333, according to the above equation. As regards the BF2 tetramer, because of computational costs of such a larger aggregate, SRSH TDA TDDFT calculations were carried out for singlets and triplets at the LC-ωhPBE/6-31G(d) level, using the same parameters as for the BF2 dimer (*i.e.*, $\omega = 0.086$ $Bohr^{-1}$, $\alpha = 0.2$ and $\beta = 0.1333$).

Diabatization of low-lying states

Diabatization of the electronic transitions was performed for singlet and triplet excited states computed as described above by means of the Boys localization scheme, as implemented in the Q-Chem program. For monomers and dimers, the 6-311G(d,p) basis set was used, while the tetramer was treated with the 6-31G(d) basis set. The number of adiabatic states considered corresponds to the number of intra- and inter-CT contributions for each system. Namely, diabatization of BF2 and 4CzIPN monomers was obtained from two and four adiabatic states, respectively. For the molecular dimers, eight adiabatic states were considered for BF2 (four intra-CT and four inter-CT) and sixteen for 4CzIPN. For the BF2 tetramer, a total of twenty adiabatic states were considered (eight intra-CT and twelve inter-CT).

Spin-Component Scaling second-order approximate Coupled-Cluster (SCS-CC2) excited states calculations

The isolated BF2 monomer was optimised at the ground state at the DFT-PBE0 level of theory using the def2-TZVP basis set[37]. Subsequently, excited state energies were obtained at the SCS-CC2/def2-TZVP level of theory using the spin-adapted formulation of the linear response theory. All SCS-CC2 calculations were performed with Turbomole/7.4 package[38].

BF2 dimer structure

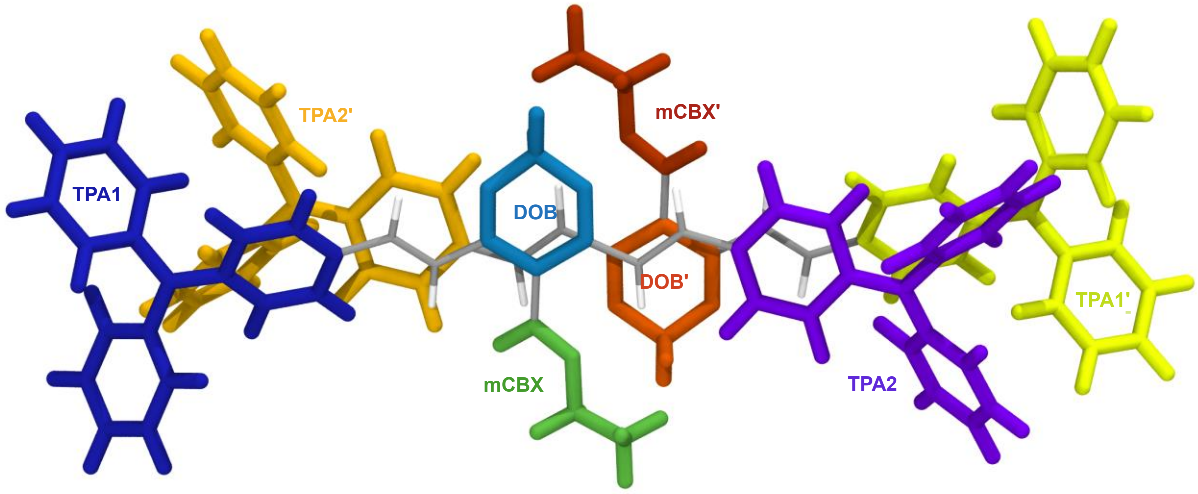

Supplementary Figure 30: Optimised structure of the ground state BF2 dimer. The nomenclature of the fragments considered in the diabatization analysis is included for reference.

BF2 monomer: singlet states

Diabatization of singlet excited states of the BF2 monomer: Z_1 and Z_2 are $D_1 \rightarrow A$ and $D_2 \rightarrow A$ diabatic contributions, respectively. D_1 = TPA1; D_2 = TPA2; A = DOB.

i	S_i	Z_i
1	2.54 (2.22)	2.79
2	2.94 (0.07)	2.83

ω	Z_1	Z_2
S_1	54	46
S_2	46	54

Supplementary Table 4: Electronic states in the BF2 monomer. (left) Energies of adiabatic (S_i) and diabatic (Z_i) states (in eV, oscillator strength in parenthesis); (right) Diabatic contributions ω (in %) as obtained from Boys diabatization for the two lowest singlet states of the BF2 monomer.

State	TPA1	DOB	TPA2	mCBX
Z_1	0.632	-0.333	-0.101	0.003
Z_2	-0.086	-0.326	0.604	0.003

Supplementary table 5: Relative Mulliken fragment charges of diabatic states of the BF2 monomer with respect to the ground state charge distribution.

The two lowest excited states S_1 and S_2 are the in-phase/out-of-phase combinations of two quasi-degenerate zwitterionic states Z_1 and Z_2 that correspond to the intra-CT from each TPA to the central DOB unit.

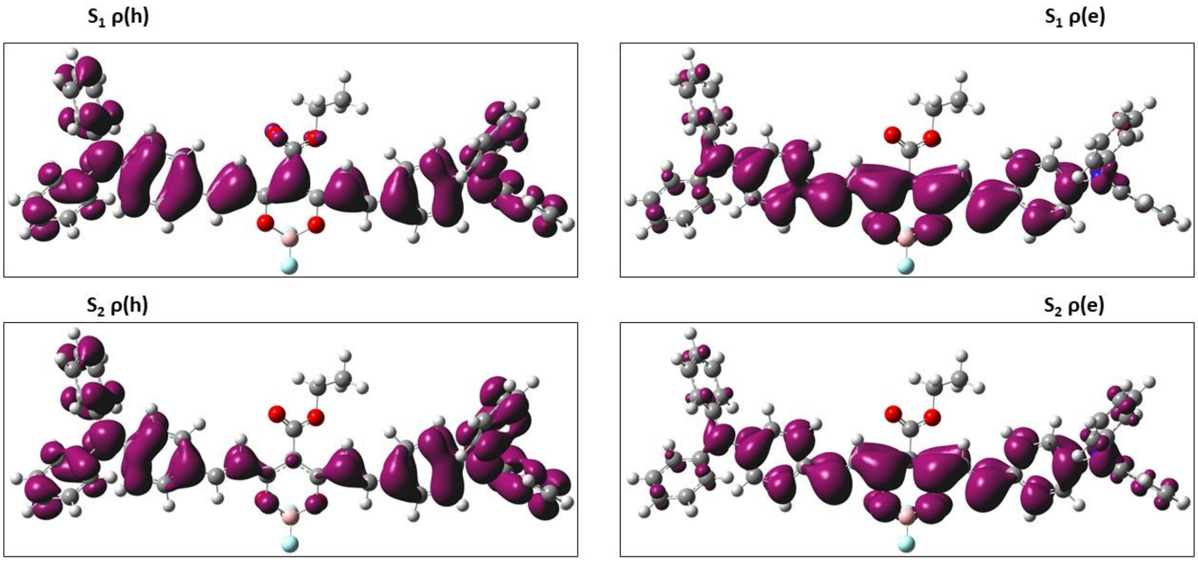

Supplementary Figure 31: Hole (ρ(h)) and electron (ρ(e)) density plots of the two lowest excited states S_1 and S_2 of the BF2 monomer.

BF2 monomer: ΔE_{ST}

	T_1 (eV)	T_2 (eV)	S_1 (eV)	S_2 (eV)
**SRSH TDA TDDFT**	1.83	2.01	2.35	2.72
**SCS-CC2**	2.48	2.63	2.76	3.19

Supplementary Table 6: T_1, T_2, S_1 and S_2 energies computed at the SRSH TDA TDDFT/6-311G+(d,p) and SCS-CC2/def2-TZVP levels of theory.

In addition, to obtain a more robust estimation of the singlet-triplet energy gap (ΔE_{ST}), higher-level of theory SCS-CC2/def2-TZVP calculations were performed on the BF2 monomer at its ground state optimised structure. If, on the one hand, SRSH TDA TDDFT calculations yielded a ΔE_{ST} of 0.52 eV (with S_1 at 2.35 eV and T_1 at 1.83 eV), on the other hand, SCS-CC2 ones gave a lower ΔE_{ST} of 0.28 eV (with S_1 at 2.76 eV and T_1 at 2.48 eV), in close agreement with experimental observations (Supplementary Figure 3).

BF2 dimer, $d_{\perp}$ = 3.5 Å: singlet states

i	S_i	Z_{intra}	Z_{inter}	Z_1	Z_2	Z_3	Z_4	Z_5	Z_6	Z_7	Z_8
1	1.98 (0.00)	41	59	24	23	6	6	10	10	10	10
2	2.07 (0.03)	4	96	45	45	3	3	1	1	1	1
3	2.23 (0.00)	51	49	24	24	0	0	15	15	11	11
4	2.38 (2.04)	66	34	0	0	17	17	5	5	28	28
5	2.44 (2.42)	57	43	0	0	21	21	27	27	2	2
6	2.49 (0.00)	16	84	2	2	40	40	8	8	0	0
7	2.69 (0.03)	73	27	4	4	9	9	17	17	20	19
8	2.73 (0.00)	92	8	0	0	4	4	17	17	29	30

Supplementary Table 7: Energies (in eV) of adiabatic singlet (S_i) states (oscillator strength in parenthesis) and their diabatic composition in terms of intra (in blue) and intermolecular (in red) contributions and Z_i states: $Z_{inter} = Z_1 + Z_2 + Z_3 + Z_4$ and $Z_{intra} = Z_5 + Z_6 + Z_7 + Z_8$.

The four lowest-energy diabatic states correspond to two pairs of degenerate states (Z_1/Z_2 and Z_3/Z_4) with inter-CT character. Symmetry breaking between the two TPA groups of BF2 because of the slip of one monomer with respect to the other along the long molecular axis lifts the degeneracy between $Z_{1/2}$ and $Z_{3/4}$. The nearly degenerate Z_5-Z_8 states on the other hand correspond to intra-CT excitations. The computed adiabatic excited states all exhibit mixing of these inter- and intra-CT diabatic states. Significant inter-CT and strong inter/intra coupling suggests that Kasha's model breaks down here.

state	ΔE	TPA1	DOB	TPA2	mCBX	TPA1'	DOB'	TPA2'	mCBX'
Z_1	2.12	0.001	0.071	0.770	0.004	-0.158	-0.409	-0.092	-0.002
Z_2	2.13	-0.159	-0.409	-0.091	-0.002	0.001	0.071	0.770	0.004
Z_3	2.44	-0.101	-0.406	-0.160	-0.002	0.765	0.072	-0.004	0.004
Z_4	2.45	0.765	0.072	-0.004	0.004	-0.100	-0.406	-0.161	-0.002
Z_5	2.45	-0.128	-0.328	0.654	0.002	0.006	-0.001	-0.018	0.000
Z_6	2.45	0.006	-0.001	-0.018	0.000	-0.126	-0.328	0.652	0.002
Z_7	2.48	-0.003	-0.003	0.005	-0.002	0.644	-0.348	-0.100	0.001
Z_8	2.48	0.643	-0.348	-0.099	0.001	-0.003	-0.003	0.005	-0.002

Supplementary Table 8: Excitation energy (in eV) and relative Mulliken fragment charges of diabatic states of the BF2 dimer with respect to the ground state charge distribution.

Although states S_1 and S_3 involve the same diabatic states with similar weight, they differ by the sign of the amplitude of Z_1 and Z_2 contributions. S_2 shows a quasi-pure inter-CT character.

Bright states S_4 and S_5 are expressed as a combination of inter-CT excitations (from TPA1' to DOB and TPA1 to DOB', Z_3 and Z_4 respectively) with a mixing of two intra-CT on each molecule, namely Z_5/Z_6 and Z_7/Z_8 (mixing of Z_1 and Z_2 of the monomer).

	e-h radius
Z_1	4.589
Z_2	4.579
Z_3	9.776
Z_4	9.755
Z_5	7.230
Z_6	7.200
Z_7	6.800
Z_8	6.774

	e-h radius	Z_{intra}	Z_{inter}
S_1	6.178	41	59
S_2	4.973	4	96
S_3	5.867	51	49
S_4	7.818	66	34
S_5	8.246	57	43
S_6	9.146	16	84
S_7	7.315	73	27
S_8	7.161	92	8

Supplementary Table 9: Electron-hole (e-h) radius (in Å) for the diabatic (left) and adiabatic (right) singlet states. Intra and intermolecular contributions are indicated in blue and red, respectively.

BF2 dimer, $d_{\perp}$ = 3.5 Å: intra-^{1}CT/inter-^{1}CT couplings

In order to stablish the intra-^{1}CT/inter-^{1}CT relationship in the BF2 dimer, we transform the Z_i basis of diabatic states into a new basis of intra and inter diabats by diagonalizing the respective matrix blocks. Supplementary table 9 shows diabatic energies and electronic couplings between the two lowest intra-^{1}CT and inter-^{1}CT states.

	intra-$^1CT_1$	intra-$^1CT_2$	inter-$^1CT_1$	inter-$^1CT_2$
intra-$^1CT_1$	2.151	0	-131	17
intra-$^1CT_2$	0	2.402	1	3
inter-$^1CT_1$	-131	1	2.089	0
inter-$^1CT_2$	17	3	0	2.093

Supplementary Table 10: Diabatic energies (diagonal values in eV) and electronic couplings (off-diagonal values in meV) between the lowest intra-^{1}CT and inter-^{1}CT states.

Gap between the lowest intra-^{1}CT and inter-^{1}CT states: ΔE = 2.151-2.089 = 0.062 eV.

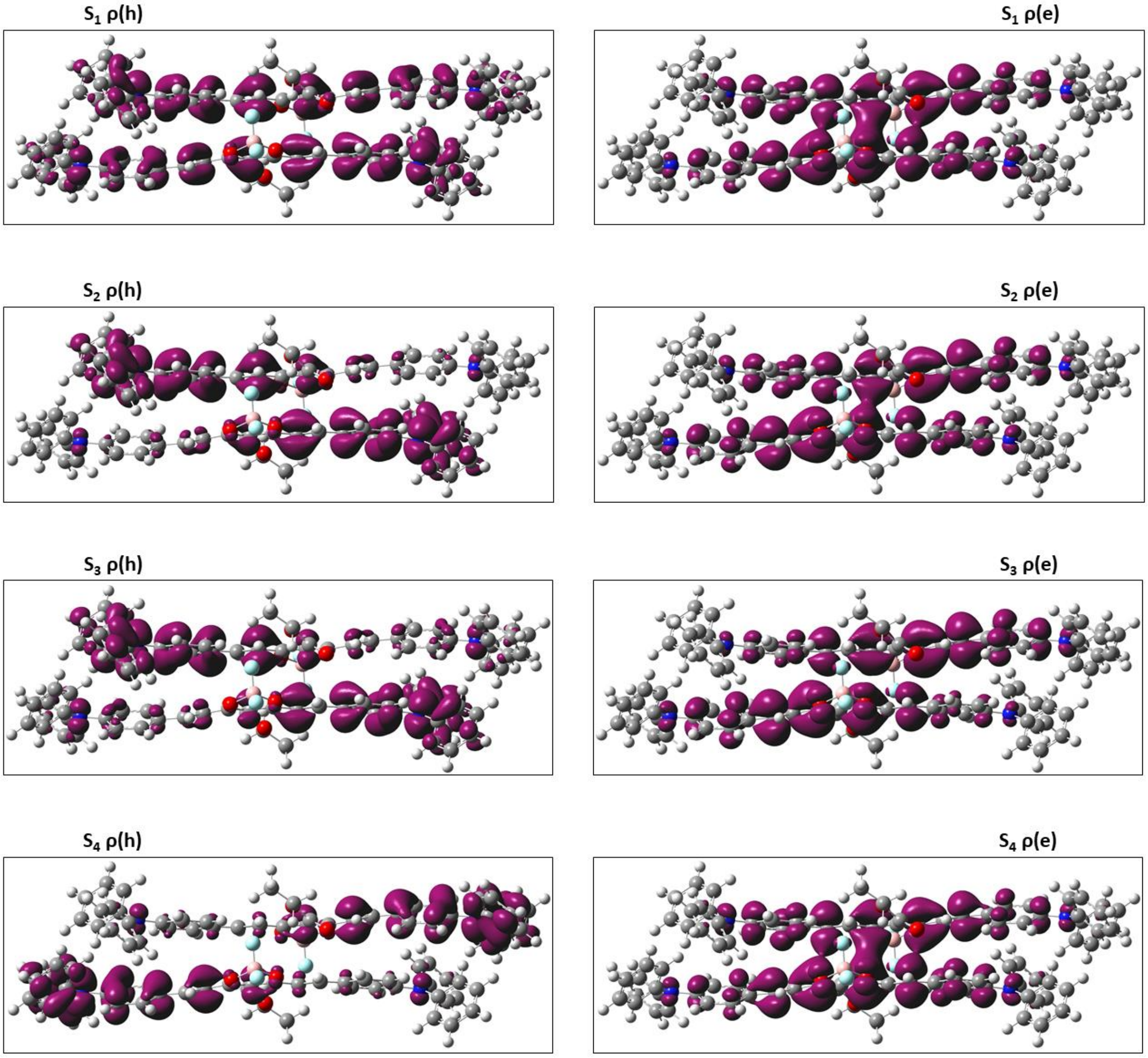

Supplementary Figure 32: Hole (ρ(h)) and electron (ρ(e)) density plots from S_1 to S_4 of the BF2 dimer at $d_{\perp}$ = 3.5 Å.

BF2 dimer, $d_{\perp}$ = 3.5 Å: triplet states

i	T_i	Z_{intra}	Z_{inter}	Z_1	Z_2	Z_3	Z_4	Z_5	Z_6	Z_7	Z_8
1	1.65	81	19	32	31	9	9	7	7	2	2
2	1.75	98	2	39	40	10	10	1	1	0	0
3	1.91	62	38	4	4	27	27	19	19	0	0
4	2.01	98	2	12	12	37	37	0	0	1	1
5	2.12	34	66	6	6	11	11	25	24	9	9
6	2.16	17	83	7	7	2	2	39	41	2	2
7	2.45	4	96	0	0	2	2	3	3	46	44
8	2.50	5	95	0	0	2	2	6	6	40	42

Supplementary Table 11: Energies (in eV) of adiabatic triplet (T_i) states and their diabatic composition in terms of intra (in blue) and intermolecular (in red) contributions and Z_i states: $Z_{intra} = Z_1 + Z_2 + Z_3 + Z_4$ and $Z_{inter} = Z_5 + Z_6 + Z_7 + Z_8$.

State	TPA1	DOB	TPA2	mCBX	TPA1'	DOB'	TPA2'	mCBX'
Z_1	0.006	0.005	-0.006	-0.001	-0.052	-0.258	0.392	0.005
Z_2	-0.052	-0.259	0.395	0.005	0.006	0.005	-0.006	-0.001
Z_3	-0.002	-0.003	-0.002	-0.001	0.386	-0.228	-0.039	0.000
Z_4	0.386	-0.228	-0.039	0.000	-0.002	-0.003	-0.002	-0.001
Z_5	0.001	0.070	0.753	0.003	-0.155	-0.403	-0.089	-0.002
Z_6	-0.155	-0.402	-0.089	-0.002	0.001	0.070	0.753	0.003
Z_7	-0.086	-0.378	-0.126	-0.002	0.701	0.062	-0.018	0.005
Z_8	0.700	0.062	-0.017	0.005	-0.085	-0.378	-0.126	-0.002

Supplementary Table 12: Relative Mulliken fragment charges of diabatic states of the BF2 dimer with respect to the ground state charge distribution.

	e-h radius
Z_1	3.763
Z_2	3.786
Z_3	3.537
Z_4	3.527
Z_5	4.479
Z_6	4.466
Z_7	9.108
Z_8	9.085

	e-h radius	Z_{intra}	Z_{inter}
T_1	4.085	81	19
T_2	3.764	98	2
T_3	3.913	62	38
T_4	3.701	98	2
T_5	4.991	34	66
T_6	4.494	17	83
T_7	8.596	4	96
T_8	8.208	5	95

Supplementary Table 13: Electron-hole (e-h) radius (in Å) for the diabatic (left) and adiabatic (right) triplet states. Intra and intermolecular contributions are indicated in blue and red, respectively.

BF2 dimer, $d_{\perp}$ = 3.5 Å: intra-^{3}CT/inter-^{3}CT couplings

To establish the intra-^{3}CT/inter-^{3}CT relationship in the BF2 dimer, we transform the Z_i basis of diabatic states into a new basis of intra and inter diabats by diagonalizing the respective matrix blocks. Supplementary table 13 shows diabatic energies and electronic couplings between the two lowest intra-^{3}CT and inter-^{3}CT states.

	intra-$^3CT_1$	intra-$^3CT_2$	inter-$^3CT_1$	inter-$^3CT_2$
intra-$^3CT_1$	1.751	0	0	194
intra-$^3CT_2$	0	1.755	32	24
inter-$^3CT_1$	0	32	2.040	0
inter-$^3CT_2$	194	24	0	2.070

Supplementary Table 14: Diabatic energies (diagonal values in eV) and electronic couplings (off-diagonal values in meV) between the lowest intra-^{3}CT and inter-^{3}CT states.

Gap between the lowest intra-^{3}CT and inter-^{3}CT states: ΔE = 2.040-1.751 = 0.289 eV

BF2 dimer, distance dependence

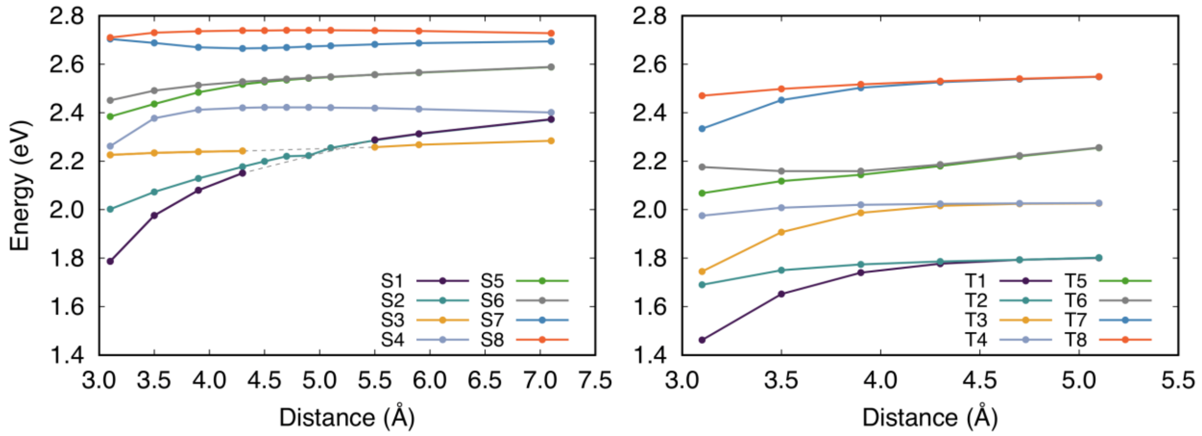

Supplementary Figure 33: Adiabatic singlet (left) and triplet (right) state energies as a function of the distance between the BF2 molecules.

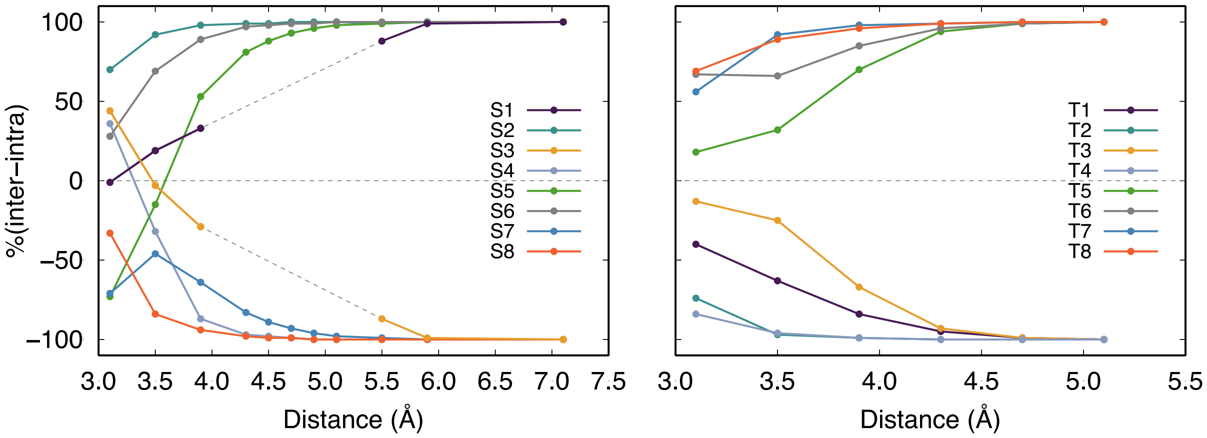

Supplementary Figure 34 Adiabatic singlet (left) and triplet (right) state wave function as a function of the distance between the BF2 molecules.

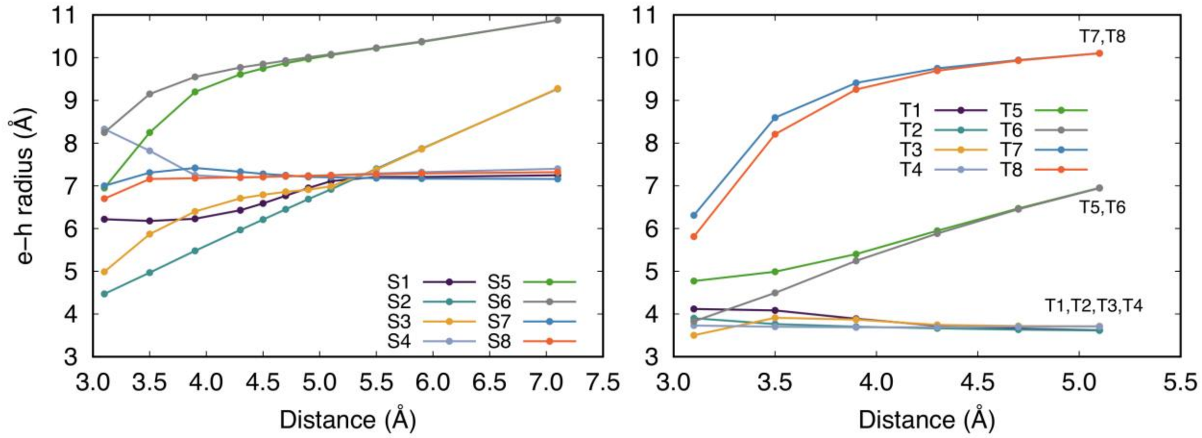

Supplementary Figure 35: Adiabatic singlet (left) and triplet (right) electron-hole (e-h) radius (in Å) as a function of the distance between the BF2 molecules.

BF2 tetramer structure

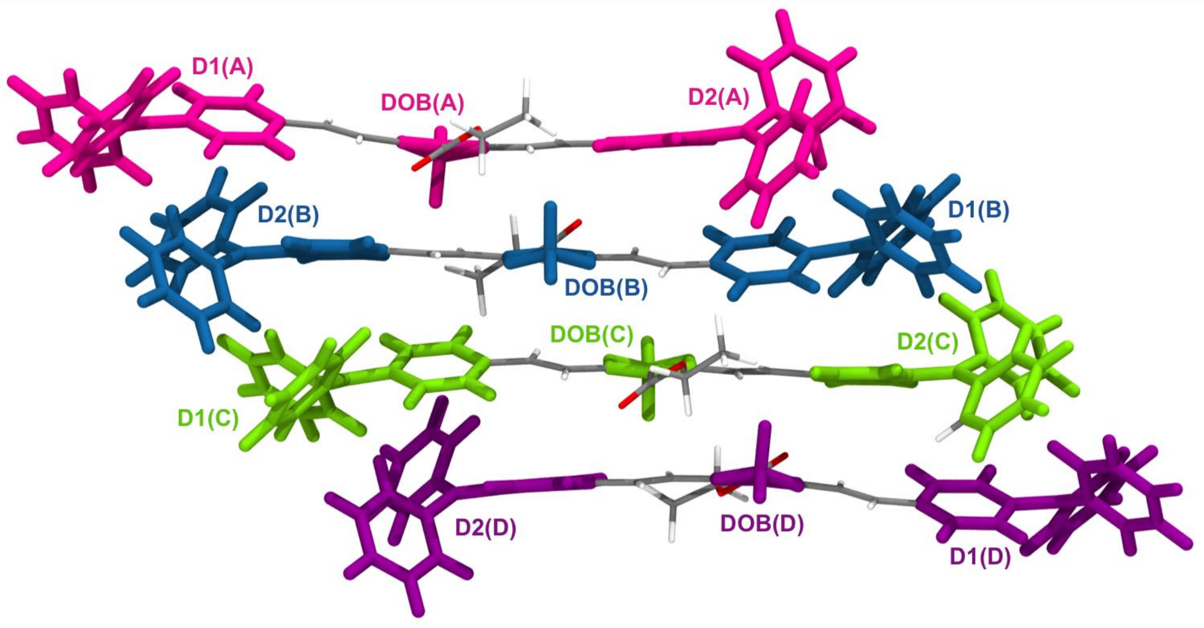

Supplementary Figure 36: Optimised structure of the ground state BF2 tetramer and nomenclature of the fragments considered in the diabatization analysis (D1=TPA1, D2=TPA2).

BF2 tetramer: singlet states

i	S_i	Z_{intra}	Z_{inter}
1	1.87 (0.00)	17	83
2	1.99 (0.02)	8	92
3	2.03 (0.01)	5	95
4	2.05 (0.00)	2	98
5	2.13 (0.04)	5	95
6	2.13 (0.09)	6	94
7	2.19 (0.00)	36	64
8	2.20 (0.03)	53	47
9	2.28 (0.00)	58	42
10	2.29 (0.00)	1	99
11	2.33 (0.02)	7	93
12	2.35 (0.00)	24	76
13	2.36 (0.03)	9	91
14	2.36 (0.13)	8	92
15	2.39 (0.00)	19	81
16	2.44 (0.71)	20	80
17	2.47 (0.01)	13	87
18	2.49 (0.80)	12	88
19	2.51 (0.17)	19	81
20	2.53 (4.68)	77	23

Supplementary Table 15: Energies (in eV) of adiabatic singlet (S_i) states (oscillator strength in parenthesis) and their diabatic composition in terms of intra (in blue) and intermolecular (in red) contributions and Z_i states: $Z_{inter} = Z_{1-8} + Z_{11-16} + Z_{19-20}$ and $Z_{intra} = Z_9 + Z_{10} + Z_{17} + Z_{18}$.

	e-h radius
Z_1	4.242
Z_2	4.123
Z_3	4.082
Z_4	4.630
Z_5	4.271
Z_6	4.651
Z_7	9.891
Z_8	9.827
Z_9	1.307
Z_{10}	1.424
Z_{11}	6.922
Z_{12}	6.917
Z_{13}	6.979
Z_{14}	6.979
Z_{15}	9.011
Z_{16}	8.974
Z_{17}	1.243
Z_{18}	1.632
Z_{19}	10.970
Z_{20}	10.927

	e-h radius	Z_{intra}	Z_{inter}
S_1	5.309	17	83
S_2	5.774	8	92
S_3	4.909	5	95
S_4	5.565	2	98
S_5	4.739	5	95
S_6	4.690	6	94
S_7	5.288	36	64
S_8	3.783	53	47
S_9	3.469	58	42
S_{10}	7.360	1	99
S_{11}	8.680	7	93
S_{12}	5.398	24	76
S_{13}	7.348	9	91
S_{14}	6.767	8	92
S_{15}	7.661	19	81
S_{16}	6.971	20	80
S_{17}	7.441	13	87
S_{18}	7.906	12	88
S_{19}	7.004	19	81
S_{20}	2.937	77	23

Supplementary Table 16: Electron-hole (e-h) radius (in Å) for the diabatic (left) and (right) adiabatic singlet states. Intra and intermolecular contributions are indicated in blue and red, respectively.

state	monomer	ΔE	DOB	TPA1	TPA2	mCBX
Z_9	A	2.28	-0.282	0.129	0.279	0.006
Z_{10}	D	2.28	-0.300	0.142	0.293	0.007
Z_{17}	B	2.36	-0.278	0.264	0.137	0.007
Z_{18}	C	2.37	-0.259	0.246	0.125	0.006

Supplementary Table 17: Intra-CT diabatic states excitation energies (in eV) and relative Mulliken fragment charges of diabatic states of the BF2 tetramer with respect to the ground state charge distribution.

state	monomer	ΔE	DOB	TPA1	TPA2	mCBX
Z_1	C	2.14	0.057	0.001	0.647	0.005
	D		-0.379	-0.105	-0.093	-0.001
Z_2	A	2.14	0.064	0.029	0.651	0.006
	B		-0.386	-0.106	-0.083	0.000
Z_3	D	2.14	0.065	0.032	0.663	0.006
	C		-0.396	-0.116	-0.082	0.000
Z_4	B	2.14	0.037	0.666	0.004	0.004
	C		-0.374	-0.094	-0.087	-0.001
Z_5	B	2.14	0.057	0.001	0.657	0.004
	A		-0.383	-0.106	-0.094	-0.001
Z_6	C	2.15	0.042	0.674	0.006	0.004
	B		-0.378	-0.094	-0.091	-0.001
Z_7	C	2.26	0.030	0.458	-0.025	0.004
	D		-0.357	-0.086	-0.146	-0.001
Z_8	B	2.27	0.038	0.478	-0.023	0.003
	A		-0.365	-0.088	-0.149	-0.001
Z_{11}	B	2.29	0.057	0.694	0.066	0.007
	D		-0.412	-0.140	-0.105	-0.002
Z_{12}	C	2.30	0.057	0.696	0.065	0.007
	A		-0.413	-0.139	-0.106	-0.002
Z_{13}	A	2.31	0.064	0.034	0.747	0.006
	C		-0.418	-0.121	-0.119	-0.002
Z_{14}	D	2.32	0.064	0.034	0.745	0.006
	B		-0.418	-0.121	-0.119	-0.002
Z_{15}	B	2.35	0.017	-0.028	0.520	0.007
	C		-0.258	0.008	-0.056	-0.001
Z_{16}	C	2.35	0.016	-0.029	0.538	0.007
	B		-0.264	-0.009	-0.057	-0.001
Z_{19}	A	2.39	0.061	0.045	0.745	0.006
	D		-0.420	-0.135	-0.122	-0.002
Z_{20}	D	2.40	0.060	0.044	0.744	0.006
	A		-0.420	-0.134	-0.123	-0.002

Supplementary Table 18: Inter-CT diabatic states excitation energies (in eV) and relative Mulliken fragment charges of diabatic states of the BF2 tetramer with respect to the ground state charge distribution.

ω	Z_1	Z_2	Z_3	Z_4	Z_5	Z_6	Z_7	Z_8	Z_9	Z_{10}	Z_{11}	Z_{12}	Z_{13}	Z_{14}	Z_{15}	Z_{16}	Z_{17}	Z_{18}	Z_{19}	Z_{20}
S_1	2	6	5	20	2	18	6	6	0	0	0	0	3	3	5	6	8	8	0	0
S_2	27	1	1	2	23	2	12	11	4	4	5	5	0	0	0	0	0	0	1	1
S_3	0	22	23	14	0	14	0	0	2	2	1	1	9	9	1	1	0	0	1	1
S_4	29	2	3	2	30	1	5	7	0	0	6	6	2	2	2	2	1	1	1	1
S_5	2	49	6	22	1	0	0	0	5	0	0	0	1	4	8	1	0	0	0	0
S_6	2	0	41	4	5	28	0	1	0	4	0	0	3	0	0	9	1	1	0	0
S_7	0	0	0	4	0	4	3	3	1	3	12	11	8	9	0	0	16	16	5	4
S_8	3	3	3	1	3	1	1	2	20	22	4	6	8	5	3	3	7	4	0	0
S_9	9	3	2	0	7	0	3	2	30	27	8	4	0	0	1	1	0	1	3	1
S_{10}	8	0	0	7	9	6	3	4	0	1	14	16	1	1	2	2	0	0	13	12
S_{11}	0	0	0	0	1	0	24	26	4	2	8	5	0	0	10	11	0	0	4	3
S_{12}	9	2	1	1	6	3	8	3	12	10	11	13	11	6	1	0	1	1	0	1
S_{13}	0	7	1	5	3	2	6	10	1	6	11	0	21	1	0	1	2	0	19	4
S_{14}	4	1	8	0	3	3	3	0	6	1	3	16	4	30	1	0	0	0	1	16
S_{15}	2	0	0	4	1	4	15	15	0	1	1	2	0	0	9	6	10	8	10	11
S_{16}	3	1	1	4	3	3	3	6	7	10	0	0	0	0	30	25	0	2	1	0
S_{17}	0	0	0	8	0	7	4	3	3	1	0	0	3	1	21	31	6	3	6	2
S_{18}	0	3	0	1	0	1	1	0	1	3	10	4	16	7	1	0	0	8	27	16
S_{19}	0	2	3	1	0	2	0	1	1	0	4	11	7	15	2	1	16	2	8	23
S_{20}	1	0	1	0	1	0	2	2	1	3	0	0	2	5	1	1	31	43	1	3

Supplementary Table 19: Diabatic contributions ω (in %) as obtained from Boys diabatization. Intra and intermolecular contributions are indicated in blue and red, respectively.

BF2 tetramer: triplet states

i	T_i	Z_{intra}	Z_{inter}
1	1.61	79	21
2	1.68	83	17
3	1.73	94	6
4	1.78	98	2
5	1.86	54	46
6	1.92	48	52
7	2.00	61	39
8	2.03	95	5
9	2.09	46	54
10	2.10	43	57
11	2.12	16	84
12	2.12	23	77
13	2.13	28	72
14	2.18	6	94
15	2.30	3	97
16	2.34	5	95
17	2.35	0	100
18	2.36	2	98
19	2.37	7	93
20	2.38	10	90

Supplementary Table 20: Energies (in eV) of adiabatic triplet (T_i) states and their diabatic composition in terms of intra (in blue) and intermolecular (in red) contributions and Z_i states: $Z_{inter} = Z_{9\text{-}20}$ and $Z_{intra} = Z_{1\text{-}8}$.

	e-h radius
Z_1	3.969
Z_2	3.972
Z_3	3.420
Z_4	3.438
Z_5	3.616
Z_6	3.707
Z_7	3.443
Z_8	3.455
Z_9	4.045
Z_{10}	4.026
Z_{11}	4.170
Z_{12}	3.886
Z_{13}	3.877
Z_{14}	4.171
Z_{15}	9.313
Z_{16}	9.476
Z_{17}	8.870
Z_{18}	8.765
Z_{19}	6.934
Z_{20}	6.905

	e-h radius	Z_{intra}	Z_{inter}
T_1	3.918	79	21
T_2	4.070	83	17
T_3	3.895	94	6
T_4	3.633	98	2
T_5	3.938	54	46
T_6	4.136	48	52
T_7	3.887	61	39
T_8	3.569	95	5
T_9	5.459	46	54
T_{10}	5.321	43	57
T_{11}	4.263	16	84
T_{12}	4.644	23	77
T_{13}	4.352	28	72
T_{14}	4.635	6	94
T_{15}	7.904	3	97
T_{16}	6.168	5	95
T_{17}	7.487	0	100
T_{18}	6.888	2	98
T_{19}	7.947	7	93
T_{20}	7.344	10	90

Supplementary Table 21: Electron-hole (e-h) radius (in Å) for the diabatic (left) and adiabatic (right) triplet states. Intra and intermolecular contributions are indicated in blue and red, respectively.

state	ΔE	A e-	A h+	B e-	B h+	C e-	C h+	D e-	D h+
Z_1	1.81	0.000	0.000	-0.004	0.002	-0.021	0.028	-0.973	0.970
Z_2	1.81	-0.973	0.969	-0.021	0.028	-0.004	0.002	0.000	0.000
Z_3	1.85	-0.021	0.018	-0.925	0.932	-0.051	0.048	-0.003	0.002
Z_4	1.85	-0.003	0.002	-0.050	0.052	-0.926	0.929	-0.020	0.017
Z_5	1.91	-0.008	0.004	-0.057	0.032	-0.910	0.924	-0.023	0.039
Z_6	1.92	-0.023	0.043	-0.900	0.920	-0.067	0.033	-0.008	0.003
Z_7	2.00	0.000	0.000	-0.001	0.000	-0.026	0.020	-0.971	0.979
Z_8	2.00	-0.969	0.977	-0.028	0.021	-0.001	0.000	0.000	0.000
Z_9	2.10	-0.034	0.816	-0.783	0.141	-0.135	0.041	-0.046	0.001
Z_{10}	2.10	-0.035	0.001	-0.141	0.031	-0.787	0.151	-0.036	0.816
Z_{11}	2.11	-0.004	0.027	-0.037	0.061	-0.075	0.887	-0.883	0.025
Z_{12}	2.11	-0.088	0.003	-0.773	0.068	-0.128	0.727	-0.010	0.201
Z_{13}	2.11	-0.011	0.193	-0.119	0.744	-0.782	0.059	-0.085	0.002
Z_{14}	2.11	-0.875	0.027	-0.087	0.887	-0.033	0.068	-0.004	0.017
Z_{15}	2.27	-0.857	0.036	-0.115	0.711	-0.024	0.240	-0.003	0.013
Z_{16}	2.27	-0.002	0.013	-0.016	0.225	-0.103	0.725	-0.878	0.036
Z_{17}	2.27	-0.001	0.777	-0.015	0.214	-0.296	0.008	-0.687	0.000
Z_{18}	2.28	-0.648	0.000	-0.333	0.007	-0.016	0.197	-0.002	0.795
Z_{19}	2.29	-0.001	0.318	-0.011	0.648	-0.314	0.033	-0.672	0.000
Z_{20}	2.30	-0.701	0.001	-0.285	0.035	-0.010	0.679	-0.002	0.284

Supplementary Table 22: Diabatic states excitation energies (in eV) and e-/h+ contributions to the relative Mulliken fragment charges of diabatic states of the BF2 tetramer with respect to the ground state charge distribution. Intra and intermolecular contributions are indicated in blue and red, respectively.

ω	Z_1	Z_2	Z_3	Z_4	Z_5	Z_6	Z_7	Z_8	Z_9	Z_{10}	Z_{11}	Z_{12}	Z_{13}	Z_{14}	Z_{15}	Z_{16}	Z_{17}	Z_{18}	Z_{19}	Z_{20}
T_1	4	5	19	18	16	16	1	1	2	1	2	5	5	2	2	2	0	0	0	0
T_2	24	25	7	8	5	5	5	5	3	3	3	0	0	3	2	2	0	0	0	0
T_3	34	31	4	4	3	3	8	7	1	1	0	1	1	0	0	1	0	0	0	0
T_4	12	14	22	22	11	10	3	4	1	1	0	0	0	0	0	0	0	0	0	0
T_5	1	1	12	12	13	12	1	1	1	1	9	11	11	10	0	0	1	1	0	0
T_6	2	3	9	8	9	9	4	4	3	3	18	2	2	18	1	2	0	0	2	2
T_7	5	5	3	3	2	2	21	20	13	12	3	3	3	3	1	1	0	0	0	0
T_8	4	4	9	9	10	9	24	26	2	2	0	0	0	0	0	0	0	0	0	0
T_9	0	1	0	1	11	2	21	9	4	1	13	0	0	3	6	14	8	2	3	1
T_{10}	0	0	2	0	5	17	4	14	0	2	9	3	0	12	12	2	2	9	4	5
T_{11}	0	0	3	2	5	1	1	3	2	0	5	23	35	10	0	0	5	1	2	3
T_{12}	4	3	3	6	0	0	3	3	20	26	0	11	4	0	1	1	4	7	1	1
T_{13}	4	5	0	0	5	7	3	3	26	26	2	1	1	2	1	0	0	0	6	6
T_{14}	1	1	2	2	0	0	0	0	15	15	1	23	21	2	1	1	5	5	2	3
T_{15}	0	0	0	0	1	1	0	0	0	0	4	1	2	2	5	5	34	24	11	8
T_{16}	0	0	0	0	1	2	0	0	1	1	10	8	7	10	0	1	7	16	17	17
T_{17}	0	0	0	0	0	0	0	0	4	1	0	1	3	2	7	7	20	10	35	10
T_{18}	0	0	0	0	1	1	0	0	1	4	6	1	1	5	1	6	5	13	14	41
T_{19}	1	1	2	2	0	0	0	0	0	1	7	4	2	4	22	36	6	10	0	1
T_{20}	1	1	2	1	2	2	0	1	1	1	6	2	3	11	37	18	3	2	2	3

Supplementary Table 23: Diabatic contributions ω (in %) as obtained from Boys diabatization. Intra contributions are in blue and intermolecular contributions are in red.

BF2 tetramer: intra-CT/inter-CT energy diagram

To simplify the above information, we partially diagonalize the singlet and triplet Z_i diabatic states obtained for the BF2 tetramer within the intra-CT, inter-CT involving 1st neighbours and inter-CT beyond 1st neighbours.

states	intra	intra	intra	intra	inter(1st)	inter(1st)	inter(1st)	inter(1st)	inter(1st)	inter(1st)	inter(1st)	inter(1st)	inter(1st)	inter(1st)	inter	inter	inter	inter	inter	inter
intra	2400	0	0	0	3	-5	-3	2	1	5	1	15	23	-16	-84	35	5	-50	-31	20
intra	0	2388	0	0	-3	-5	4	2	-2	-6	-2	12	-23	-19	22	83	16	42	-45	-6
intra	0	0	2364	0	36	0	-31	-1	-17	-78	8	-3	-27	-13	-40	-18	-2	-3	-6	-40
intra	0	0	0	2335	5	-44	3	8	109	-18	-2	73	-8	143	15	-34	12	-4	-33	7
inter(1st)	3	-3	36	5	2499	0	0	0	0	0	0	0	0	0	-8	1	-8	14	18	75
inter(1st)	-5	-5	0	-44	0	2397	0	0	0	0	0	0	0	0	-23	47	-6	-5	-39	6
inter(1st)	-3	4	-31	3	0	0	2323	0	0	0	0	0	0	0	-15	-9	-4	-6	14	67
inter(1st)	2	2	-1	8	0	0	0	2310	0	0	0	0	0	0	-47	95	-4	-3	-21	5
inter(1st)	1	-2	-17	109	0	0	0	0	2182	0	0	0	0	0	-4	-7	-22	0	8	-1
inter(1st)	5	-6	-78	-18	0	0	0	0	0	2170	0	0	0	0	-29	-13	0	-19	6	38
inter(1st)	1	-2	8	-2	0	0	0	0	0	0	2121	0	0	0	134	61	1	15	0	18
inter(1st)	15	12	-3	73	0	0	0	0	0	0	0	2119	0	0	-16	36	87	3	61	-11
inter(1st)	23	-23	-27	-8	0	0	0	0	0	0	0	0	2055	0	-9	-5	8	106	-22	-54
inter(1st)	-16	-19	-13	143	0	0	0	0	0	0	0	0	0	1948	-33	66	49	-7	-42	5
inter	-84	22	-40	15	-8	-23	-15	-47	-4	-29	134	-16	-9	-33	2329	0	0	0	0	0
inter	35	83	-18	-34	1	47	-9	95	-7	-13	61	36	-5	66	0	2317	0	0	0	0
inter	5	16	-2	12	-8	-6	-4	-4	-22	0	1	87	8	49	0	0	2312	0	0	0
inter	-50	42	-3	-4	14	-5	-6	-3	0	-19	15	3	106	-7	0	0	0	2295	0	0
inter	-31	-45	-6	-33	18	-39	14	-21	8	6	0	61	-22	-42	0	0	0	0	2278	0
inter	20	-6	-40	7	75	6	67	5	-1	38	18	-11	-54	5	0	0	0	0	0	2248

Supplementary Table 24: Diabatic energies (diagonal) and electronic couplings (off-diagonal) between the lowest intra-^{1}CT (blue), 1st neighbours inter-^{1}CT (red) and inter-^{1}CT beyond 1st neighbours (green). All values are expressed in meV.

states	intra	intra	intra	intra	intra	intra	intra	intra	inter(1st)	inter(1st)	inter(1st)	inter(1st)	inter(1st)	inter(1st)	inter(1st)	inter(1st)	inter	inter	inter	inter
intra	2052	0	0	0	0	0	0	0	37	-25	-11	12	25	-43	-37	-24	5	-10	5	-1
intra	0	2050	0	0	0	0	0	0	-30	-32	12	12	-35	-32	39	-25	11	-3	-2	-4
intra	0	0	2022	0	0	0	0	0	45	-10	16	2	32	-2	-64	-15	-3	-2	9	5
intra	0	0	0	2005	0	0	0	0	-13	93	3	-29	5	-34	0	125	4	-3	4	-3
intra	0	0	0	0	1788	0	0	0	3	-18	3	61	-8	-56	5	43	8	-7	4	-7
intra	0	0	0	0	0	1752	0	0	45	1	-65	22	56	-12	56	9	-5	-7	-18	-7
intra	0	0	0	0	0	0	1751	0	0	49	-8	-112	16	37	5	-65	-5	2	4	-13
intra	0	0	0	0	0	0	0	1723	-48	-7	-110	-9	-165	10	-105	0	-12	-15	-23	-11
inter(1st)	37	-30	45	-13	3	45	0	-48	2344	0	0	0	0	0	0	0	3	2	2	0
inter(1st)	-25	-32	-10	93	-18	1	49	-7	0	2341	0	0	0	0	0	0	0	0	-2	4
inter(1st)	-11	12	16	3	3	-65	-8	-110	0	0	2177	0	0	0	0	0	4	1	52	24
inter(1st)	12	12	2	-29	61	22	-112	-9	0	0	0	2111	0	0	0	0	61	-58	8	-13
inter(1st)	25	-35	32	5	-8	56	16	-165	0	0	0	0	2079	0	0	0	-57	-59	36	17
inter(1st)	-43	-32	-2	-34	-56	-12	37	10	0	0	0	0	0	2069	0	0	-10	21	2	-3
inter(1st)	-37	39	-64	0	5	56	5	-105	0	0	0	0	0	0	2032	0	18	22	87	39
inter(1st)	-24	-25	-15	125	43	9	-65	0	0	0	0	0	0	0	0	2019	-11	8	45	-97
inter	5	11	-3	4	8	-5	-5	-12	3	0	4	61	-57	-10	18	-11	2326	0	0	0
inter	-10	-3	-2	-3	-7	-7	2	-15	2	0	1	-58	-59	21	22	8	0	2319	0	0
inter	5	-2	9	4	4	-18	4	-23	2	-2	52	8	36	2	87	45	0	0	2252	0
inter	-1	-4	5	-3	-7	-7	-13	-11	0	4	24	-13	17	-3	39	-97	0	0	0	2247

Supplementary Table 25: Diabatic energies (diagonal) and electronic couplings (off-diagonal) between the lowest intra-^{3}CT (blue), 1st neighbours inter-^{3}CT (red) and inter-^{3}CT beyond 1st neighbours (green). All values are expressed in meV.

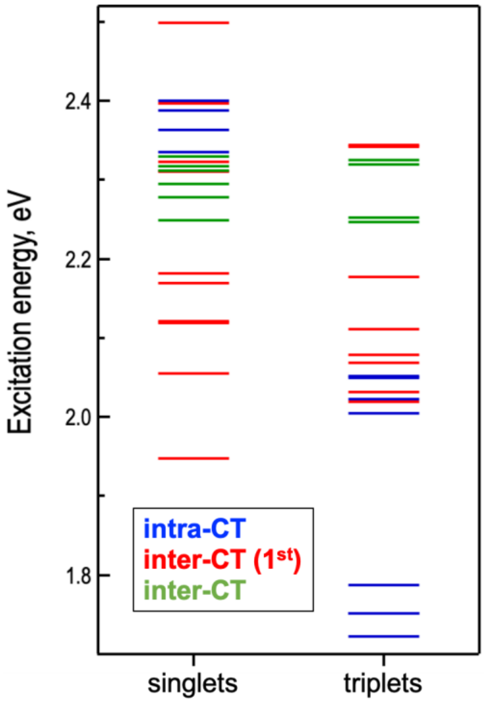

Supplementary Figure 37: Singlet and triplet intra-CT and inter-CT diabatic state energy distribution for the BF2 tetramer. Blue: intra-CT; red: inter-CT (1st neighbours); green: inter-CT (beyond 1st neighbours).

4CzIPN dimer structure

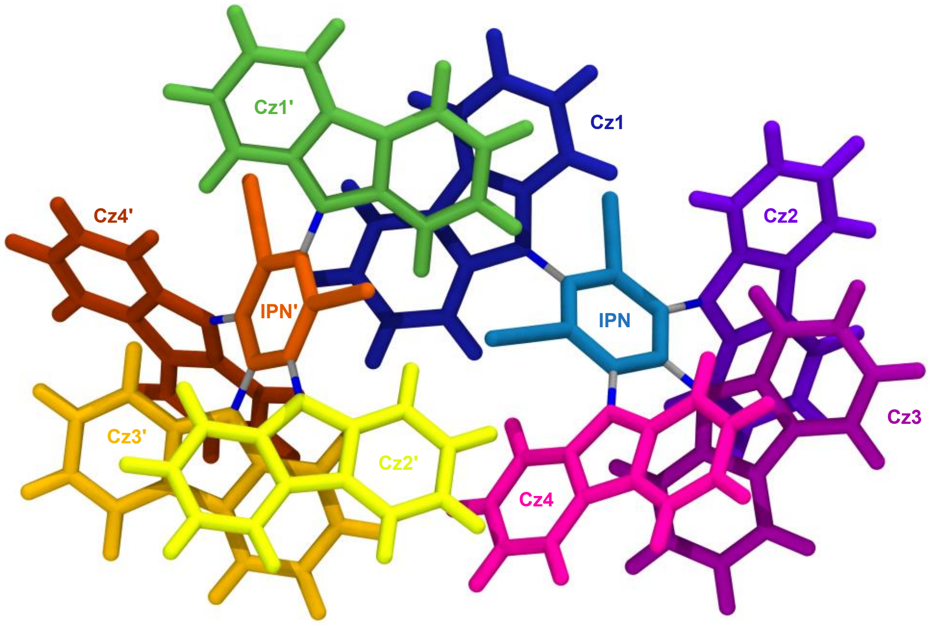

Supplementary Figure 38: Optimised structure of the ground state 4CzIPN dimer and nomenclature of the fragments considered in the diabatization analysis.

4CzIPN monomer: singlet states

Diabatization of singlet excited states of the 4CzIPN monomer: Z_i are D→A diabatic contributions, respectively. D = Cz; A = IPN.

i	S_i	Z_i
1	2.88 (0.09)	3.03
2	3.07 (0.04)	3.03
3	3.09 (0.01)	3.05
4	3.13 (0.02)	3.06

ω	Z_1	Z_2	Z_3	Z_4
S_1	28	27	20	25
S_2	51	49	0	0
S_3	7	9	79	4
S_4	14	14	0	71

Supplementary Table 26: (left) Energies of adiabatic (S_i) and diabatic (Z_i) states (in eV, oscillator strength in parenthesis); (right) Diabatic contributions ω (in %) as obtained from Boys diabatization for the four lowest singlet states of the 4CzIPN monomer.

State	IPN	Cz1	Cz2	Cz3	Cz4
Z_1	-0.836	-0.002	-0.029	0.058	0.808
Z_2	-0.836	-0.002	0.808	0.059	-0.029
Z_3	-0.845	-0.002	0.028	0.792	0.027
Z_4	-0.851	0.918	-0.033	-0.002	-0.033

Supplementary Table 27: Relative Mulliken fragment charges of diabatic states of the 4CzIPN monomer with respect to the ground state charge distribution.

The four lowest excited states S_{1-4} correspond to combinations of the intra-CT diabatic states (from each Cz to the central IPN unit).

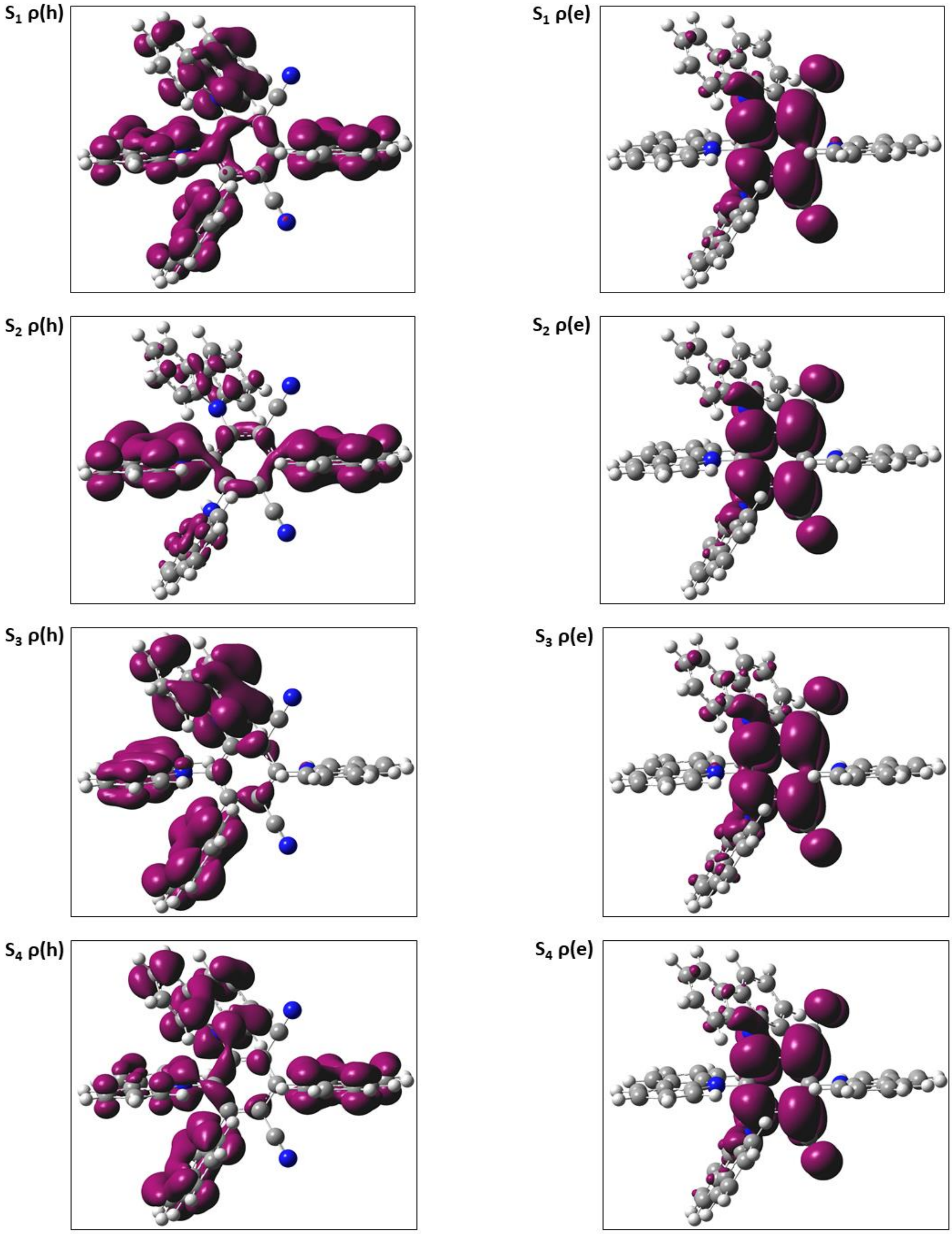

Supplementary Figure 39: Hole (ρ(h)) and electron (ρ(e)) density plots of the four lowest excited states $S_{1\text{-}4}$ of the 4CzIPN monomer.

4CzIPN dimer, $d_{\perp}$ = 3.5 Å

i	S_i	intra	inter	T_i	intra	inter
1	2.52 (0.04)	78	22	2.36	98	2
2	2.59 (0.05)	100	0	2.41	100	0
3	2.64 (0.01)	16	84	2.56	94	6
4	2.72 (0.02)	56	44	2.59	100	0
5	2.77 (0.02)	100	0	2.62	50	50
6	2.80 (0.01)	49	51	2.66	57	43
7	2.84 (0.06)	99	1	2.69	91	9
8	2.85 (0.00)	81	19	2.70	99	1
9	2.85 (0.07)	81	19	2.73	94	6
10	2.88 (0.00)	10	90	2.76	100	0
11	2.90 (0.01)	61	39	2.79	100	0
12	2.91 (0.02)	100	0	2.82	19	81
13	2.92 (0.18)	98	2	2.86	82	18
14	2.97 (0.06)	89	11	2.88	26	74
15	2.98 (0.01)	93	7	2.89	90	10
16	2.98 (0.09)	88	12	2.90	100	0

Supplementary Table 28: Energies (in eV) of adiabatic singlet (S_i) (oscillator strength in parenthesis) and triplet (T_i) states. Intra and intermolecular contributions are indicated in blue and red, respectively.

i	S_i	T_i
1	4.329	3.742
2	3.636	3.497
3	7.612	3.138
4	5.469	2.966
5	3.759	5.422
6	5.688	5.704
7	3.290	2.730

Supplementary Table 29: Electron-hole (e-h) radius (in Å) for the adiabatic singlet (S_i) and triplet (T_i) states.

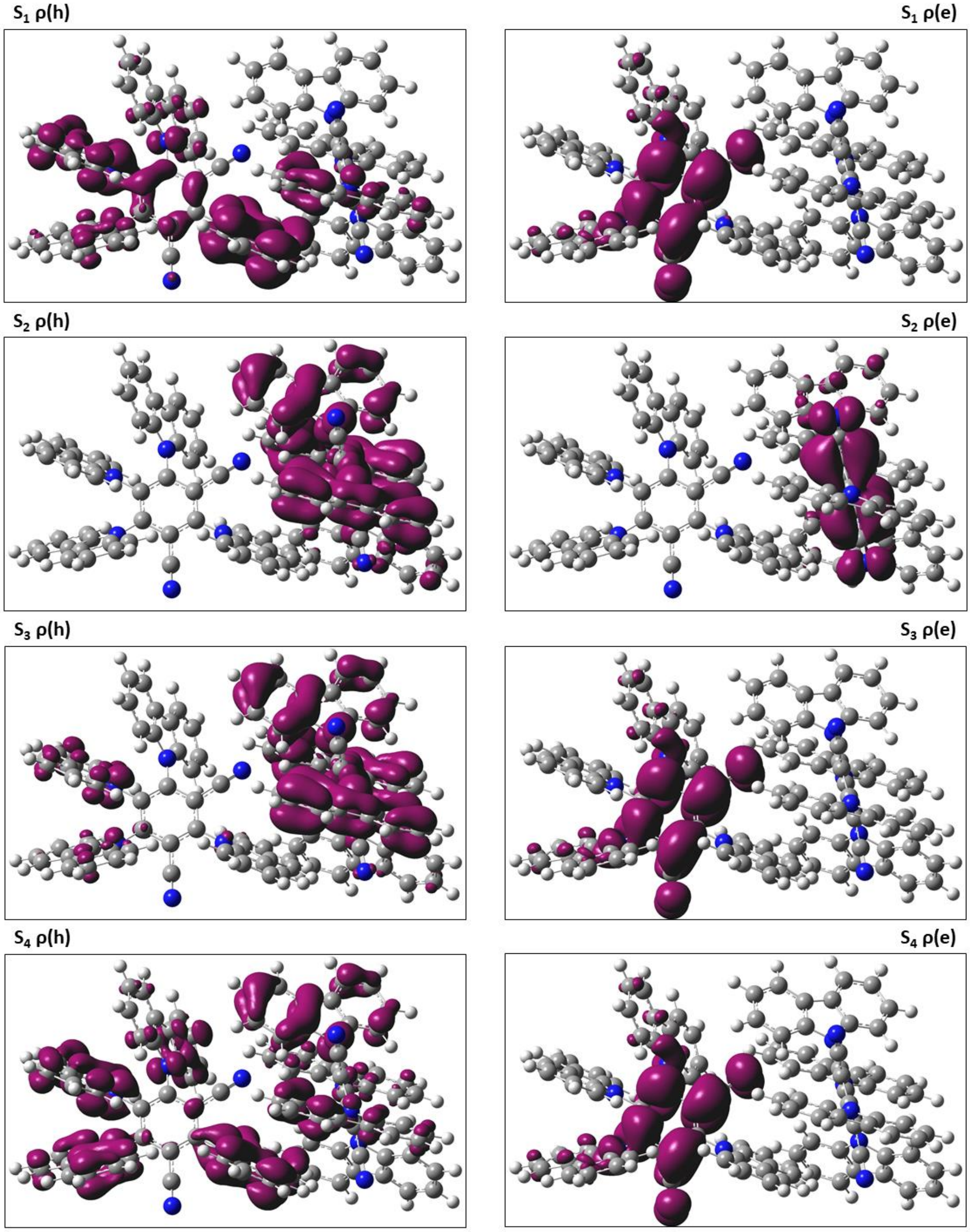

Supplementary Figure 40: Hole (ρ(h)) and electron (ρ(e)) density plots from S_1 to S_4 of the 4CzIPN dimer at $d_{\perp} = 3.5$ Å.

4CzIPN diabatization at $d_{\perp}$ = 3.5 Å: singlet states

	IPN	Cz1	Cz2	Cz3	Cz4	IPN'	Cz1'	Cz2'	Cz3'	Cz4'
Z_1 (2.72)	-0.88	0.01	-0.06	0.00	-0.05	0.07	0.83	0.01	0.06	0.01
Z_2 (2.74)	0.00	0.01	0.00	0.00	0.00	-0.81	0.90	-0.05	0.00	-0.04
Z_3 (2.75)	-0.83	0.92	-0.05	0.00	-0.04	0.00	0.01	0.00	0.00	0.00
Z_4 (2.77)	-0.83	0.00	-0.02	0.70	0.16	0.00	0.00	0.00	0.00	0.00
Z_5 (2.77)	0.00	0.00	0.00	0.00	0.00	-0.81	0.00	0.77	0.04	0.00
Z_6 (2.78)	-0.87	0.00	-0.06	0.00	-0.04	0.06	0.01	0.79	0.07	0.03
Z_7 (2.79)	-0.81	0.00	0.67	0.08	0.05	0.00	0.00	0.00	0.00	0.00
Z_8 (2.79)	-0.84	0.00	0.25	0.51	0.07	0.00	0.00	0.00	0.00	0.00
Z_9 (2.80)	0.00	0.00	0.00	0.00	0.00	-0.86	0.00	0.53	0.34	0.00
Z_{10} (2.81)	-0.87	-0.01	-0.06	0.00	-0.05	0.01	0.00	0.71	0.27	0.00
Z_{11} (2.82)	0.00	0.00	0.00	0.00	0.00	-0.82	0.00	-0.03	0.65	0.21
Z_{12} (2.85)	-0.86	0.23	-0.04	0.00	-0.01	0.00	0.67	0.00	0.01	0.00
Z_{13} (2.88)	-0.81	0.52	-0.02	0.00	0.19	0.00	0.06	0.01	0.04	0.01
Z_{14} (2.92)	-0.76	0.47	0.03	0.10	-0.01	0.00	0.16	0.00	0.01	0.01
Z_{15} (2.96)	0.01	0.16	0.00	0.01	0.00	-0.88	0.80	-0.05	0.00	-0.05
Z_{16} (2.97)	-0.03	0.01	0.00	0.00	0.01	-0.73	0.36	0.18	0.18	0.01

Supplementary Table 30: Relative Mulliken fragment charges of diabatic states (energy in eV in parenthesis) of the 4CzIPN dimer with respect to the ground state charge distribution.

The lowest diabatic state Z_1 corresponds to inter-CT excitation from Cz1’ to IPN. The Z_2/Z_3 quasi-degenerate states correspond to intra-CT from Cz1 (Cz1’) to IPN (IPN’). The next state with inter-CT character Z_6 involves a transfer of the electron density from Cz2’ to IPN. Diabatic states Z_{12}-Z_{15} have mixed inter-/intra-CT character with Z_{12} being mostly inter-CT while Z_{13}-Z_{15} correspond primarily to local excitations.

ω	Z_1	Z_2	Z_3	Z_4	Z_5	Z_6	Z_7	Z_8	Z_9	Z_{10}	Z_{11}	Z_{12}	Z_{13}	Z_{14}	Z_{15}	Z_{16}
S_1	10	0	31	17	0	1	9	5	0	1	0	9	14	1	0	0
S_2	0	32	0	0	25	0	0	0	14	0	27	0	0	0	2	0
S_3	37	0	0	5	0	30	4	5	0	17	0	0	1	0	0	0
S_4	6	0	13	14	0	13	20	8	0	11	0	13	1	0	0	0
S_5	0	49	0	0	25	0	0	1	20	0	0	0	0	0	5	0
S_6	31	0	1	2	0	5	2	33	1	10	0	5	9	0	0	0
S_7	0	0	0	31	1	0	57	7	2	0	0	0	1	0	0	0
S_8	6	0	0	15	18	2	0	13	33	3	0	9	0	1	0	0
S_9	4	0	0	9	15	0	4	25	27	7	0	7	0	0	0	0
S_{10}	0	0	6	0	0	37	0	0	0	47	0	5	2	1	0	0
S_{11}	6	0	47	2	0	11	0	0	0	3	0	20	7	5	0	0
S_{12}	0	8	0	0	12	0	0	0	3	0	69	0	0	2	5	0
S_{13}	0	0	1	0	2	0	0	0	0	0	1	2	5	85	1	2
S_{14}	0	1	0	3	0	0	2	1	0	0	1	11	15	4	10	51
S_{15}	0	7	0	1	0	0	1	0	0	0	2	7	15	0	66	0
S_{16}	0	1	0	1	0	0	0	0	0	0	0	12	29	0	11	45

Supplementary Table 31: Diabatic contributions ω (in %) as obtained from Boys diabatization.

4CzIPN diabatization at $d_{\perp}$ = 3.5 Å: triplet states

	IPN	Cz1	Cz2	Cz3	Cz4	IPN'	Cz1'	Cz2'	Cz3'	Cz4'
Z_1 (2.56)	0.00	0.00	0.00	0.00	0.00	-0.75	0.00	0.78	0.00	-0.04
Z_2 (2.60)	-0.73	0.00	0.77	0.00	-0.04	0.00	0.00	0.00	0.00	0.00
Z_3 (2.62)	-0.79	0.87	-0.05	0.00	-0.04	0.00	0.01	0.00	0.00	0.00
Z_4 (2.65)	-0.65	0.46	0.03	0.12	0.02	0.00	0.03	0.00	0.00	0.00
Z_5 (2.65)	0.00	0.02	0.00	0.00	0.00	-0.79	0.85	-0.05	0.00	-0.04
Z_6 (2.65)	-0.72	0.00	-0.05	0.01	0.75	0.00	0.00	0.00	0.00	0.00
Z_7 (2.66)	-0.78	0.00	-0.04	0.76	0.06	0.00	0.00	0.00	0.00	0.00
Z_8 (2.70)	0.00	0.00	0.00	0.00	0.00	-0.64	0.39	0.08	0.15	0.02
Z_9 (2.72)	-0.88	0.00	-0.06	0.00	-0.05	0.07	0.82	0.02	0.06	0.02
Z_{10} (2.73)	0.00	0.00	0.00	0.00	0.00	-0.77	0.00	-0.04	0.78	0.04
Z_{11} (2.75)	0.00	0.00	0.00	0.00	0.00	-0.71	0.00	-0.04	0.01	0.74
Z_{12} (2.77)	-0.84	0.00	0.42	0.43	0.00	0.00	0.00	0.00	0.00	0.00
Z_{13} (2.79)	0.00	0.00	0.00	0.00	0.00	-0.85	0.00	0.66	0.23	-0.03
Z_{14} (2.79)	-0.87	0.00	-0.06	0.00	-0.05	0.06	0.01	0.81	0.07	0.04
Z_{15} (2.80)	-0.87	-0.01	-0.06	0.00	-0.05	0.01	0.00	0.71	0.27	0.00
Z_{16} (2.82)	-0.87	0.68	-0.06	0.00	-0.05	0.00	0.30	0.00	0.00	0.00

Supplementary Table 32: Relative Mulliken fragment charges of diabatic states (energy in eV in parenthesis) of the 4CzIPN dimer with respect to the ground state charge distribution.

ω	Z_1	Z_2	Z_3	Z_4	Z_5	Z_6	Z_7	Z_8	Z_9	Z_{10}	Z_{11}	Z_{12}	Z_{13}	Z_{14}	Z_{15}	Z_{16}
**T_1**	0	15	35	0	0	7	27	0	1	0	0	8	0	0	0	5
**T_2**	38	0	0	0	25	0	0	1	0	25	3	0	8	0	0	0
**T_3**	0	36	2	23	0	28	1	0	2	0	0	4	0	3	1	1
**T_4**	37	0	0	0	18	0	0	14	0	7	22	0	1	0	0	0
**T_5**	0	3	8	20	0	5	7	0	34	0	0	3	0	10	6	3
**T_6**	0	24	14	8	0	4	1	0	8	0	0	1	0	20	15	6
**T_7**	1	1	7	45	1	27	3	4	6	0	0	1	0	2	2	2
**T_8**	10	0	0	3	16	1	1	64	1	0	2	0	2	0	0	0
**T_9**	0	16	5	0	0	17	33	0	2	0	0	19	0	2	2	3
**T_{10}**	13	0	0	0	9	0	0	1	0	19	8	0	50	0	0	0
**T_{11}**	0	0	0	0	27	0	0	17	0	4	51	0	1	0	0	0
**T_{12}**	0	0	3	0	0	5	1	0	43	0	0	0	0	10	29	8
**T_{13}**	0	2	1	0	0	4	17	0	0	0	0	49	0	6	12	9
**T_{14}**	0	1	0	0	0	2	7	0	2	0	0	13	0	41	31	3
**T_{15}**	0	1	24	0	0	0	3	0	3	0	0	1	0	6	2	60
**T_{16}**	0	0	0	0	4	0	0	0	0	44	13	0	39	0	0	0

Supplementary Table 33: Diabatic contributions ω (in %) as obtained from Boys diabatization.

Calculation of the hyperfine coupling in the BF2 dimer

In this section we present the approach developed to compute the hyperfine coupling (HFC) in the excited states of the BF2 dimer. By considering an inter-CT state involving a monomer A and a monomer B, we can depict two scenarios:

1) The electron density is fully transferred from monomer A(B) to monomer B(A) giving rise to a pure inter-CT state, where the monomer A(B) acquires a cationic nature and monomer B(A) an anionic nature.

2) The electron density is partially transferred from monomer A(B) to monomer B(A), giving rise to a hybrid intra-inter CT state, where the hole (electron) density, which we associate with the cation (anion), is delocalized over the entire dimer.

In the first case, the hole and the electron are well localized on different molecules and the local magnetic field they experience can be easily evaluated by computing the HFC on the cation and anion species, respectively. In the second case, the hole and the electron are delocalized on the two monomers and consequently the local magnetic field competing to each monomer arises from a mixed cationic and anionic character. It follows that the evaluation of the effective HFC characterizing each monomer requires the knowledge of the amount of the hole (cationic character) and the electron (anionic character) localized on monomer A and monomer B. This can be done by resorting to the *attachment/detachment* formalism, weighting the HFC of the electron (anion) and hole (cation) on the amount of attachment and detachment density localized on each monomer.

Our approach encompasses three steps:

1) The calculation of the HFC of the cation and anion of the isolated monomers by accounting for both the isotropic and dipolar part, according to the following equations[39]:

$$B_{iso} = \left[\sum_{\alpha}^{nuclei} [A_{\alpha}^2 I_{\alpha}(I_{\alpha}+1)] N_{\alpha} \right]^{\frac{1}{2}} \tag{7a}$$

$$B_{dip} = \frac{1}{3} \left[\sum_{\alpha}^{nuclei} [\boldsymbol{T}_{\alpha} \colon \boldsymbol{T}_{\alpha} I_{\alpha}(I_{\alpha}+1)]\, N_{\alpha} \right]^{\frac{1}{2}} \tag{7b}$$

where A_{α} is the isotropic constant describing the coupling between the nuclear spin of atom α and the electron spin, I_{α} is the nuclear spin, $\boldsymbol{T}_{\alpha}$ is the second-rank tensor describing the dipolar coupling between the nuclear spin of atom α and the electron spin, and N_{α} is the natural abundance of the α-th nucleus. The term $\boldsymbol{T}_{\alpha} \colon \boldsymbol{T}_{\alpha}$ is the inner product of the tensor with itself, computed as:

$$\boldsymbol{T}_{\alpha} \colon \boldsymbol{T}_{\alpha} = (T_{\alpha}^{x})^2 + \left(T_{\alpha}^{y}\right)^2 + (T_{\alpha}^{z})^2 \tag{8}$$

being T_{α}^{i} the elements of the tensor in the diagonal form.

The total effective magnetic field arising from the isotropic and dipolar HFC is then:

$$B_{HF} = \sqrt{B_{iso}^2 + B_{dip}^2} \tag{9}$$

This is done for both the monomers in the anionic and cationic form. The isotropic constant, A_α, and the dipolar tensor, $\boldsymbol{T}_\alpha$, were computed at DFT level, with the LC-ωhPBE functional using the previously mentioned parameters for the BF2 monomer and the EPR-III basis set, providing the following results:

	B_{iso} [mT]	B_{dip} [mT]	B_{HF} [mT]
Anion	0.899 (104)	0.292 (34)	0.945 (110)
Cation	0.701 (81)	0.354 (41)	0.786 (91)

Supplementary Table 34: Isotropic, dipolar and total HFC computed for the anion and cation of the BF2 monomer. The values of B_{iso} in parentheses are expressed in neV.

In our case, because the BF2 dimer is made up of two identical molecules, the HFC calculation was carried out only for one monomer. As natural abundance, we considered 0.0107 for ^{13}C, 1 for ^{1}H, 1 for ^{19}F, 0.00038 for ^{17}O, 0.99 for ^{14}N and 0.80 for ^{11}B.

2) The calculation of the attachment and detachment densities associated with the excited state of interest are carried out using the NANCY_EX package[40] from SRSH TDDFT calculations with the LC-ωhPBE functional using the previously mentioned parameters for the BF2 dimer and the 6-311+G(d,p) basis set.

3) Weighting of the HFC of the cation and anion on the percentage of detachment and attachment density localized on each monomer:

$$B_{HF,anion}^{m1\%} = B_{HF,anion}^{m1} \times Att^{m1} \qquad (10a)$$

$$B_{HF,anion}^{m2\%} = B_{HF,anion}^{m2} \times Att^{m2} \qquad (10b)$$

$$B_{HF,anion}^{dimer} = \sqrt{\left(B_{HF,anion}^{m1\%}\right)^2 + \left(B_{HF,anion}^{m2\%}\right)^2} \qquad (10c)$$

$$B_{HF,cation}^{m1\%} = B_{HF,cation}^{m1} \times Det^{m1} \qquad (11a)$$

$$B_{HF,cation}^{m2\%} = B_{HF,cation}^{m2} \times Det^{m2} \qquad (11b)$$

$$B_{HF,cation}^{dimer} = \sqrt{\left(B_{HF,cation}^{m1\%}\right)^2 + \left(B_{HF,cation}^{m2\%}\right)^2} \qquad (11c)$$

where $Att^{mi} = \sum_j^{atoms(mi)} \rho_j^{att}$ and $Det^{mi} = \sum_j^{atoms(mi)} \rho_j^{det}$ are the amount of the attachment and detachment density, respectively, localized on the *mi*-th monomer.

d⊥ = 3.5Å	**T_1**	**T_2**	**T_3**	**T_4**	**T_5**	**T_6**	**T_7**	**T_8**	**T_9**	**T_{10}**
Att^{m1}	0.499	0.501	0.500	0.499	0.498	0.502	0.496	0.504	0.499	0.501
Att^{m2}	0.501	0.499	0.500	0.500	0.502	0.498	0.504	0.496	0.501	0.499
Det^{m1}	0.499	0.501	0.501	0.499	0.502	0.498	0.505	0.495	0.499	0.501
Det^{m2}	0.501	0.498	0.499	0.500	0.498	0.502	0.495	0.505	0.501	0.499
$B_{HF,anion}^{m1\%}[mT]$	0.472	0.473	0.473	0.472	0.471	0.474	0.469	0.476	0.472	0.473
$B_{HF,anion}^{m2\%}$ $[mT]$	0.473	0.472	0.473	0.473	0.474	0.471	0.476	0.469	0.473	0.472
$B_{HF,cation}^{m1\%}[mT]$	0.392	0.394	0.394	0.392	0.395	0.391	0.397	0.389	0.392	0.394
$B_{HF,cation}^{m2\%}[mT]$	0.394	0.391	0.392	0.393	0.391	0.395	0.389	0.397	0.394	0.392
$B_{HF,anion}^{dimer}[mT]$	0.668	0.668	0.668	0.668	0.668	0.668	0.668	0.668	0.668	0.668
$B_{HF,cation}^{dimer}[mT]$	0.556	0.555	0.556	0.555	0.556	0.556	0.556	0.556	0.556	0.556
$B_{HF,anion}^{dimer}[neV]$	77	77	77	77	77	77	77	77	77	77
$B_{HF,cation}^{dimer}[neV]$	64	64	64	64	64	64	64	64	64	64

Supplementary Table 35: Attachment and detachment density localized on each BF2 monomer, the weighted hole (cation) and electron (anion) HFC according to equations 10a-10b and 11a-11b and the total hole (cation) and electron (anion) HFC in the dimer, obtained using equations 10c and 11c.

$d_{\perp}$ = 6.1Å	T_1	T_2	T_3	T_4	T_5	T_6	T_7	T_8	T_9	T_{10}
Att^{m1}	0.999	0.001	1.000	0.000	1.000	0.000	0.999	0.001	0.994	0.006
Att^{m2}	0.001	0.999	0.000	1.000	0.000	1.000	0.001	0.999	0.006	0.994
Det^{m1}	0.999	0.001	1.000	0.000	0.000	1.000	0.001	0.999	0.994	0.006
Det^{m2}	0.001	0.999	0.000	1.000	1.000	0.000	0.999	0.001	0.006	0.994
$B^{m1\%}_{HF,anion}[mT]$	0.944	0.001	0.945	0.000	0.945	0.000	0.944	0.001	0.939	0.006
$B^{m2\%}_{HF,anion}$ [mT]	0.001	0.944	0.000	0.945	0.000	0.945	0.001	0.944	0.006	0.939
$B^{m1\%}_{HF,cation}[mT]$	0.785	0.001	0.786	0.000	0.000	0.786	0.001	0.785	0.781	0.005
$B^{m2\%}_{HF,cation}[mT]$	0.001	0.785	0.000	0.786	0.786	0.000	0.785	0.001	0.005	0.781
$B^{dimer}_{HF,anion}[mT]$	0.944	0.944	0.945	0.945	0.945	0.945	0.944	0.944	0.939	0.939
$B^{dimer}_{HF,cation}[mT]$	0.785	0.785	0.786	0.786	0.786	0.786	0.785	0.785	0.781	0.781
$B^{dimer}_{HF,anion}[neV]$	109	109	110	110	110	110	109	109	109	109
$B^{dimer}_{HF,cation}[neV]$	91	91	91	91	91	91	91	91	91	91

Supplementary Table 36: Attachment and detachment density localized on each BF2 monomer, the weighted hole (cation) and electron (anion) HFC according to the equations 10a-10b and 11a-11b and the total hole (cation) and electron (anion) HFC in the dimer, obtained using equations 10c and 11c.

Calculations on the hyperfine ISC rates

Manolopoulos and co. have proposed an incoherent model to compute the rate of intersystem crossing between the singlet and triplet spin correlated radical pair (^{1}SCRP and ^{3}SCRP)[41,42], which for these purposes can be considered analogous to the inter-CT states presented here. In their model, the ^{1}SCRP is formed after photoexcitation to a locally excited singlet state (^{1}LE). Then, interconversion between ^{1}SCRP and ^{3}SCRP occurs through a hyperfine interaction-driven intersystem crossing (HFI-ISC) mechanism.

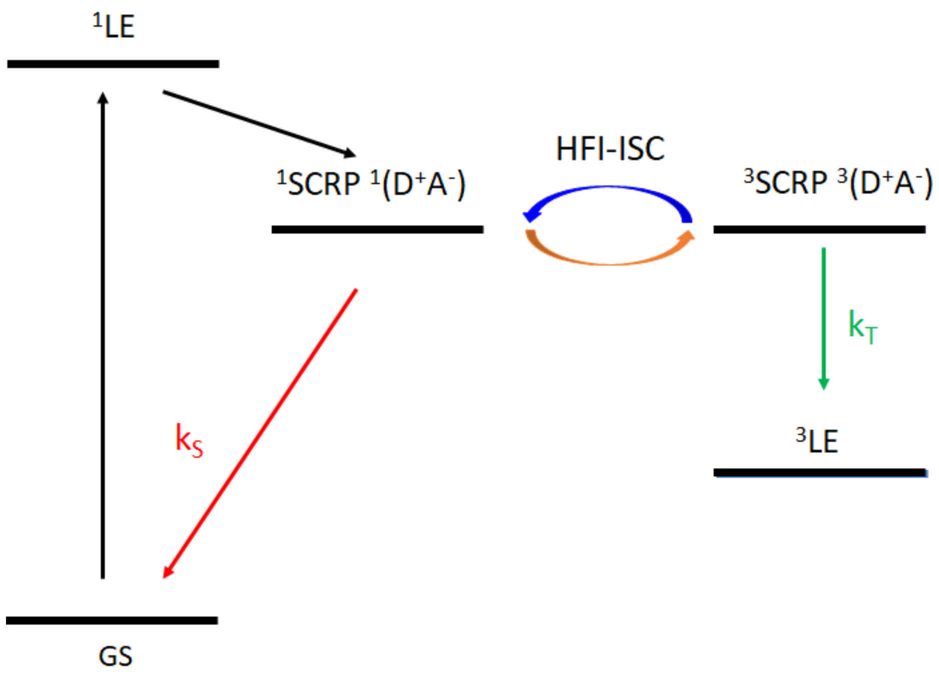

Supplementary Figure 41: Electronic structure of the different states involved in the model proposed by Manolopoulos and co. in references 36 and 37.

The HFI-ISC is limited by both the decay rate to the ground state (GS) from the ^{1}SCRP (k_S), the decay rate from ^{3}SCRP to the ^{3}LE (k_T) and the exchange energy J between the ^{1}SCRP and ^{3}SCRP.

In the incoherent, Markovian, limit, the HFI-ISC rate takes the following expression:

$$k_{HFI-ISC} = \frac{\omega_{D^+}^2 + \omega_{A^-}^2}{6} \frac{\gamma}{\gamma^2 + J^2} \tag{12}$$

where ω_{D^+} and ω_{A^-} are the pulsation associated with the hyperfine interaction corresponding to the electron donor and acceptor in their cationic and anionic states, respectively; γ is the dephasing rate evaluated as the average decay rate $\left(\gamma = \frac{k_S + k_T}{2}\right)$.

The $k_{HFI-ISC}$ rate expression is obtained through a second-order perturbation treatment and is thus valid within a range of parameters such as $\frac{\omega_{D^+}^2 + \omega_{A^-}^2}{6} \ll \gamma^2 + J^2$. $\eta = \frac{\frac{\omega_{D^+}^2 + \omega_{A^-}^2}{6}}{\gamma^2 + J^2}$ must therefore be much lower than 1 for the perturbation treatment to remain valid.

The pulsations associated with the cation and anion hyperfine interaction as well as the exchange energy are obtained from first principles DFT and TDDFT calculations. In Figure 3e, HFI-ISC rate is obtained as a function of the exchange energy J and the dephasing rate γ considering the distance dependence of the exchange energy J and γ as a parameter. Here, the J distance dependence in Supplementary Figure 34 is fitted with a decaying exponential:

$$J = \alpha \, exp(-\beta d_{\perp}) \tag{13}$$

here $d_{\perp}$ is the intermolecular distance between the ^{1}SCRP and ^{3}SCRP and α and β are fitting parameters which amount to $4.76 \cdot 10^{-3}$ eV and 0.364 $Å^{-1}$, respectively.

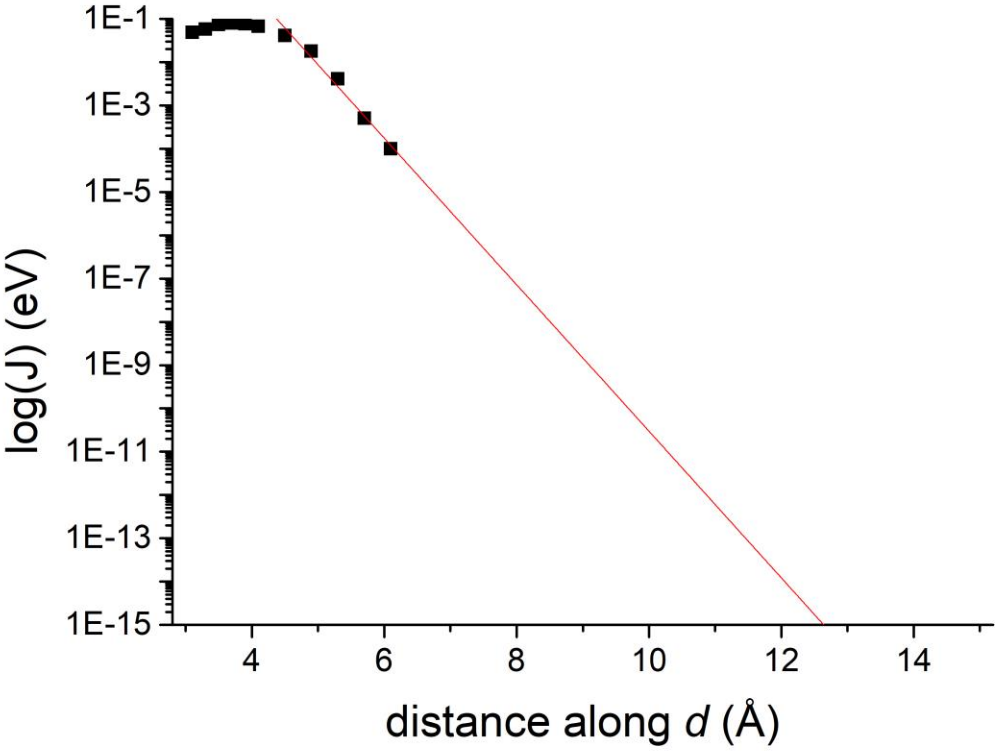

Supplementary Figure 42: Distance dependence of the exchange energy J between the ^{1}SCRP and ^{3}SCRP.

In Supplementary Figure 35, we plot η as a function of J and γ. The dephasing rate γ is estimated to be 40 ns – 1 μs from the regrowth of the SE signal in the TA of BF2 at 10 wt% in CBP (Figure 2d). Here, we note that the regrowth of the SE represents the reformation of intra-^{1}CT states, in which HFI-ISC cannot occur, from the HFI-ISC active inter-CT states. Thus, we take the onset of SE regrowth in Figure 2d (40 ns) to provide a lower bound and the point at which SE regrowth ends (1 μs) as an upper bound for the dephasing time. In the range of dephasing rates inferred from experiments and exchange energies predicted by theory, η is much larger than 1 (see highlighted blue ellipsoid area in Supplementary Figure 35) suggesting that the singlet-triplet interconversion mechanism is likely to be coherent.

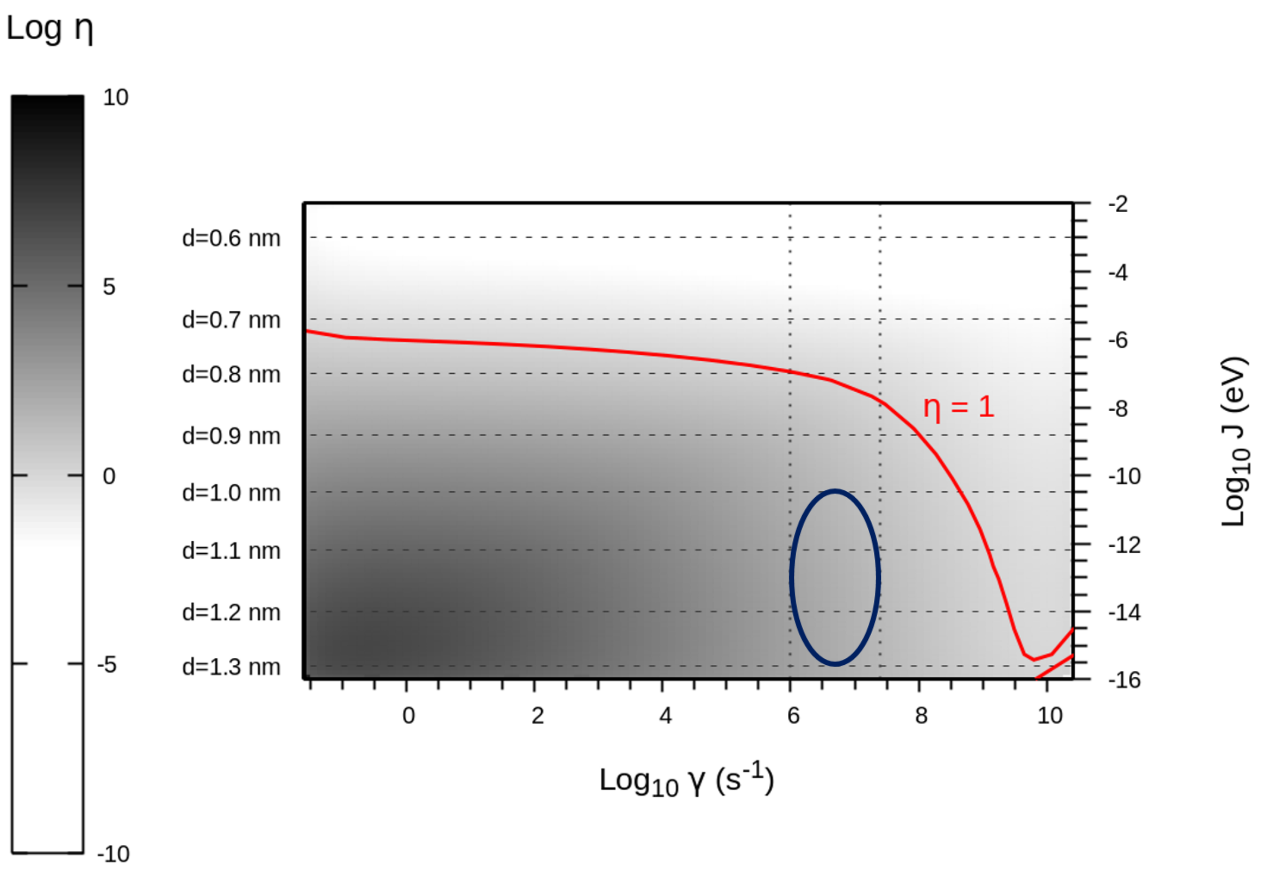

Supplementary Figure 43: η as a function of the J and γ. The circled area represents the region of interest for the BF2 system explored in our work.

Supplementary References

1. Yuan, Y. *et al.* Over 10% EQE Near-Infrared Electroluminescence Based on a Thermally Activated Delayed Fluorescence Emitter. *Adv. Funct. Mater.* **27**, 1700986 (2017).
2. Hosokai, T. *et al.* Evidence and mechanism of efficient thermally activated delayed fluorescence promoted by delocalized excited states. *Sci. Adv.* **3**, e1603282 (2017).
3. Wang, H. *et al.* Novel Thermally Activated Delayed Fluorescence Materials-Thioxanthone Derivatives and Their Applications for Highly Efficient OLEDs. *Adv. Mater.* **26**, 5198–5204 (2014).
4. Gelinas, S. *et al.* Ultrafast Long-Range Charge Separation in Organic Semiconductor Photovoltaic Diodes. *Science (80-.).* **343**, 512–516 (2014).
5. Jakowetz, A. C. *et al.* Visualizing excitations at buried heterojunctions in organic semiconductor blends. *Nat. Mater.* **16**, 551–557 (2017).
6. Scarongella, M. *et al.* A Close Look at Charge Generation in Polymer:Fullerene Blends with Microstructure Control. *J. Am. Chem. Soc.* **137**, 2908–2918 (2015).
7. Menke, S. M. *et al.* Order enables efficient electron-hole separation at an organic heterojunction with a small energy loss. *Nat. Commun.* **9**, 277 (2018).
8. Bulović, V., Deshpande, R., Thompson, M. . & Forrest, S. . Tuning the color emission of thin film molecular organic light emitting devices by the solid state solvation effect. *Chem. Phys. Lett.* **308**, 317–322 (1999).
9. Kim, D.-H. *et al.* High-efficiency electroluminescence and amplified spontaneous emission from a thermally activated delayed fluorescent near-infrared emitter. *Nat. Photonics* **12**, 98–104 (2018).

10. Tang, X. *et al.* Highly efficient luminescence from space-confined charge-transfer emitters. *Nat. Mater.* **19**, 1332–1338 (2020).

11. Cui, L.-S. *et al.* Fast spin-flip enables efficient and stable organic electroluminescence from charge-transfer states. *Nat. Photonics* **14**, 636–642 (2020).

12. Skuodis, E. *et al.* Aggregation, thermal annealing, and hosting effects on performances of an acridan-based TADF emitter. *Org. Electron.* **63**, 29–40 (2018).

13. Li, P., Ingram, G., Lee, J.-J., Zhao, Y. & Lu, Z.-H. Energy disorder and energy level alignment between host and dopant in organic semiconductors. *Commun. Phys.* **2**, 2 (2019).

14. Steiner, U. E. & Ulrich, T. Magnetic field effects in chemical kinetics and related phenomena. *Chem. Rev.* **89**, 51–147 (1989).

15. Carbonera, D. Optically detected magnetic resonance (ODMR) of photoexcited triplet states. *Photosynth. Res.* **102**, 403–414 (2009).

16. Rodgers, C. T. & Hore, P. J. Chemical magnetoreception in birds: The radical pair mechanism. *Proc. Natl. Acad. Sci.* **106**, 353–360 (2009).

17. Wang, Y., Sahin-Tiras, K., Harmon, N. J., Wohlgenannt, M. & Flatté, M. E. Immense Magnetic Response of Exciplex Light Emission due to Correlated Spin-Charge Dynamics. *Phys. Rev. X* **6**, 011011 (2016).

18. McLauchlan, K. A. & Steiner, U. E. Invited article. *Mol. Phys.* **73**, 241–263 (1991).

19. Weng, Z., Gillin, W. P. & Kreouzis, T. Fitting the magnetoresponses of the OLED using polaron pair model to obtain spin-pair dynamics and local hyperfine fields. *Sci. Rep.* **10**, 16806 (2020).

20. Xu, H., Wang, M., Yu, Z.-G., Wang, K. & Hu, B. Magnetic field effects on excited states, charge transport, and electrical polarization in organic semiconductors in spin and orbital regimes. *Adv. Phys.* **68**, 49–121 (2019).

21. Cohen, A. E. Nanomagnetic Control of Intersystem Crossing. *J. Phys. Chem. A* **113**, 11084–11092 (2009).

22. Hontz, E. *et al.* The Role of Electron–Hole Separation in Thermally Activated Delayed Fluorescence in Donor–Acceptor Blends. *J. Phys. Chem. C* **119**, 25591–25597 (2015).

23. Janssen, P. *et al.* Tuning organic magnetoresistance in polymer-fullerene blends by controlling spin reaction pathways. *Nat. Commun.* **4**, 2286 (2013).

24. Bunzmann, N. *et al.* Spin- and Voltage-Dependent Emission from Intra- and Intermolecular TADF OLEDs. *Adv. Electron. Mater.* **2000702**, 2000702 (2021).

25. Jeschke, G. Determination of the Nanostructure of Polymer Materials by Electron Paramagnetic Resonance Spectroscopy. *Macromol. Rapid Commun.* **23**, 227–246 (2002).

26. Koptyug, A. V., Saik, V. O., Animisov, O. A. & Molin, Y. N. Spin-locking in concentration-narrowed OD ESR spectra. *Chem. Phys.* **138**, 173–178 (1989).

27. Stephens, P. J., Devlin, F. J., Chabalowski, C. F. & Frisch, M. J. Ab Initio Calculation of Vibrational Absorption and Circular Dichroism Spectra Using Density Functional Force Fields. *J. Phys. Chem.* **98**, 11623–11627 (1994).

28. Archet, F. *et al.* Synthesis of Bioinspired Curcuminoid Small Molecules for Solution-Processed Organic Solar Cells with High Open-Circuit Voltage. *ACS Energy Lett.* **2**, 1303–1307 (2017).

29. Etherington, M. K. *et al.* Persistent Dimer Emission in Thermally Activated Delayed Fluorescence Materials. *J. Phys. Chem. C* **123**, 11109–11117 (2019).

30. Grimme, S., Ehrlich, S. & Goerigk, L. Effect of the damping function in dispersion corrected density functional theory. *J. Comput. Chem.* **32**, 1456–1465 (2011).

31. Hirata, S. & Head-Gordon, M. Time-dependent density functional theory within the Tamm–Dancoff approximation. *Chem. Phys. Lett.* **314**, 291–299 (1999).

32. Refaely-Abramson, S. *et al.* Gap renormalization of molecular crystals from density-functional theory. *Phys. Rev. B* **88**, 081204 (2013).

33. Gillett, A. J. *et al.* The role of charge recombination to spin-triplet excitons in non-fullerene acceptor organic solar cells. *arXiv:2010.10978 [physics.app-ph]* (2020).

34. Frisch G. W.; Schlegel, H. B.; Scuseria, G. E.; Robb, M. A.; Cheeseman, J. R.; Scalmani, G.; Barone, V.; Petersson, G. A.; Nakatsuji, H.; Li, X.; Caricato, M.; Marenich, A. V.; Bloino, J.; Janesko, B. G.; Gomperts, R.; Mennucci, B.; Hratch, D. J., M. J. . T. Gaussian 16, Rev. A.03. *Gaussian, Inc., Wallingford, CT* (2016).

35. Shao, Y. *et al.* Advances in molecular quantum chemistry contained in the Q-Chem 4 program package. *Mol. Phys.* **113**, 184–215 (2015).

36. Henderson, T. M., Izmaylov, A. F., Scalmani, G. & Scuseria, G. E. Can short-range hybrids describe long-range-dependent properties? *J. Chem. Phys.* **131**, 044108 (2009).

37. Weigend, F. & Ahlrichs, R. Balanced basis sets of split valence, triple zeta valence and quadruple zeta valence quality for H to Rn: Design and assessment of accuracy. *Phys. Chem. Chem. Phys.* **7**, 3297 (2005).

38. Turbomole V7.0 2015.

39. Yu, Z. G., Ding, F. & Wang, H. Hyperfine interaction and its effects on spin dynamics in organic solids. *Phys. Rev. B* **87**, 205446 (2013).

40. Etienne, T., Assfeld, X. & Monari, A. New Insight into the Topology of Excited States through Detachment/Attachment Density Matrices-Based Centroids of Charge. *J. Chem. Theory Comput.* **10**, 3906–3914 (2014).

41. Fay, T. P. & Manolopoulos, D. E. Radical pair intersystem crossing: Quantum dynamics or incoherent kinetics? *J. Chem. Phys.* **150**, 151102 (2019).

42. Fay, T. P., Lewis, A. M. & Manolopoulos, D. E. Spin-dependent charge recombination

along para -phenylene molecular wires. *J. Chem. Phys.* **147**, 064107 (2017).
