# Supplementary material for: Spontaneous exciton dissociation enables spin state interconversion in delayed fluorescence organic semiconductors
Source: Nat Commun. 2021 Nov 17;12:6640. doi: 10.1038/s41467-021-26689-8 (PMC8599618; doi:10.1038/s41467-021-26689-8)
Supplement: Supplementary file 3 — Reporting summary [file 41467_2021_26689_MOESM3_ESM.pdf]

nature research

Corresponding author(s): Alexander J. Gillett, Yuttapoom Puttisong, Frederic Fages, David Beljonne and Richard H. Friend

Solar Cells Reporting Summary

Nature Research wishes to improve the reproducibility of the work that we publish. This form is intended for publication with all accepted papers reporting the characterization of photovoltaic devices and provides structure for consistency and transparency in reporting. Some list items might not apply to an individual manuscript, but all fields must be completed for clarity.

For further information on Nature Research policies, including our data availability policy, see Authors & Referees.

~ Experimental design

Please check: are the following details reported in the manuscript?

1. Dimensions

Item	Yes	No	Details
Area of the tested solar cells	x		See Methods; "OSC device fabrication".
Method used to determine the device area	x		See Methods; "OSC device fabrication".

2. Current-voltage characterization

Item	Yes	No	Details
Current density-voltage (J-V) plots in both forward and backward direction	x		See Methods; "OSC device testing".
Voltage scan conditions<br>*For instance: scan direction, speed, dwell times*	x		See Methods; "OSC device testing".
Test environment<br>*For instance: characterization temperature, in air or in glove box*	x		See Methods; "OSC device testing".
Protocol for preconditioning of the device before its characterization		x	The devices were not preconditioned before characterization.
Stability of the J-V characteristic<br>*Verified with time evolution of the maximum power point or with the photocurrent at maximum power point; see ref. 7 for details.*		x	As the purpose of the devices is only to demonstrate charge generation in neat organic semiconductors, no stability measurements were performed.

3. Hysteresis or any other unusual behaviour

Item	Yes	No	Details
Description of the unusual behaviour observed during the characterization		x	No unusual behaviour.
Related experimental data		x	No unusual behaviour.

4. Efficiency

Item	Yes	No	Details
External quantum efficiency (EQE) or incident photons to current efficiency (IPCE)	x		See supplementary information.
A comparison between the integrated response under the standard reference spectrum and the response measure under the simulator		x	As the purpose of the devices is only to demonstrate charge generation in neat organic semiconductors, no comparison was performed.
For tandem solar cells, the bias illumination and bias voltage used for each subcell		x	No tandem cells reported.

5. Calibration

Item	Yes	No	Details
Light source and reference cell or sensor used for the characterization	x		See Methods; "OSC device testing".
Confirmation that the reference cell was calibrated and certified	x		See Methods; "OSC device testing".

Calculation of spectral mismatch between the reference cell and the devices under test

☐ Yes
☒ No

As the purpose of the devices is only to demonstrate charge generation in neat organic semiconductors, we did not calculate the spectral mismatch.

6. Mask/aperture

Size of the mask/aperture used during testing

☐ Yes
☒ No

As the purpose of the devices is only to demonstrate charge generation in neat organic semiconductors, we did not measure the devices with a mask.

Variation of the measured short-circuit current density with the mask/aperture area

☐ Yes
☒ No

As the purpose of the devices is only to demonstrate charge generation in neat organic semiconductors, we did not measure the devices with a mask.

7. Performance certification

Identity of the independent certification laboratory that confirmed the photovoltaic performance

☐ Yes
☒ No

As the purpose of the devices is only to demonstrate charge generation in neat organic semiconductors, the efficiency values were not certified.

A copy of any certificate(s)
Provide in Supplementary Information

☐ Yes
☒ No

No certification performed.

8. Statistics

Number of solar cells tested

☒ Yes
☐ No

See Methods; "OSC device testing".

Statistical analysis of the device performance

☒ Yes
☐ No

See Methods; "OSC device testing".

9. Long-term stability analysis

Type of analysis, bias conditions and environmental conditions
For instance: illumination type, temperature, atmosphere humidity, encapsulation method, preconditioning temperature

☐ Yes
☒ No

As the purpose of the devices is only to demonstrate charge generation in neat organic semiconductors, no stability measurements were performed.
